# Supplementary material for: Shared activity patterns arising at genetic susceptibility loci reveal underlying genomic and cellular architecture of human disease
Source: PLoS Comput Biol. 2018 Mar 1;14(3):e1005934. doi: 10.1371/journal.pcbi.1005934 (PMC5849332; doi:10.1371/journal.pcbi.1005934)
Supplement: S4 Table — (PDF) [file pcbi.1005934.s004.pdf]

Crohn's disease

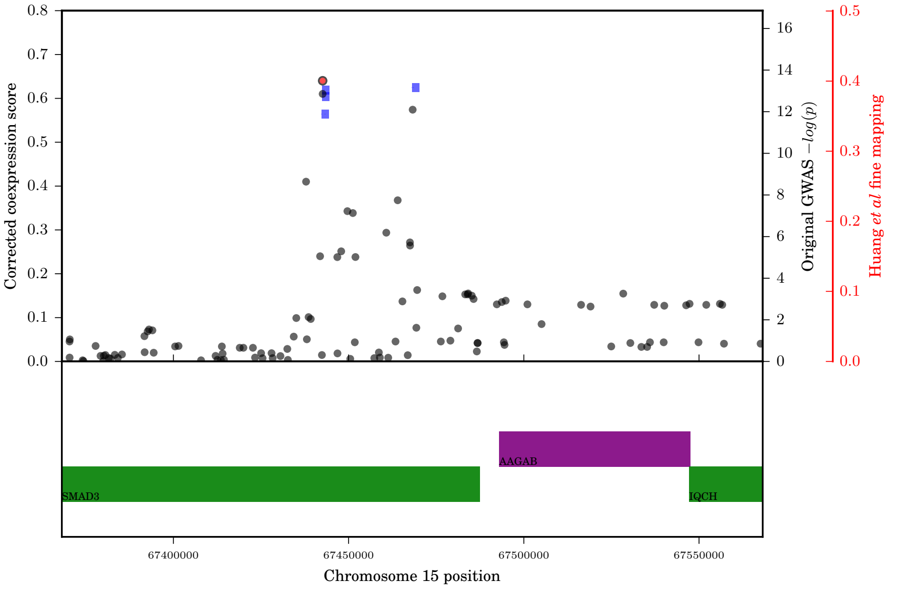

Region surrounding enhancer@chr15:67468171-67468380 [rs17294280] p(Bonferroni)=0.00e+00.

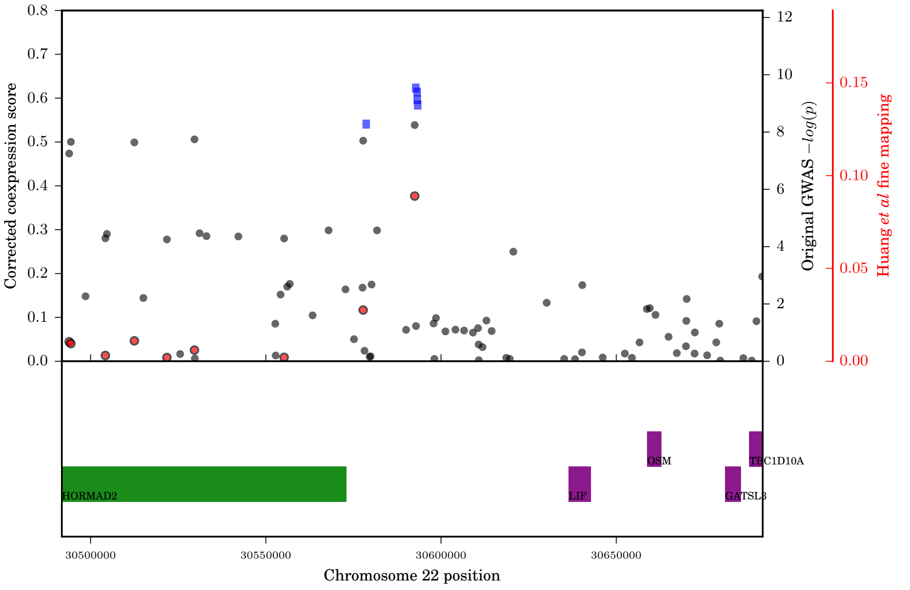

Region surrounding enhancer@chr22:30591785-30593544 [rs713875] p(Bonferroni)=0.00e+00.

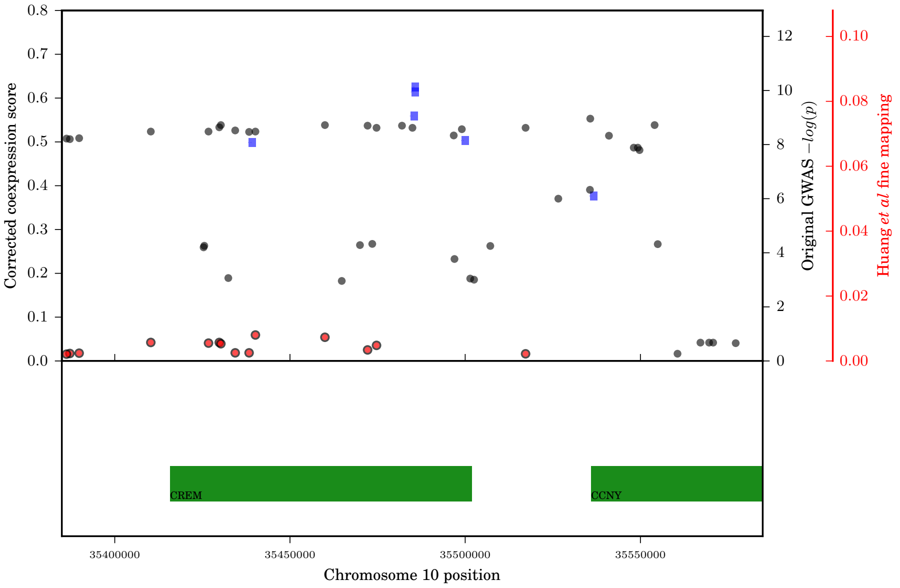

Region surrounding p@chr10:35484899..35484906,- [rs1057108] p(Bonferroni)=0.00e+00.

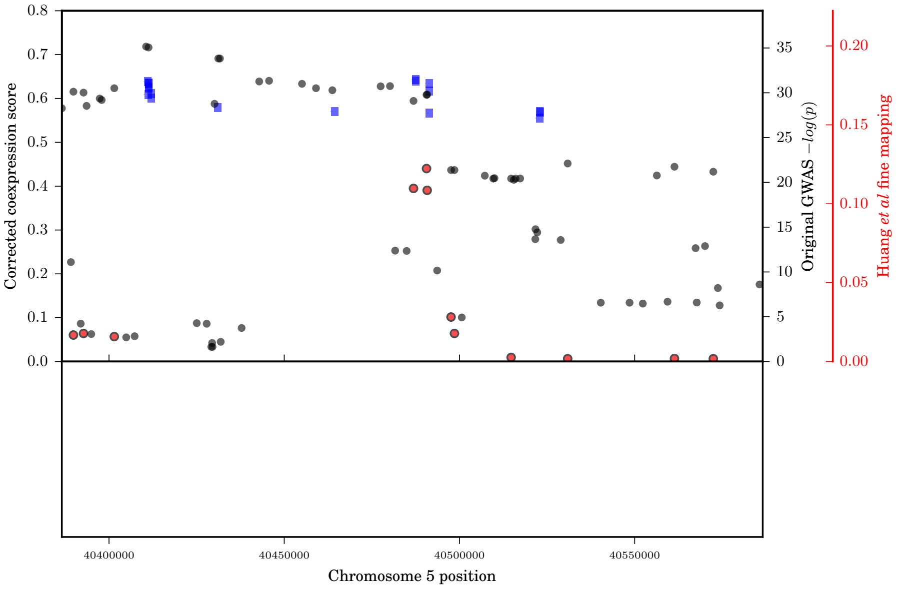

Region surrounding enhancer@chr5:40486540-40486993 [rs7720838] p(Bonferroni)=0.00e+00.

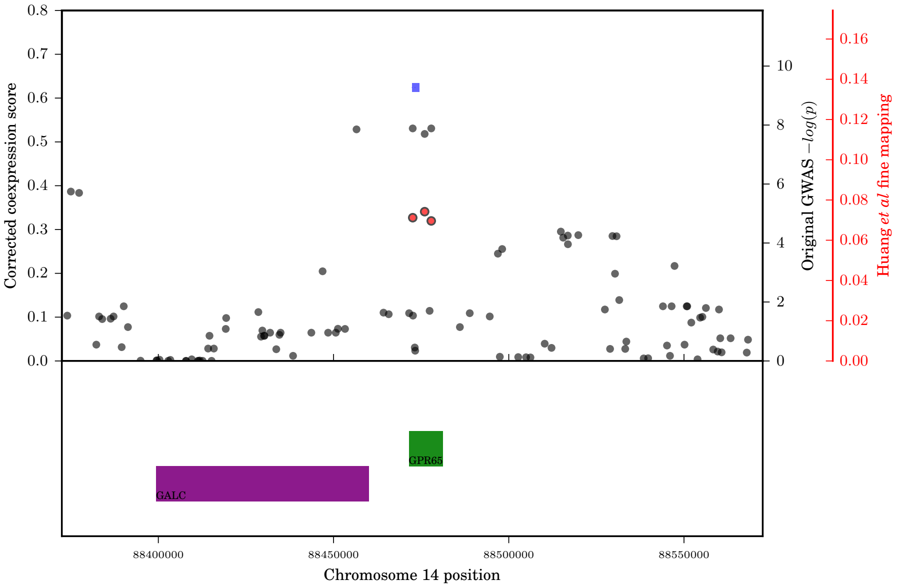

Region surrounding enhancer@chr14:88472465-88473193 [rs8005161] p(Bonferroni)=2.30e-02.

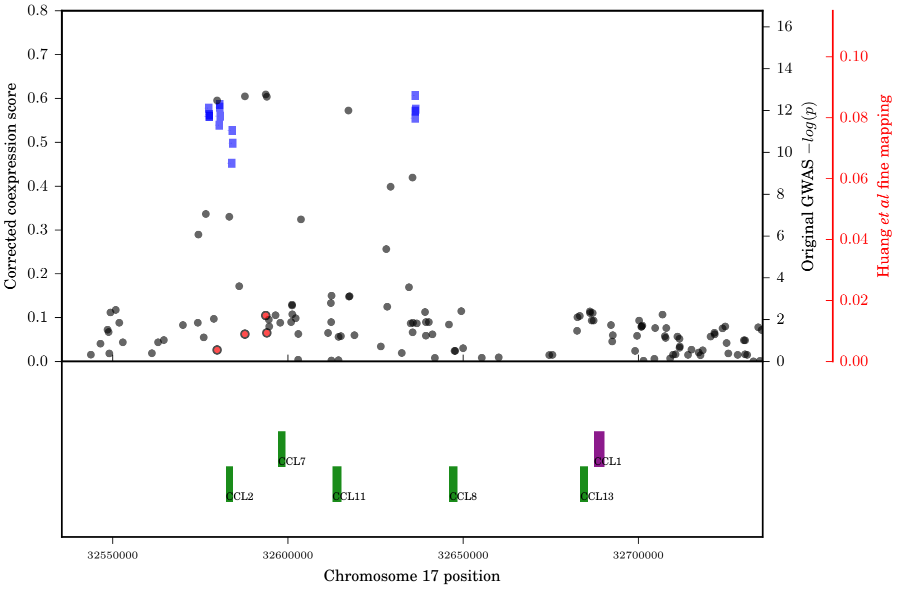

Region surrounding p@chr17:32635480..32635483,- [rs16969454] p(Bonferroni)=0.00e+00.

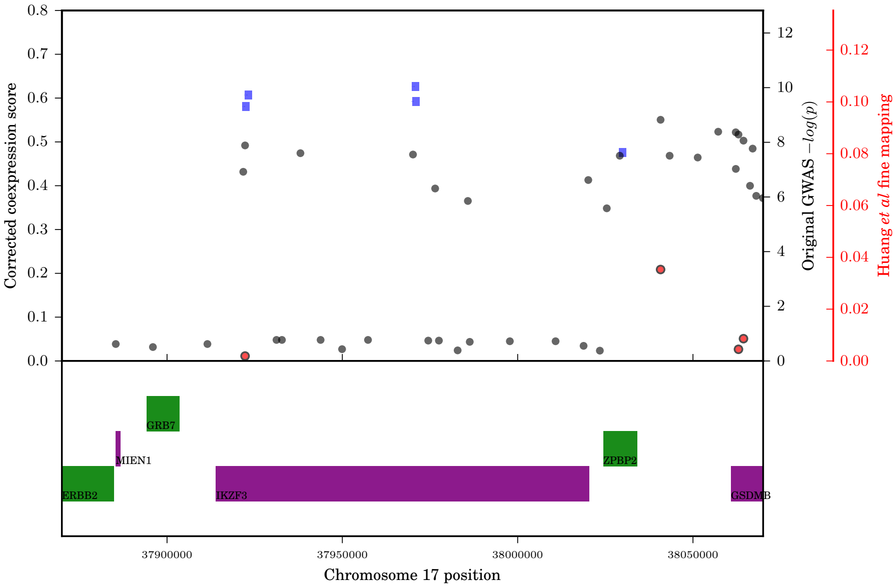

Region surrounding p@chr17:37970009..37970013,- [rs9909593] p(Bonferroni)=0.00e+00.

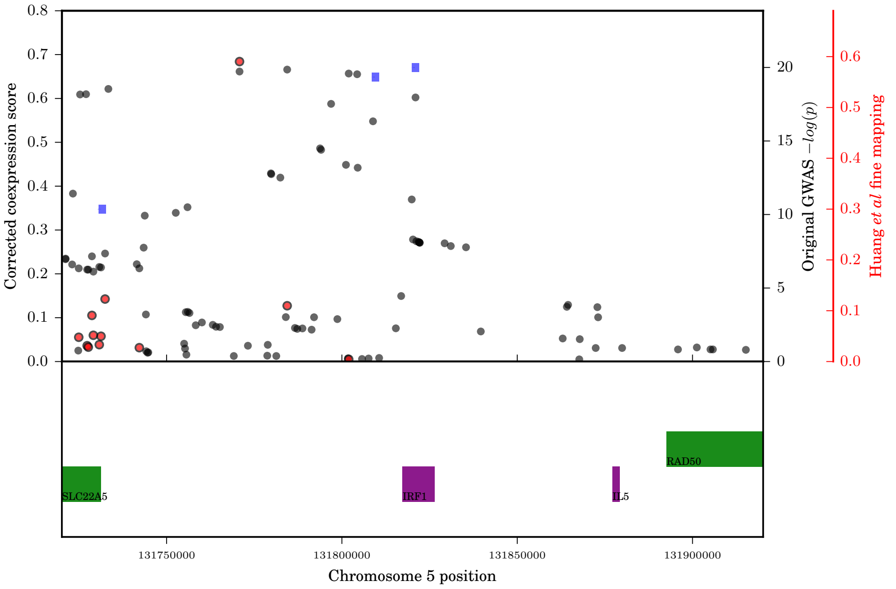

Region surrounding p@chr5:131820136..131820148,- [rs2070727] p(Bonferroni)=0.00e+00.

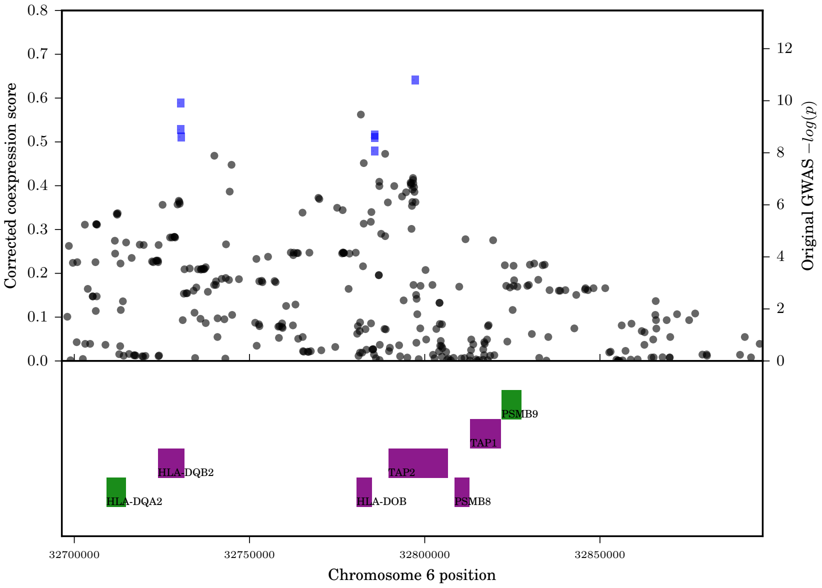

Region surrounding p4@TAP2 [rs241448—rs241447—rs241452—rs17034—rs241451—rs241449] p(Bonferroni)=0.00e+00.

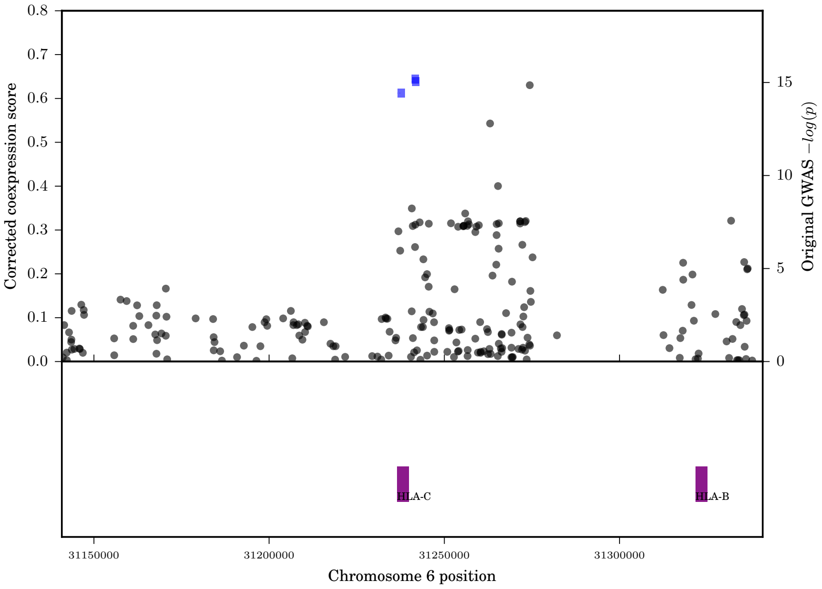

Region surrounding p@chr6:31240851..31240879,- [rs7759127] p(Bonferroni)=0.00e+00.

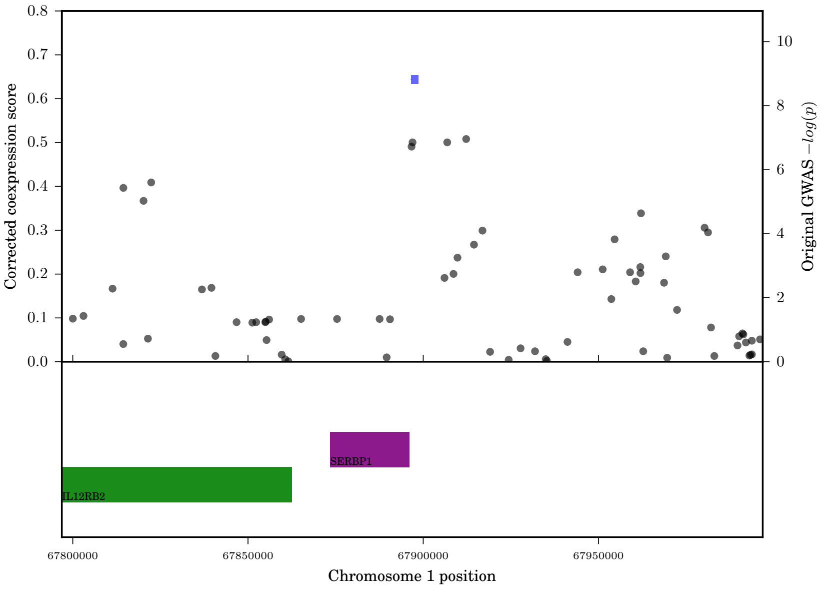

Region surrounding p@chr1:67896878..67896883,+ [rs3762313—rs3762314] p(Bonferroni)=0.00e+00.

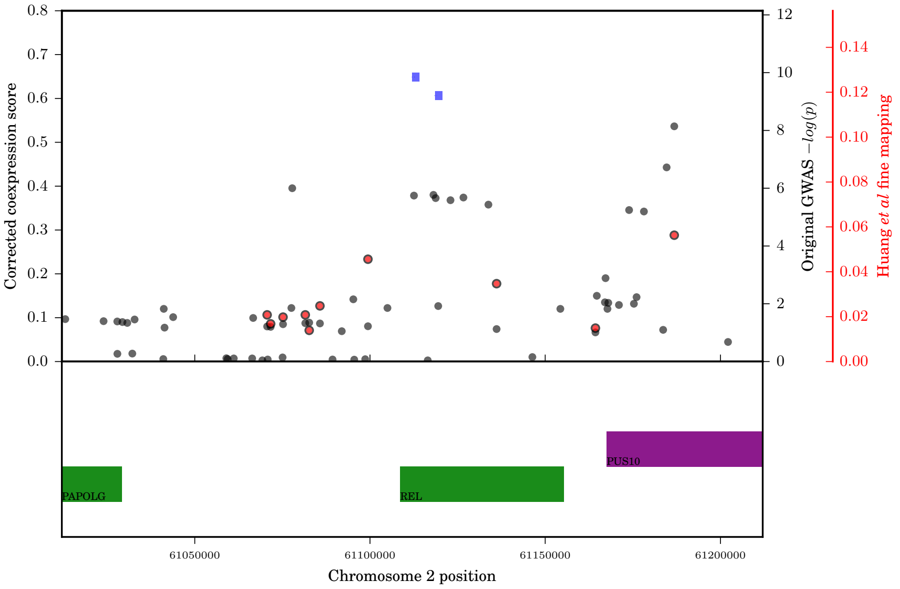

Region surrounding enhancer@chr2:61112067-61112568 [rs6545835] p(Bonferroni)=0.00e+00.

Ulcerative colitis

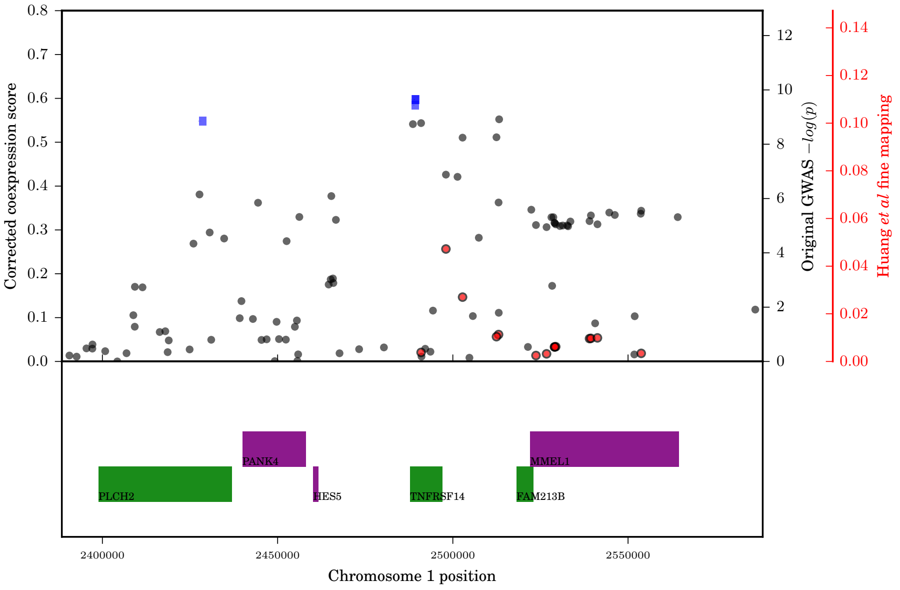

Region surrounding p1@LOC100133445,p1@LOC115110 [rs1886730] p(Bonferroni)=0.00e+00.

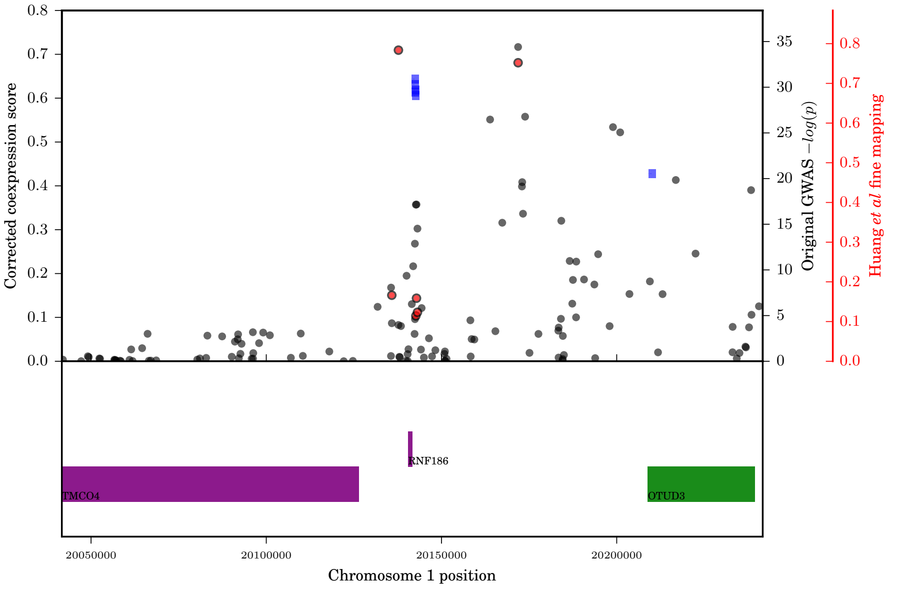

Region surrounding p4@RNF186 [rs12064796] p(Bonferroni)=0.00e+00.

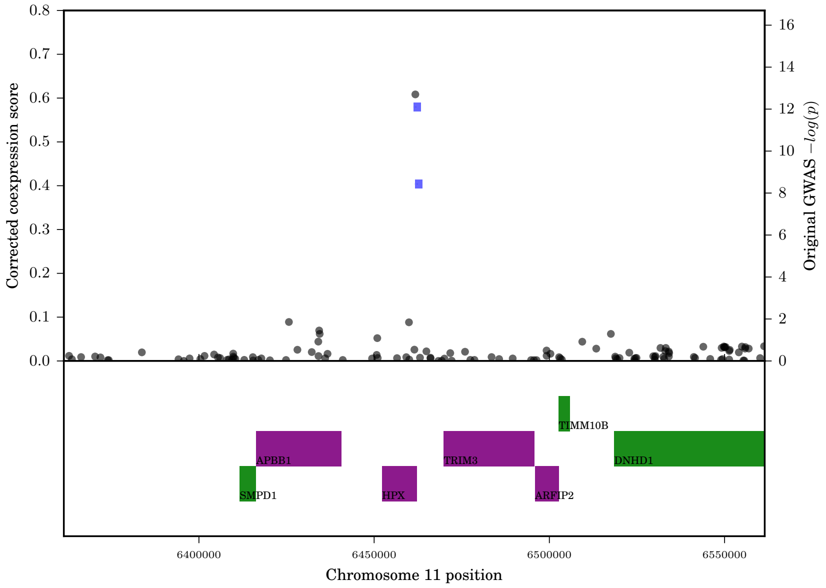

Region surrounding p@chr11:6461452..6461464,- [rs10839564] p(Bonferroni)=0.00e+00.

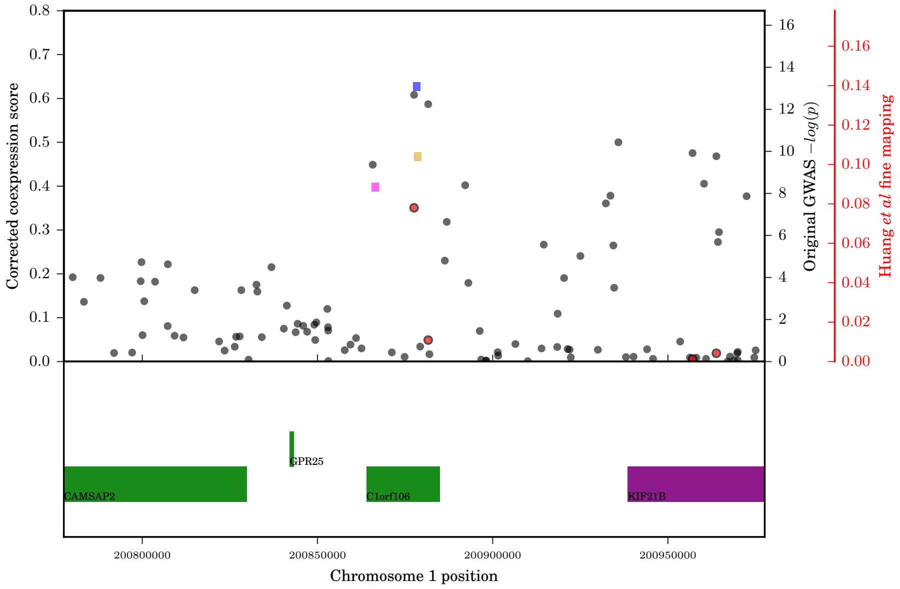

Region surrounding p11@C1orf106 [rs7554511] p(Bonferroni)=0.00e+00.

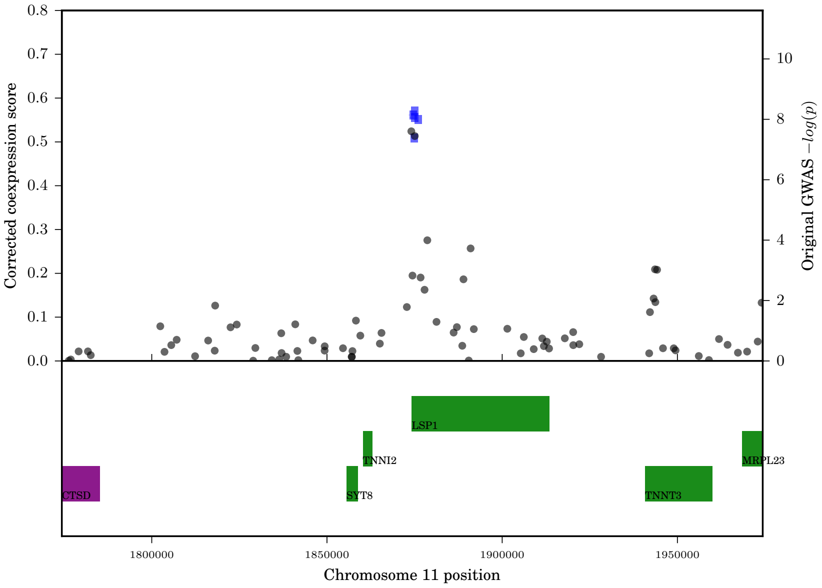

Region surrounding p2@LSP1 [rs907611] p(Bonferroni)=0.00e+00.

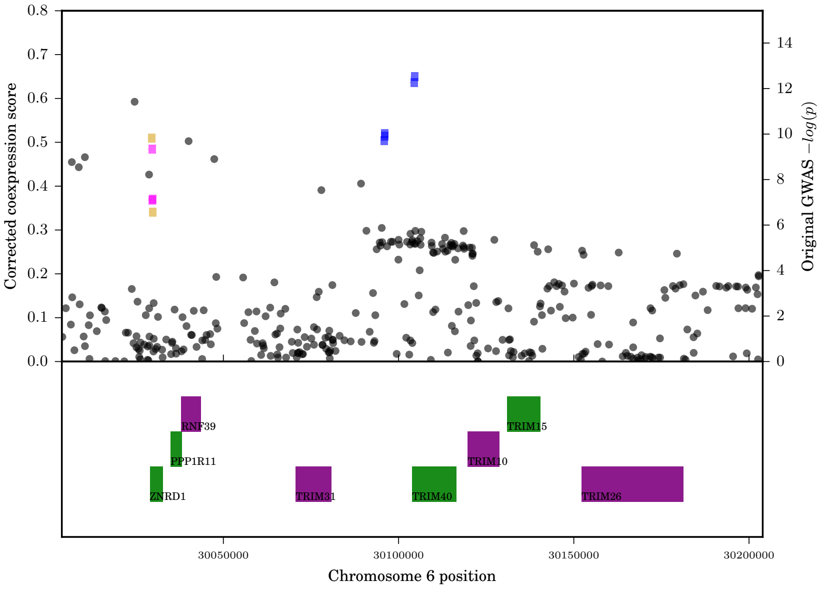

Region surrounding p1@TRIM40 [rs9261467] p(Bonferroni)=0.00e+00.

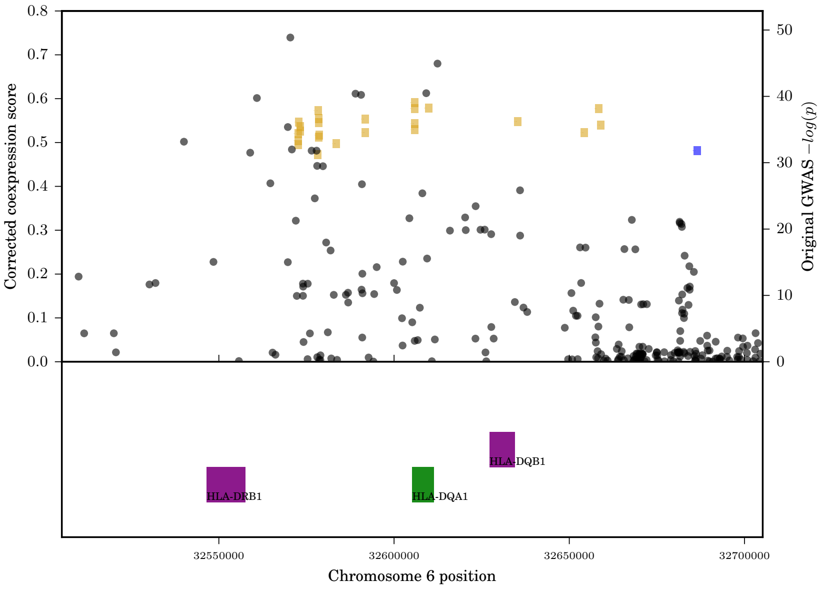

Region surrounding p2@HLA-DQA1 [rs9272426] p(Bonferroni)=0.00e+00.

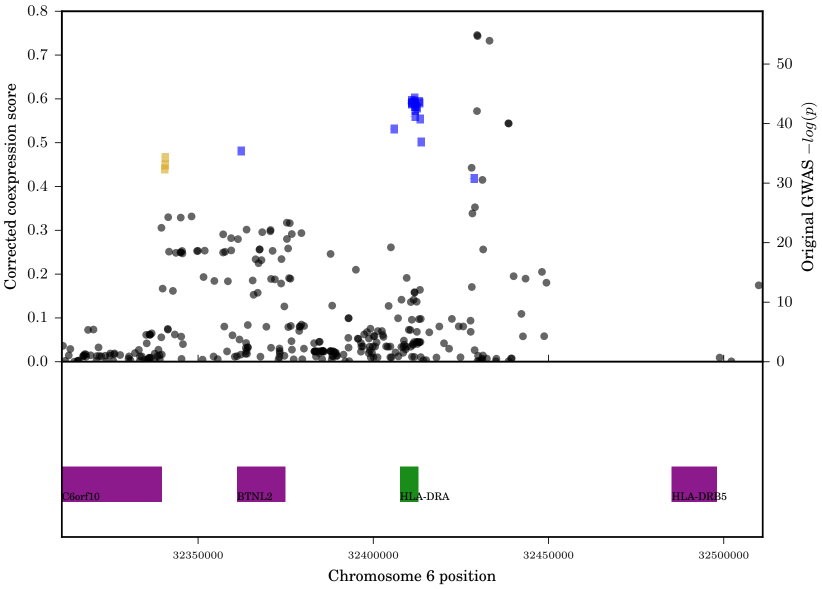

Region surrounding p3@HLA-DQA1 [rs3135391] p(Bonferroni)=0.00e+00.

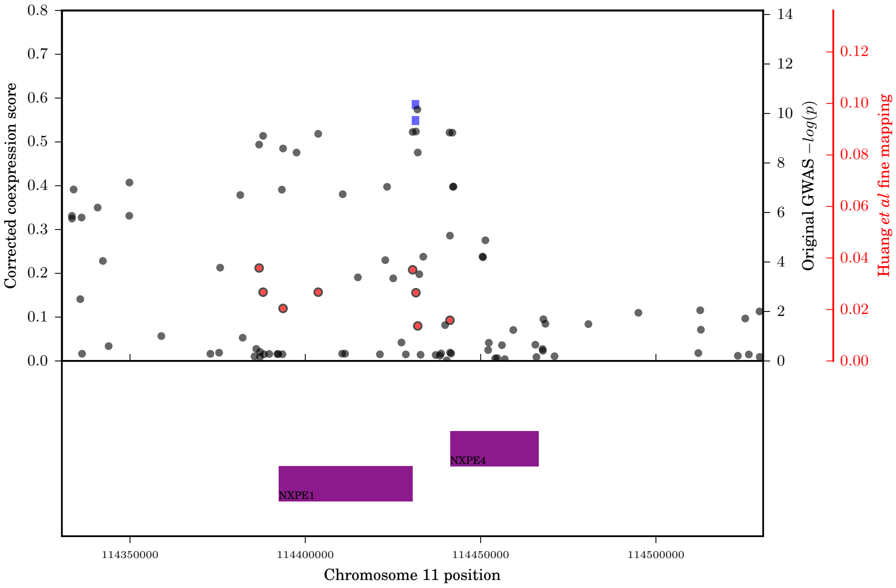

Region surrounding p2@FAM55A [rs661946] p(Bonferroni)=0.00e+00.

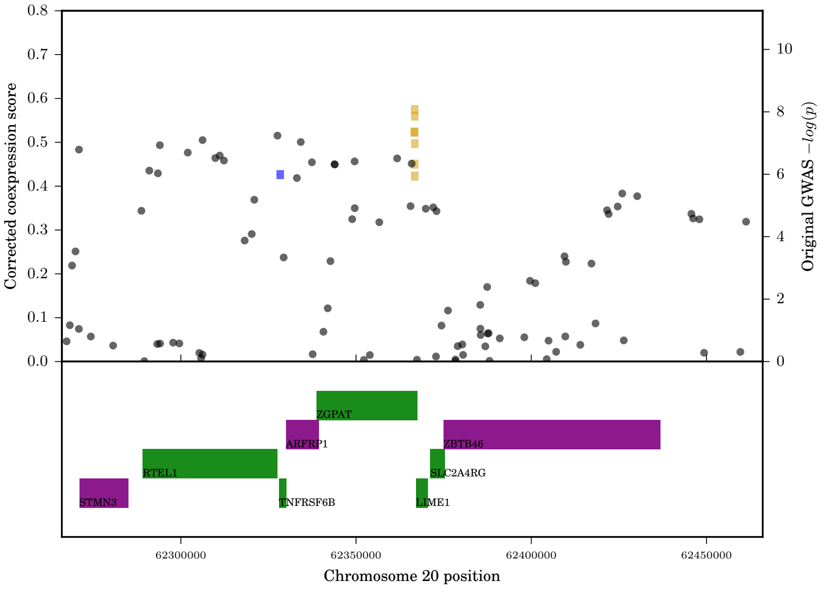

Region surrounding p@chr20:62366043..62366057,+ [rs2427533] p(Bonferroni)=0.00e+00.

LDL cholesterol

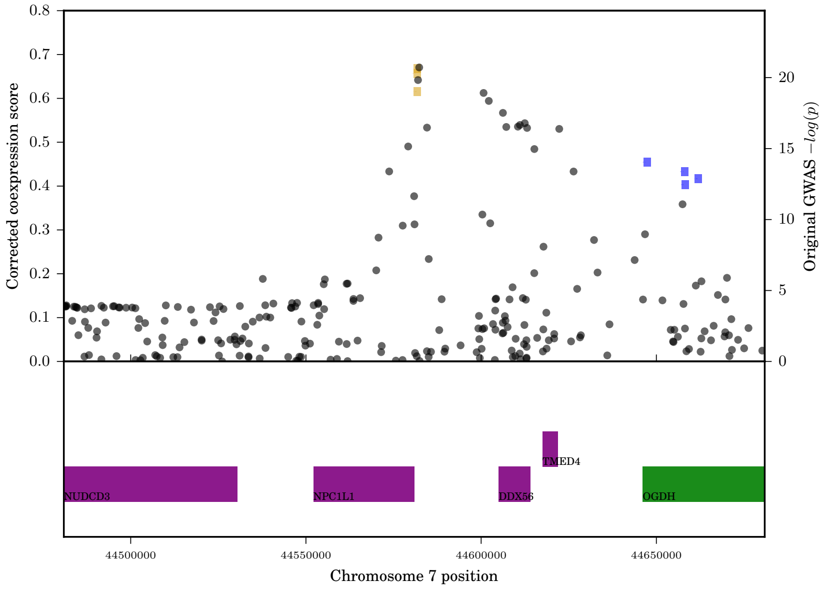

Region surrounding p2@NPC1L1 [rs41279633—rs17655652] p(Bonferroni)=1.69e-02.

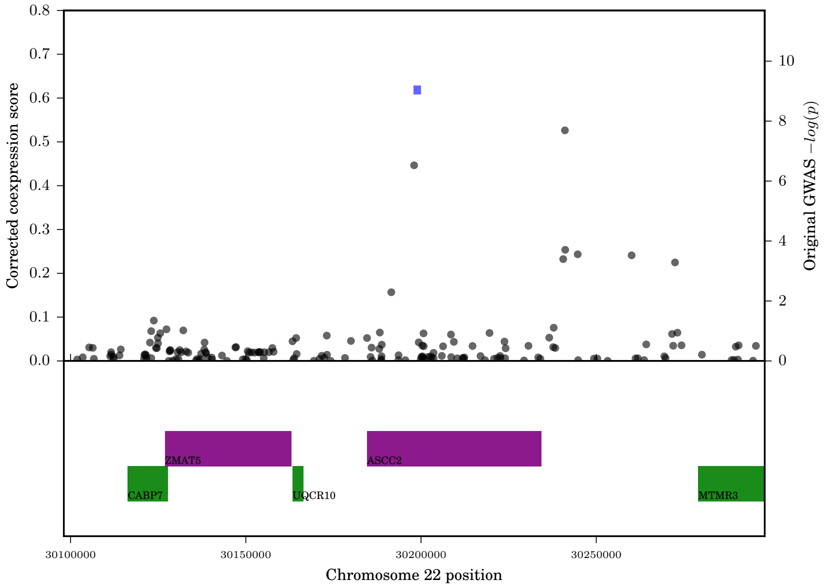

Region surrounding p2@ASCC2 [rs4823054] p(Bonferroni)=0.00e+00.

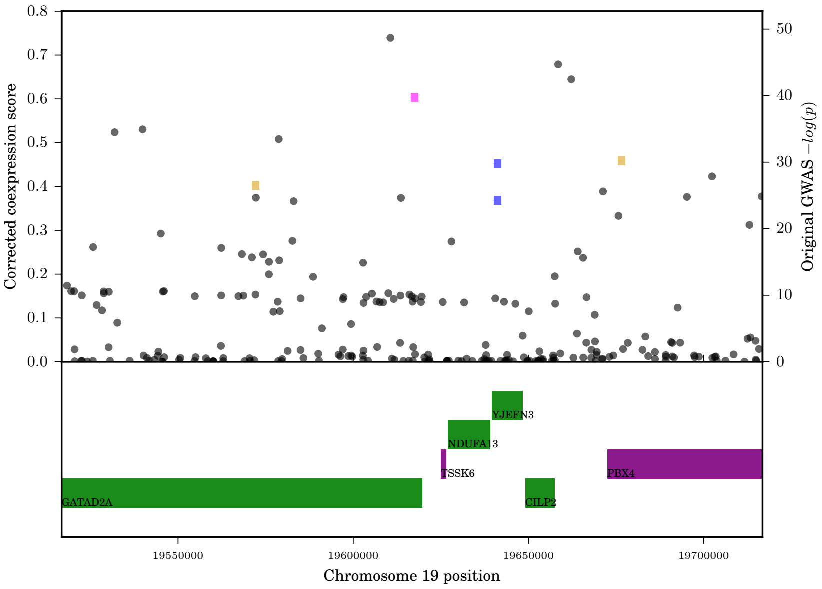

Region surrounding p@chr19:19616795..19616800,+ [rs1063966] p(Bonferroni)=0.00e+00.

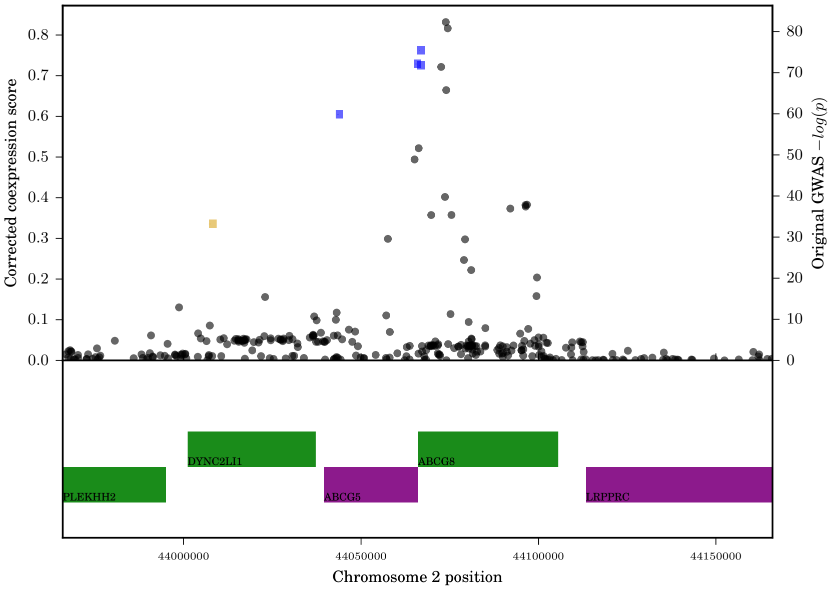

Region surrounding p3@ABCG5 [rs11887534] p(Bonferroni)=0.00e+00.

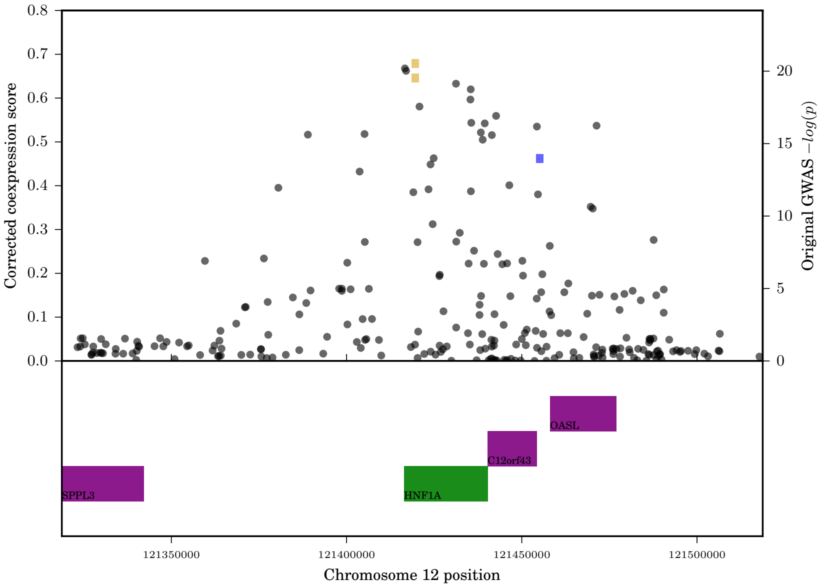

Region surrounding p6@HNF1A-AS1 [rs1169286] p(Bonferroni)=0.00e+00.

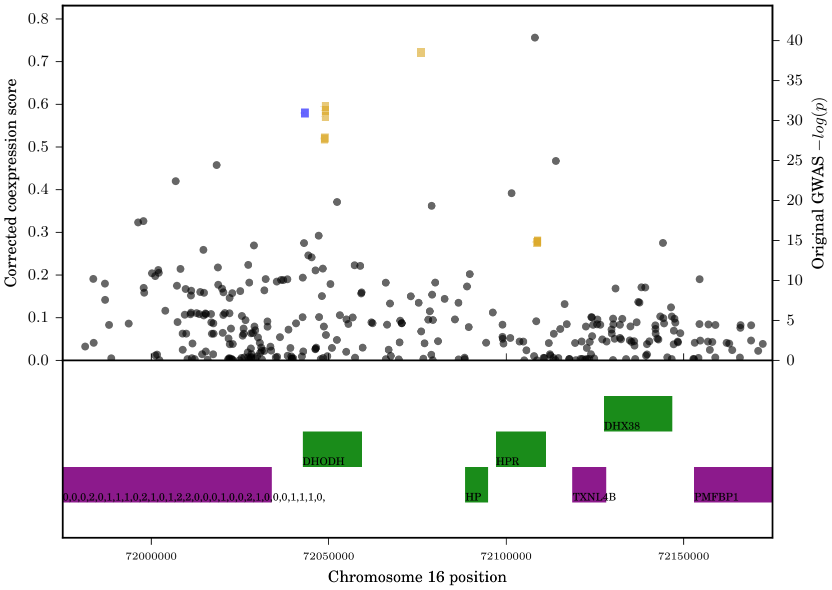

Region surrounding p@chr16:72075048..72075055,- [rs9932951] p(Bonferroni)=1.69e-02.

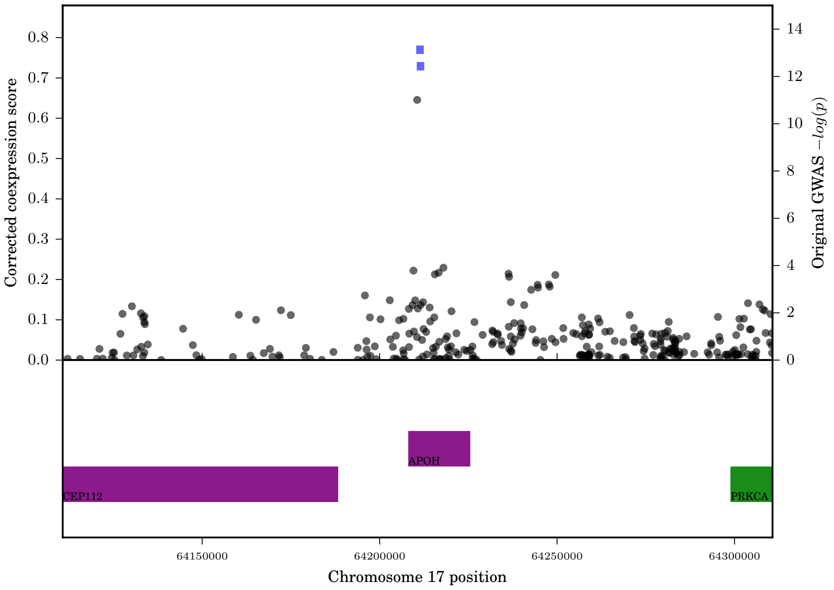

Region surrounding p@chr17:64210704..64210723,+ [rs1801689] p(Bonferroni)=0.00e+00.

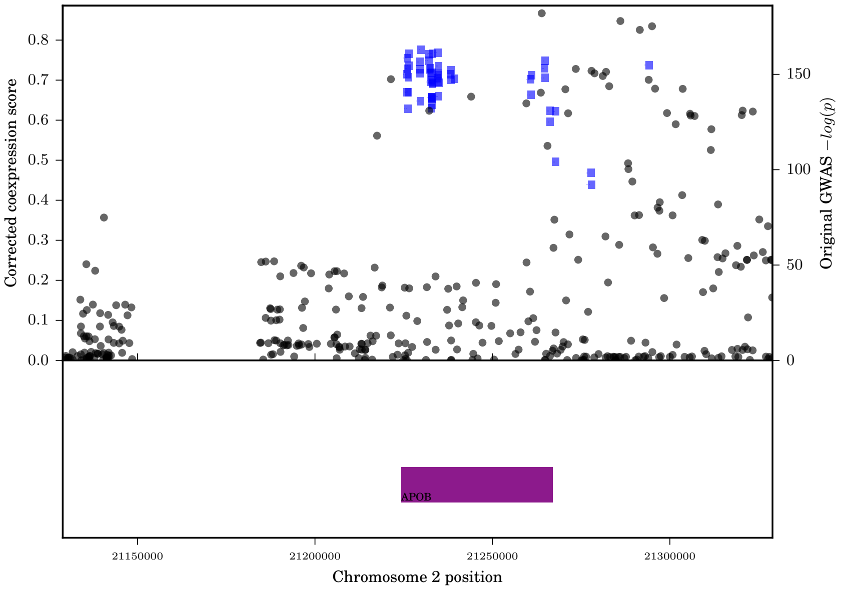

Region surrounding p94@APOB [rs1801701] p(Bonferroni)=0.00e+00.

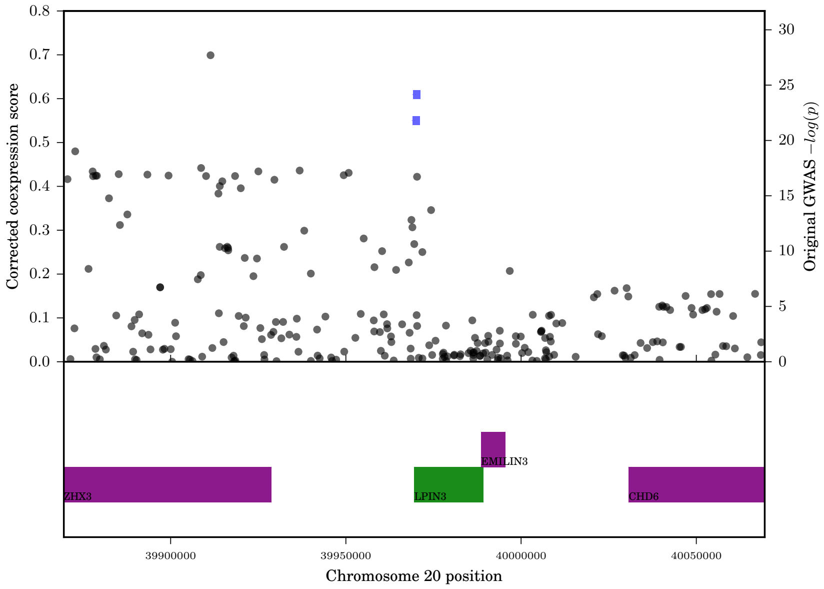

Region surrounding p2@LPIN3 [rs6029636] p(Bonferroni)=1.69e-02.

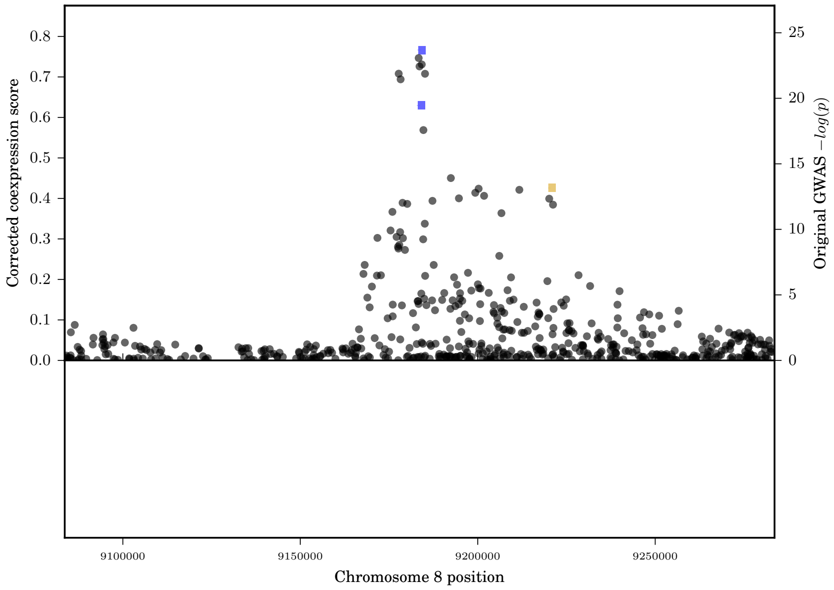

Region surrounding p2@ENST00000518619 [rs9987289—rs4841132] p(Bonferroni)=0.00e+00.

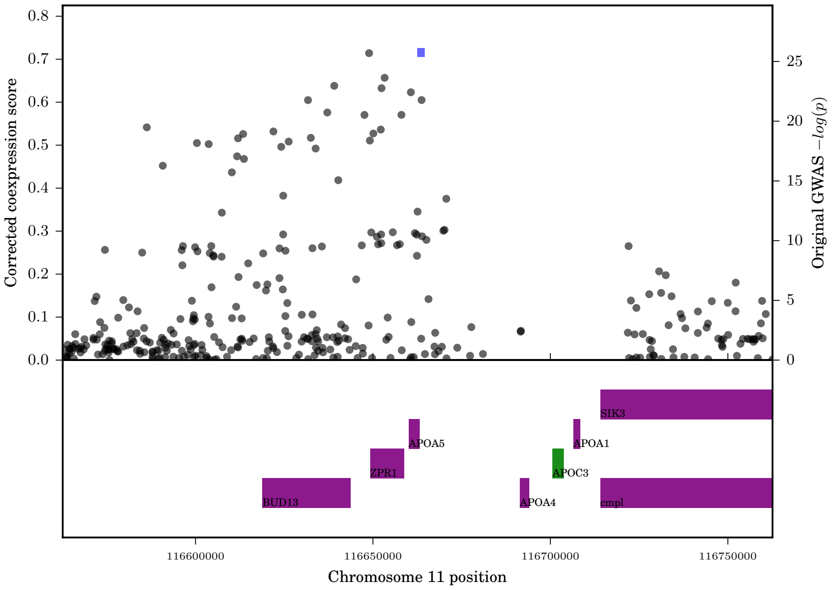

Region surrounding p1@APOA5 [rs651821] p(Bonferroni)=0.00e+00.

HDL cholesterol

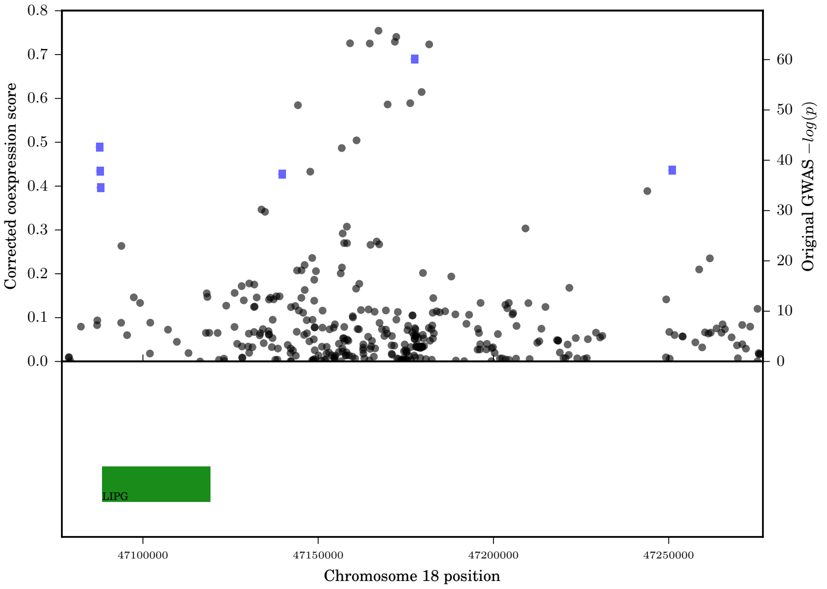

Region surrounding p@chr18:47176875..47176889,+ [rs4939886—rs4939887—rs4939888] p(Bonferroni)=0.00e+00.

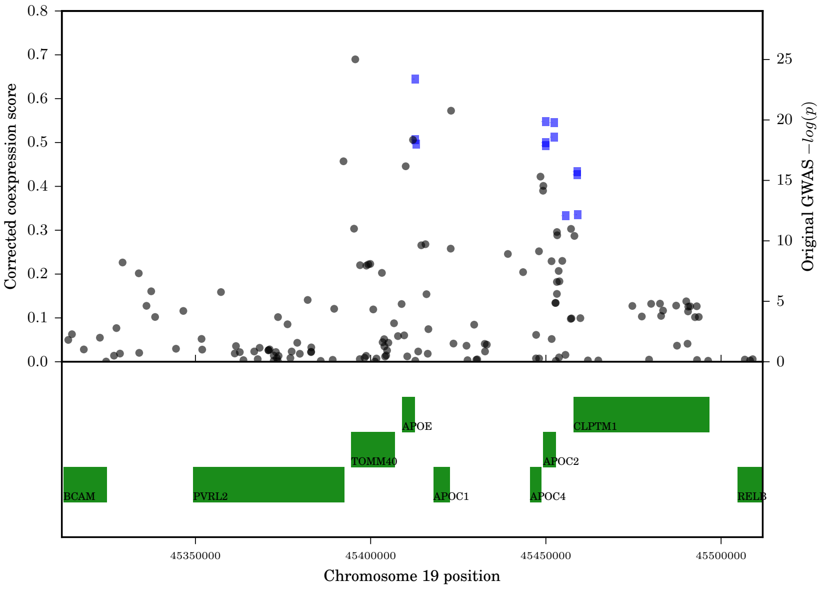

Region surrounding p@chr19:45411878..45411884,- [rs7412] p(Bonferroni)=1.58e-02.

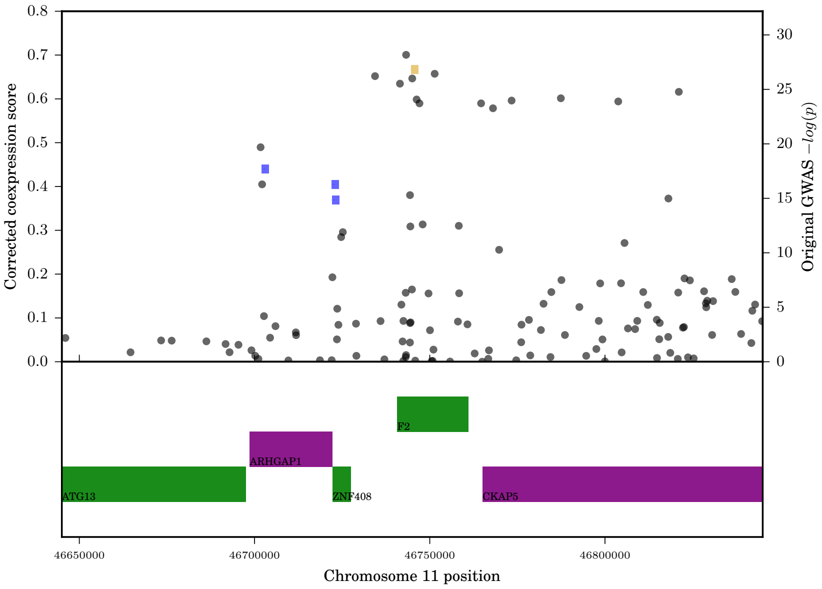

Region surrounding p2@F2 [rs2070852—rs5896] p(Bonferroni)=1.58e-02.

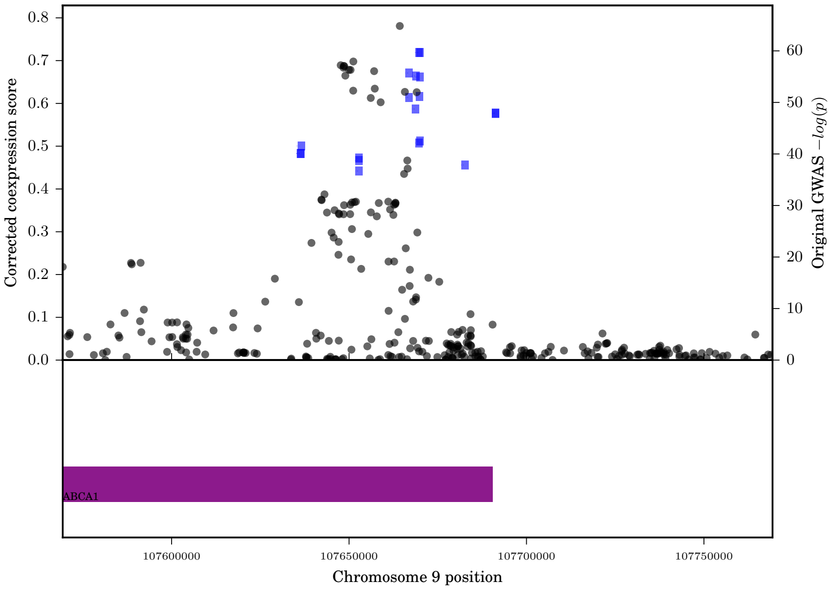

Region surrounding p@chr9:107669326..107669336,+ [rs4100654—rs13284054] p(Bonferroni)=0.00e+00.

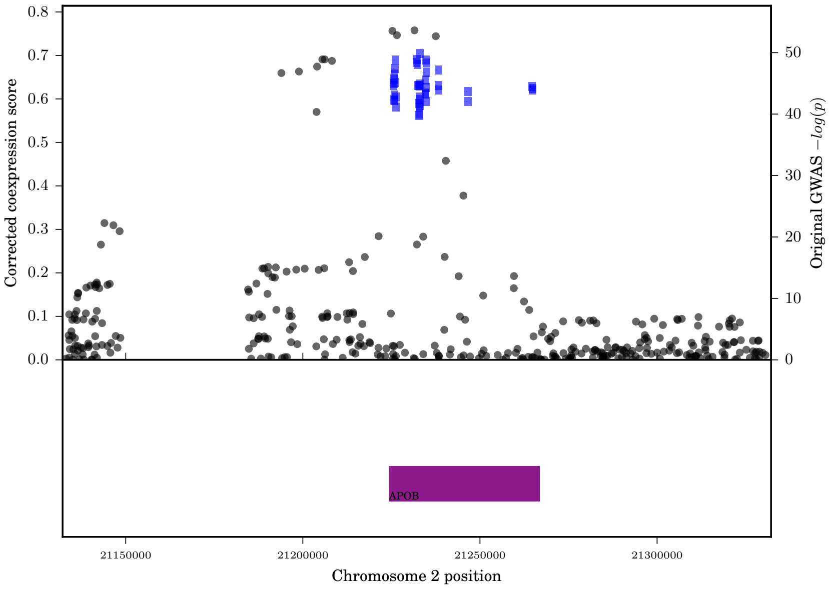

Region surrounding p13@BC172787 [rs693] p(Bonferroni)=0.00e+00.

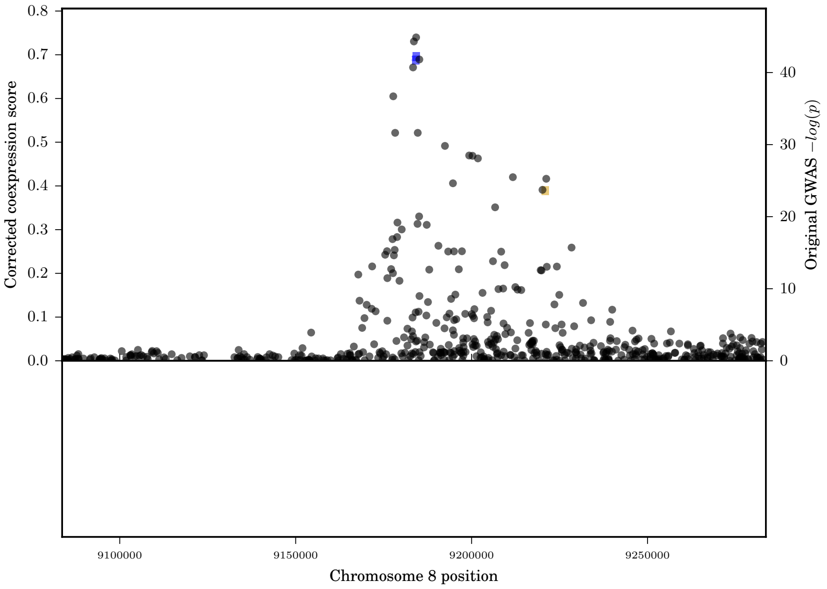

Region surrounding p2@ENST00000518619 [rs9987289—rs4841132] p(Bonferroni)=0.00e+00.

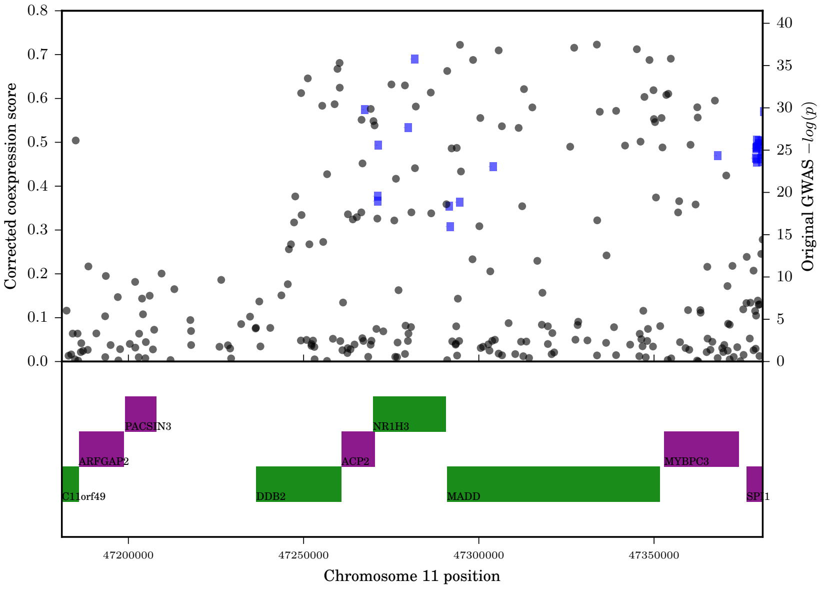

Region surrounding chr11:47281010..47281020,+ [rs11039155] p(Bonferroni)=0.00e+00.

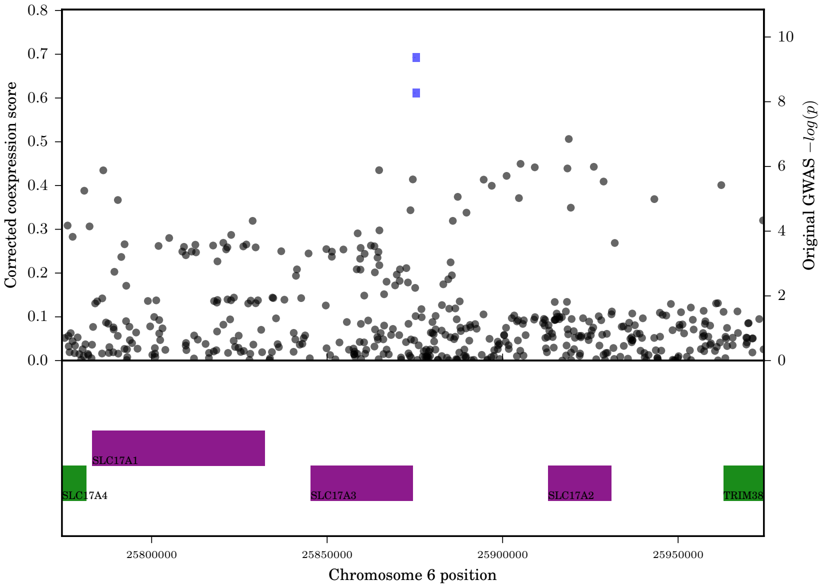

Region surrounding p1@SLC17A3 [rs13198474] p(Bonferroni)=0.00e+00.

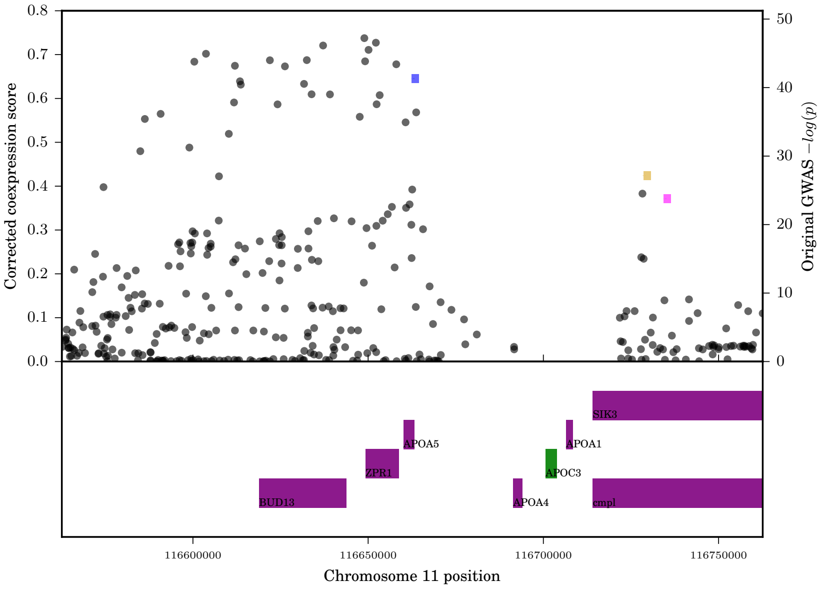

Region surrounding p1@APOA5 [rs651821] p(Bonferroni)=3.16e-02.

Total cholesterol

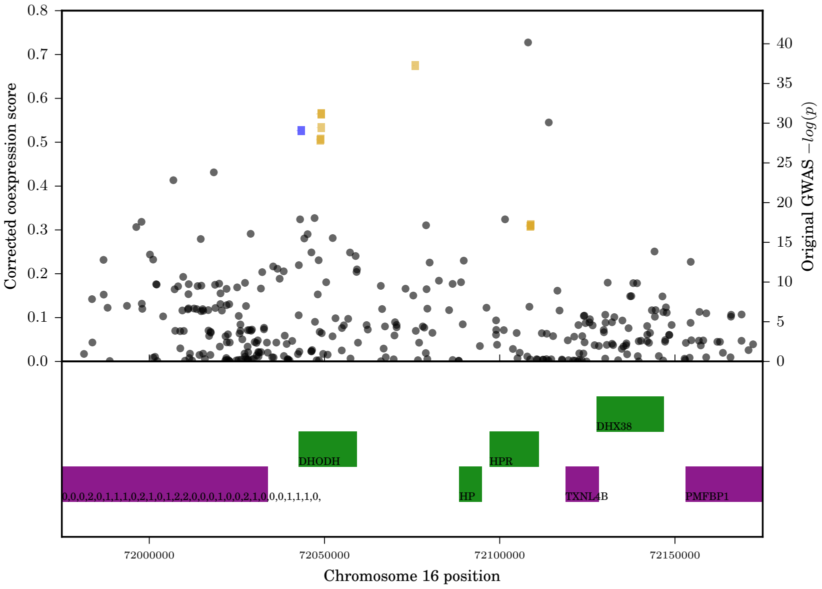

Region surrounding p@chr16:72075048..72075055,- [rs9932951] p(Bonferroni)=1.68e-02.

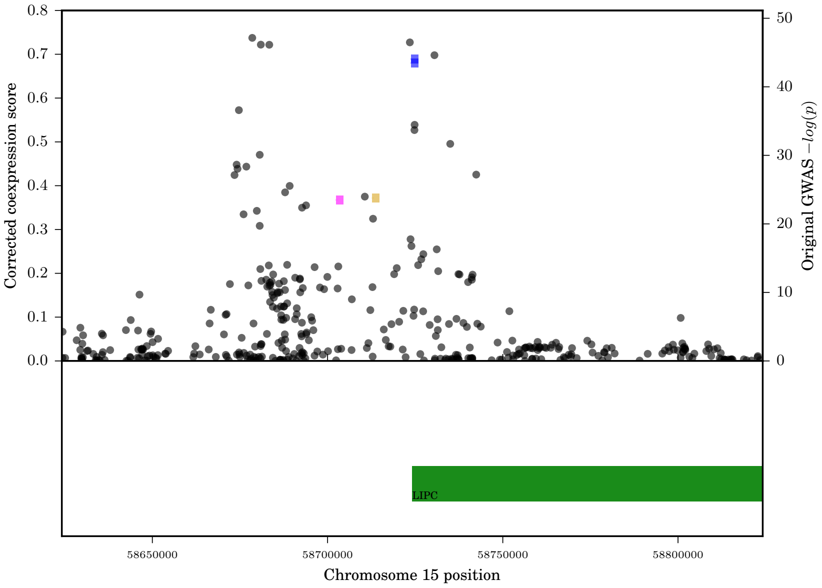

Region surrounding p3@LIPC [rs2070895] p(Bonferroni)=0.00e+00.

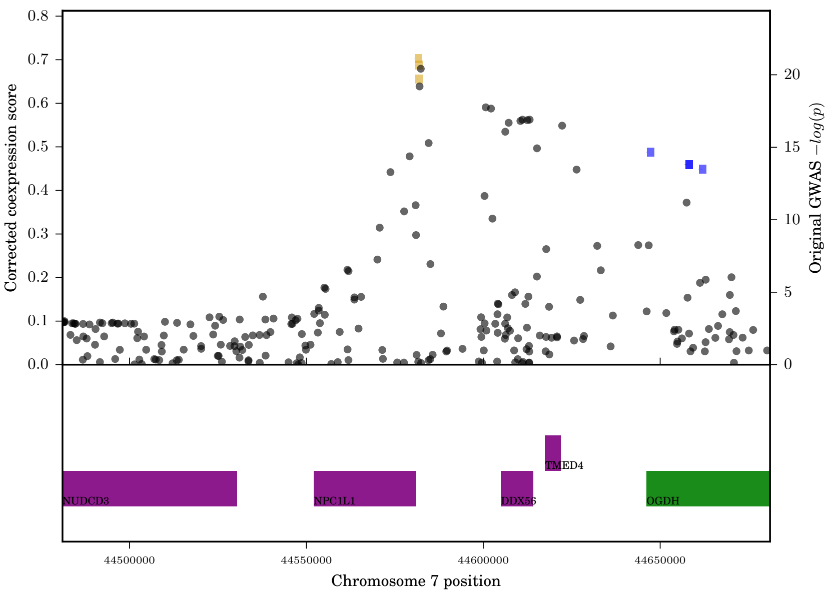

Region surrounding p@chr7:44581054..44581057,+ [rs41279633—rs17655652] p(Bonferroni)=0.00e+00.

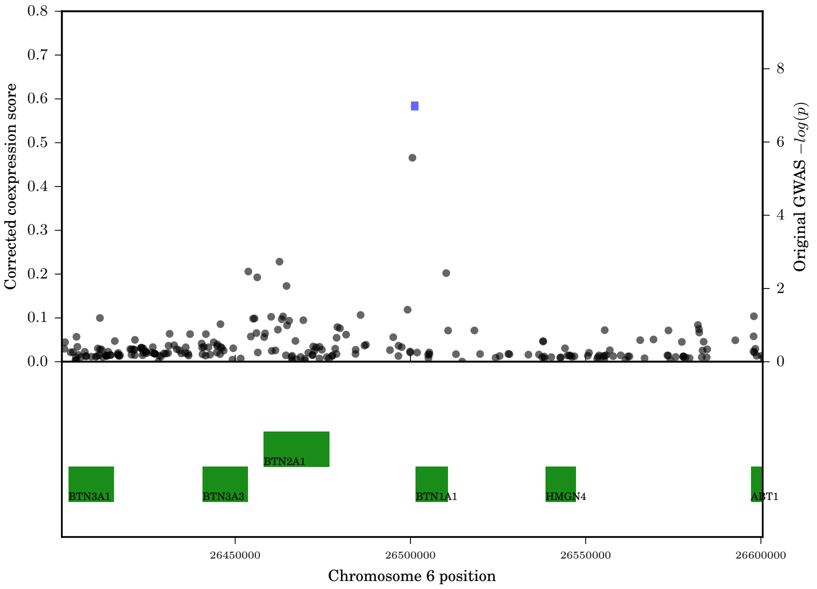

Region surrounding p@chr6:26500524..26500540,+ [rs13194984] p(Bonferroni)=1.68e-02.

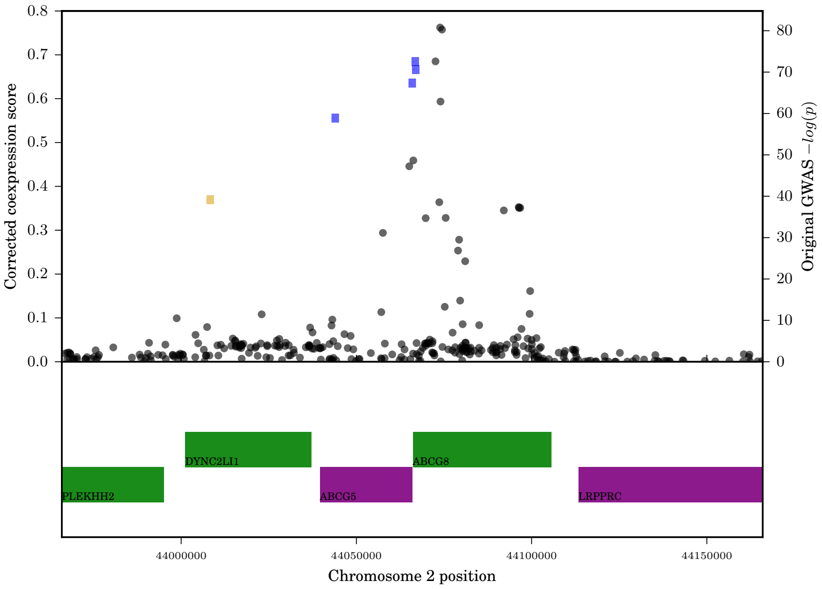

Region surrounding p3@ABCG5 [rs11887534] p(Bonferroni)=0.00e+00.

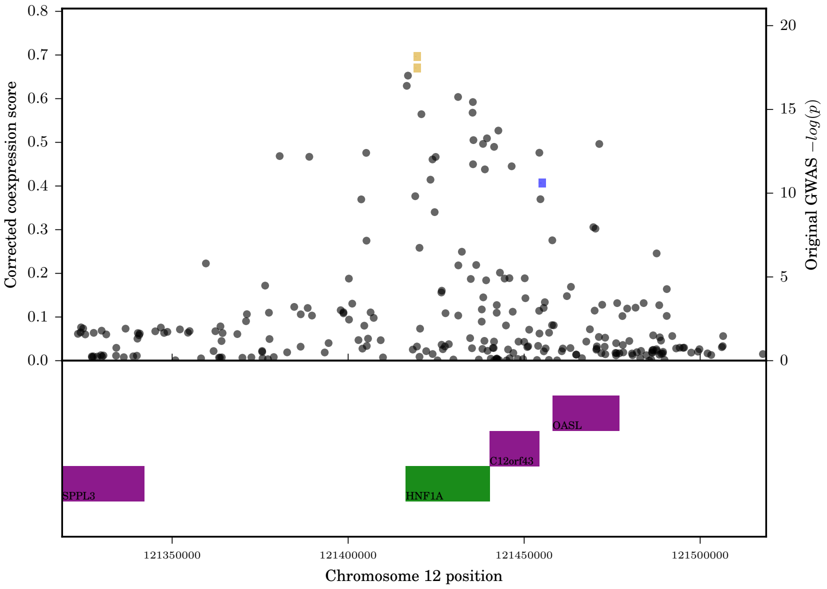

Region surrounding p6@HNF1A-AS1 [rs1169286] p(Bonferroni)=0.00e+00.

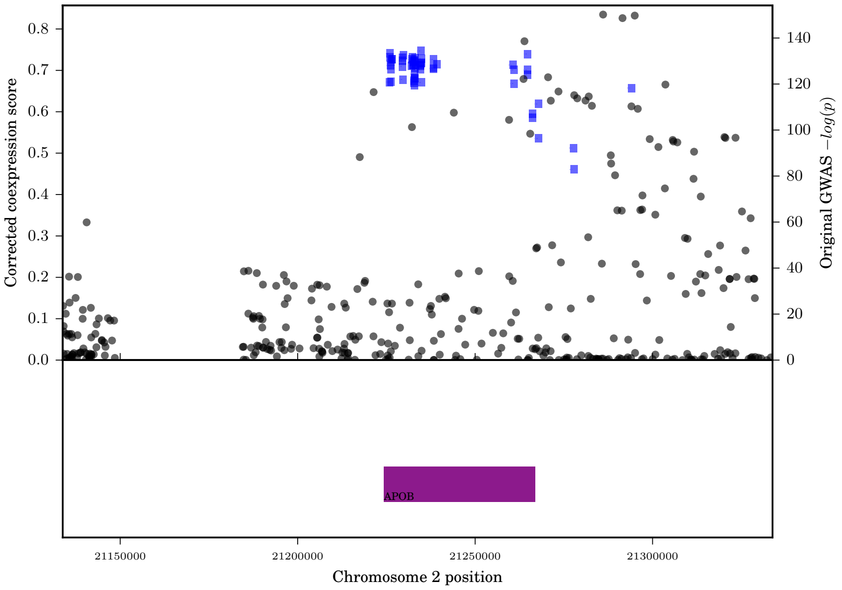

Region surrounding p58@APOB [rs533617] p(Bonferroni)=0.00e+00.

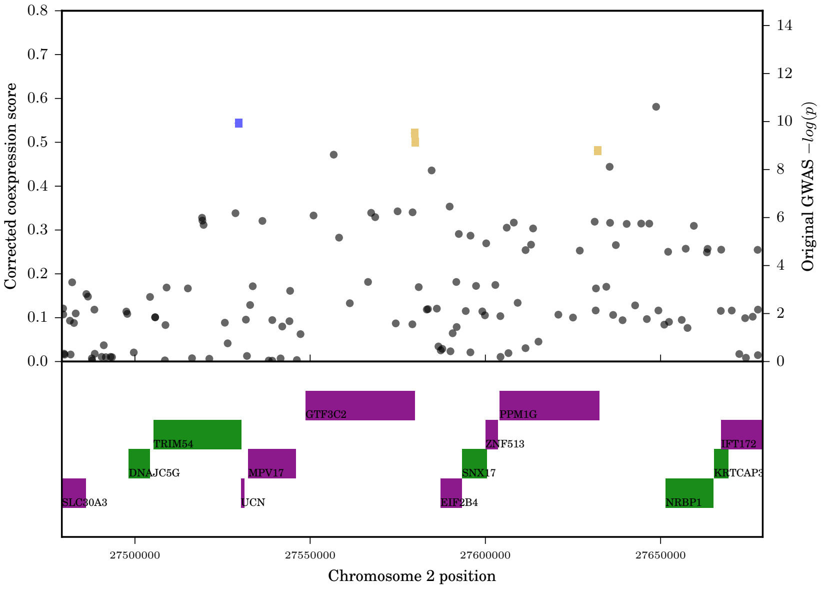

Region surrounding p1@ENST00000447070 [rs6760828] p(Bonferroni)=0.00e+00.

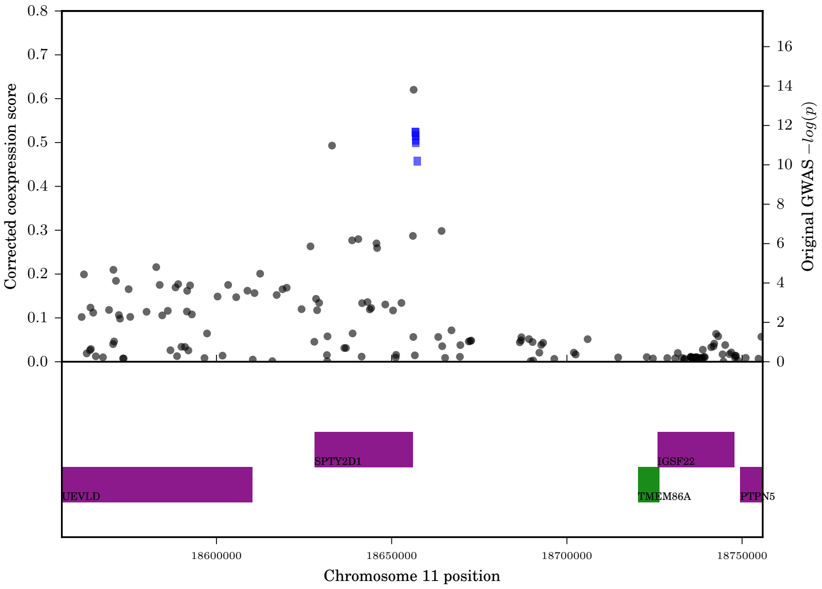

Region surrounding p1@SPTY2D1 [rs7943121] p(Bonferroni)=1.68e-02.

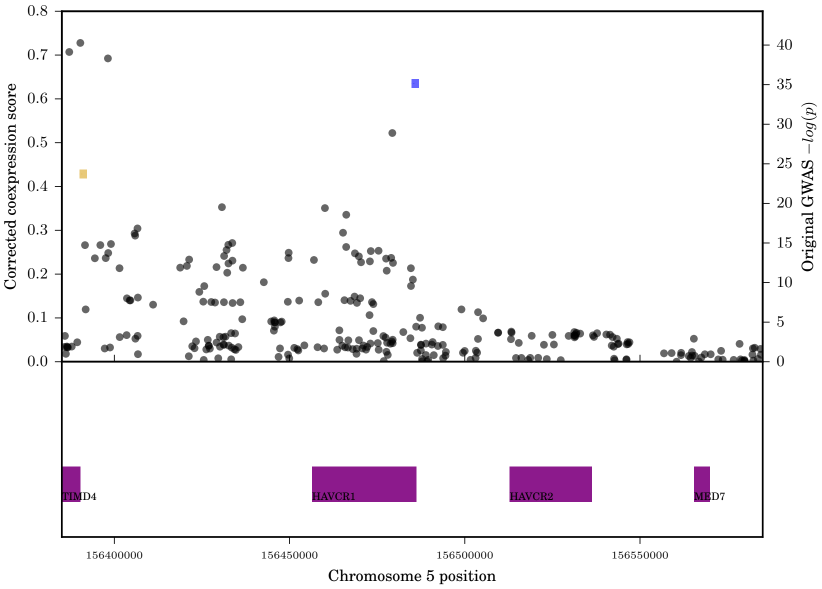

Region surrounding p3@HAVCR1 [rs67960962] p(Bonferroni)=0.00e+00.

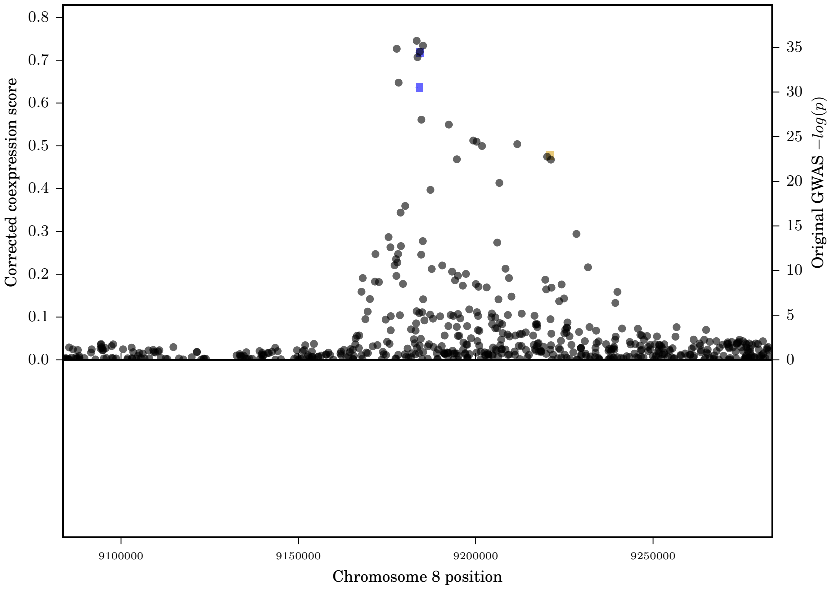

Region surrounding p2@ENST00000518619 [rs11774381—rs9987289—rs4841132] p(Bonferroni)=0.00e+00.

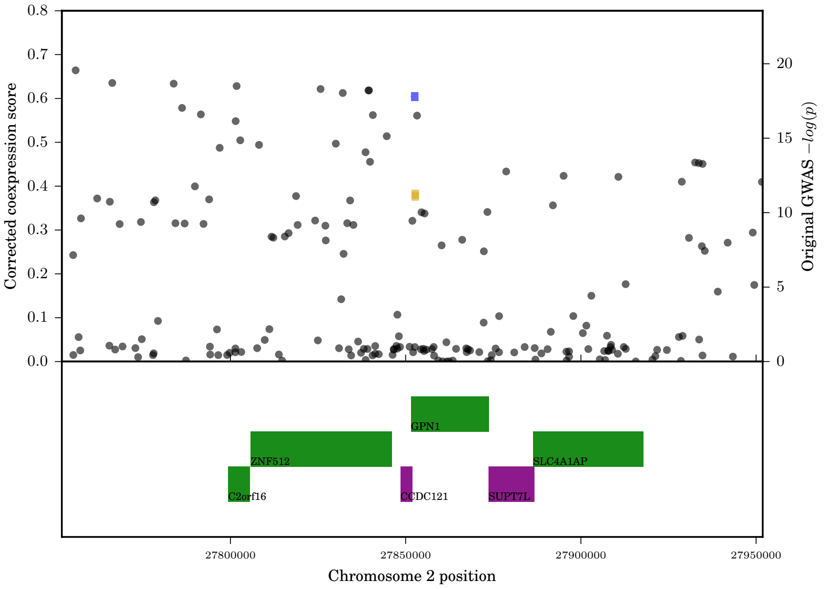

Region surrounding p1@GPN1 [rs3749147] p(Bonferroni)=0.00e+00.

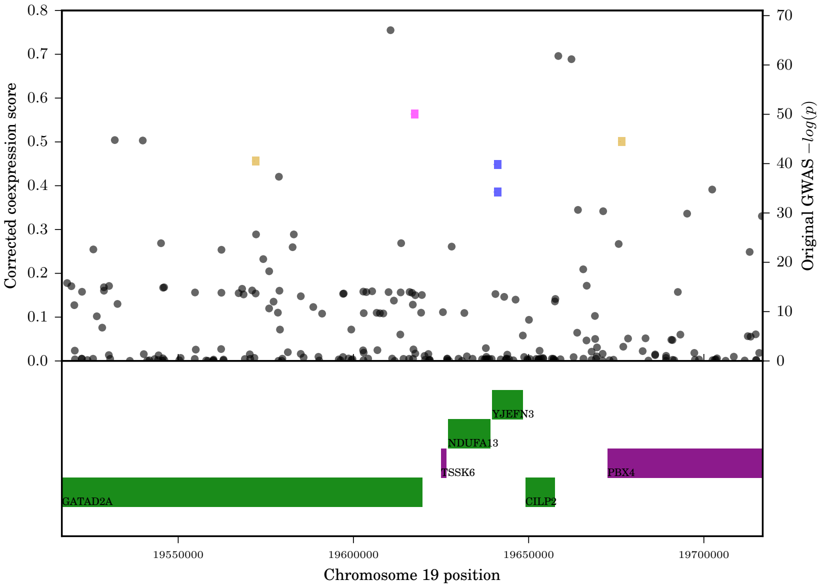

Region surrounding p@chr19:19616795..19616800,+ [rs1063966] p(Bonferroni)=1.68e-02.

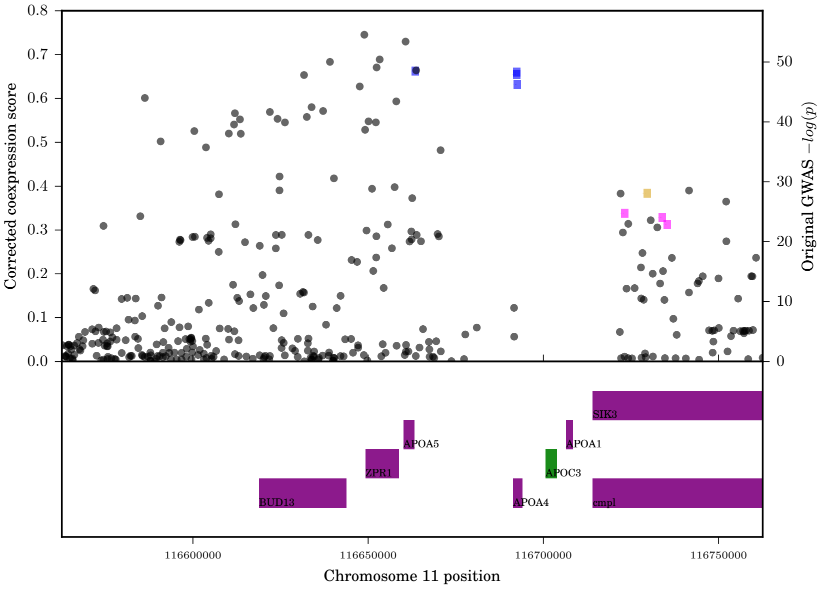

Region surrounding p1@APOA5 [rs651821] p(Bonferroni)=0.00e+00.

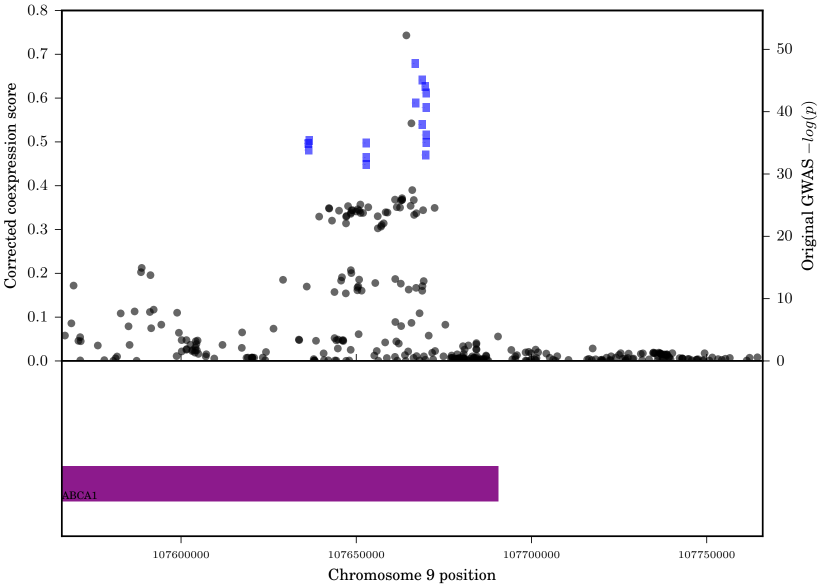

Region surrounding p5@ABCA1 [rs1800978] p(Bonferroni)=0.00e+00.

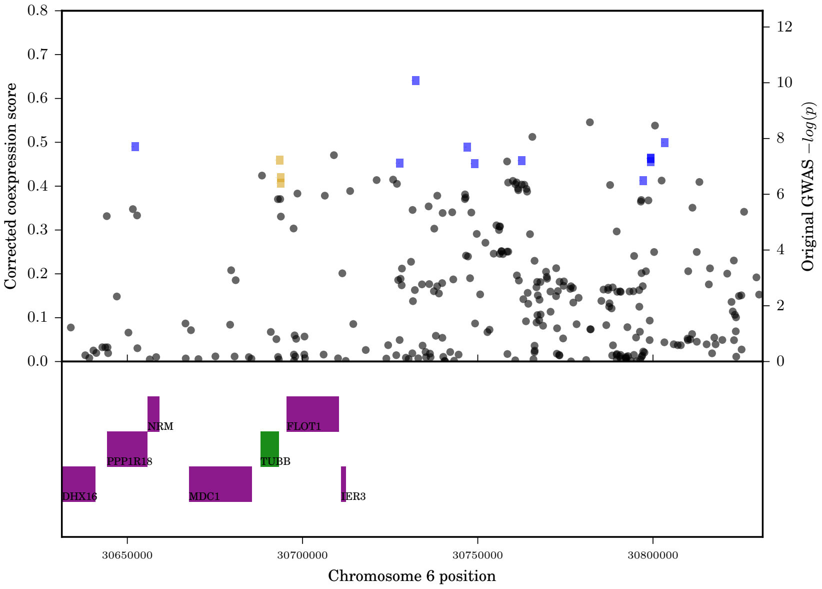

Region surrounding enhancer@chr6:30731311-30731508 [rs3095338] p(Bonferroni)=0.00e+00.

Triglycerides

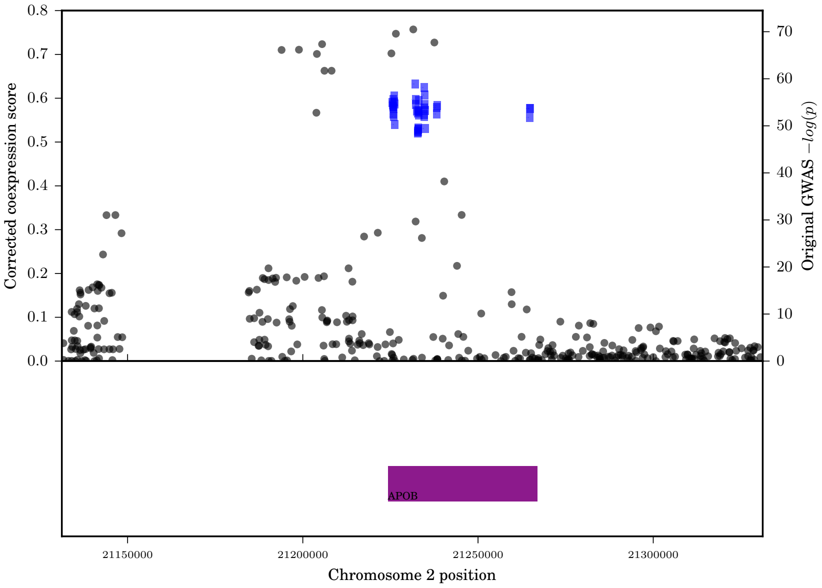

Region surrounding p67@APOB [rs676210] p(Bonferroni)=0.00e+00.

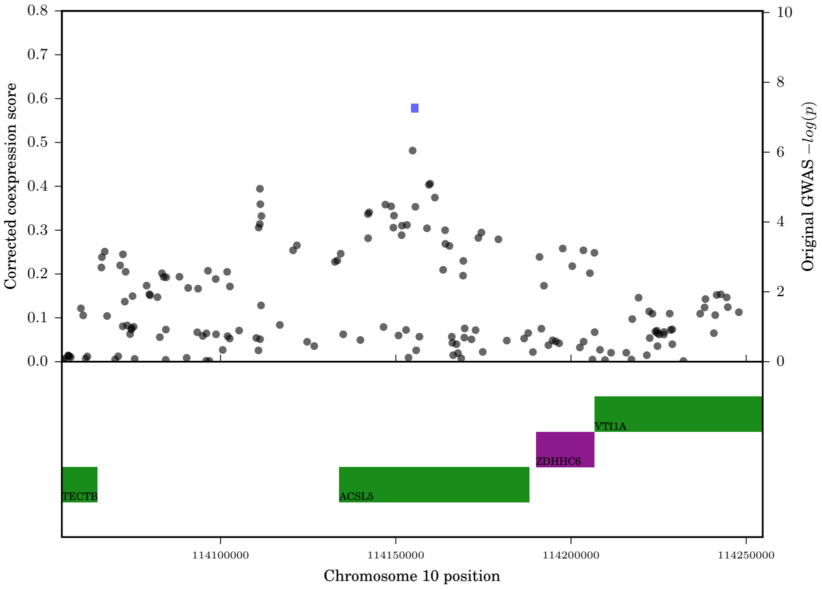

Region surrounding p32@ACSL5 [rs11195943] p(Bonferroni)=3.62e-02.

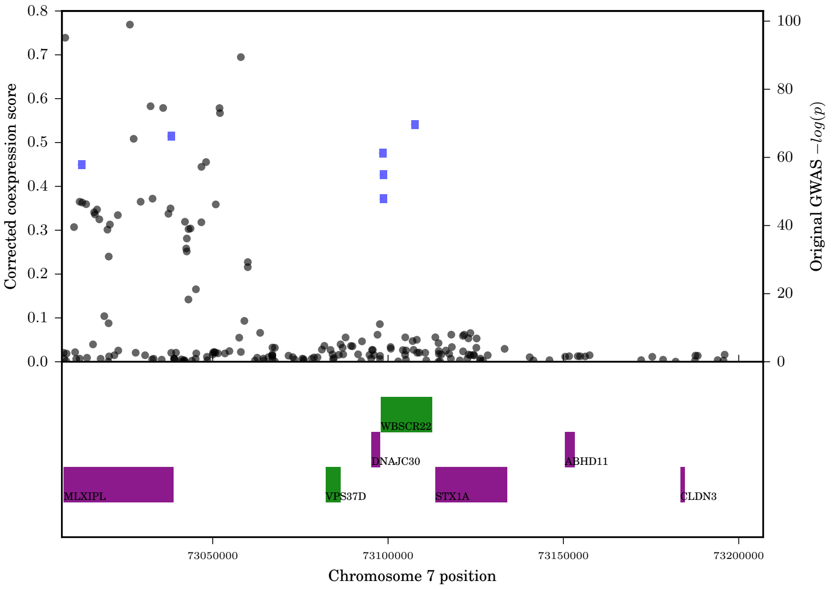

Region surrounding p@chr7:73106975..73106980,+ [rs2293490] p(Bonferroni)=3.62e-02.

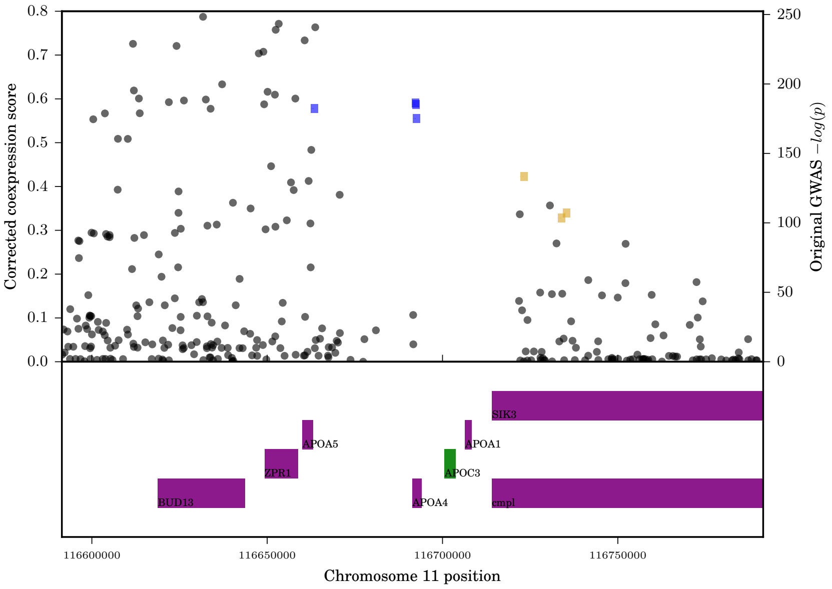

Region surrounding p@chr11:116691448..116691464,- [rs5110—rs675] p(Bonferroni)=3.62e-02.

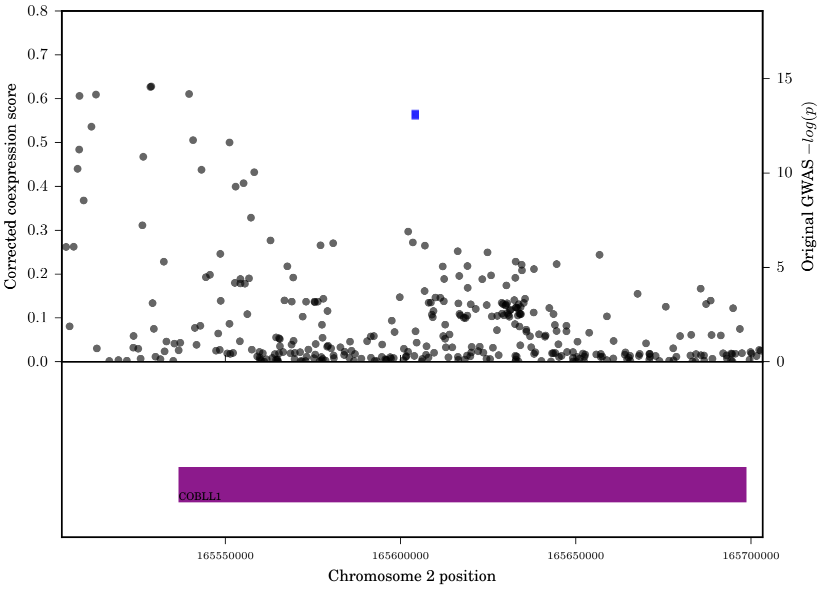

Region surrounding p@chr2:165603346..165603357,- [rs10178921] p(Bonferroni)=1.81e-02.

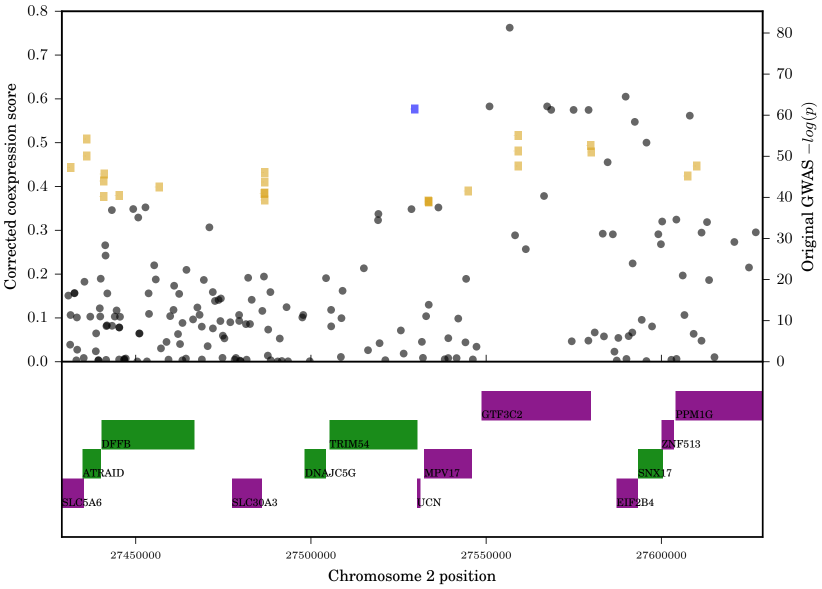

Region surrounding p@chr2:27528919..27528940,+ [rs4665963] p(Bonferroni)=3.62e-02.

Height

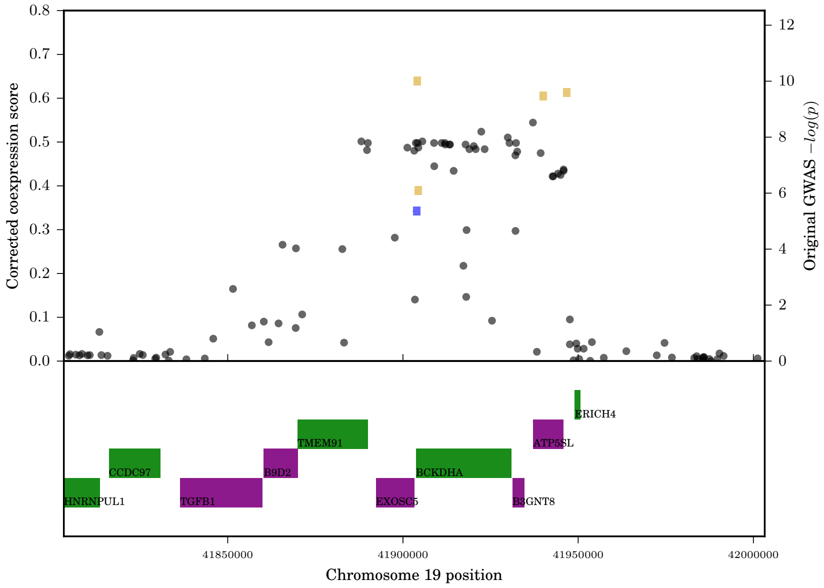

Region surrounding p1@EXOSC5 [rs10853751] p(Bonferroni)=0.00e+00.

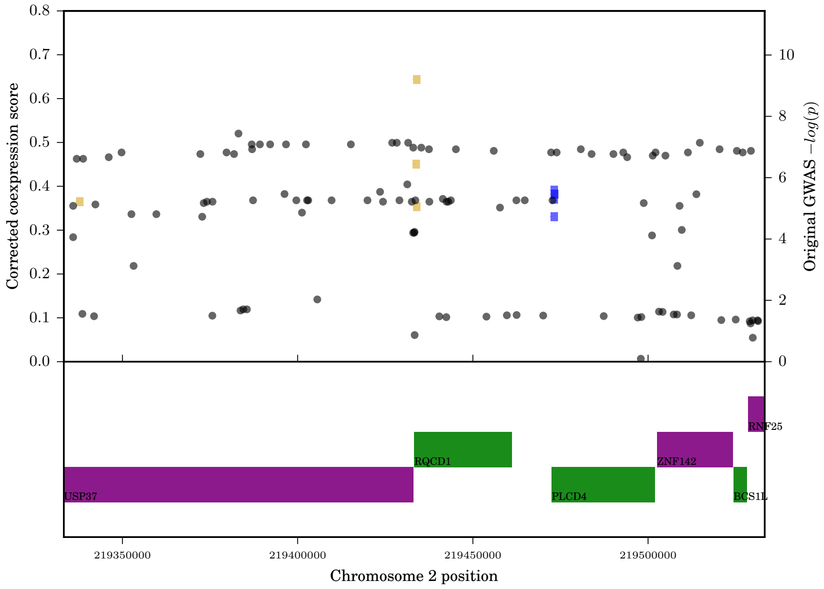

Region surrounding p2@RQCD1 [rs500422] p(Bonferroni)=0.00e+00.

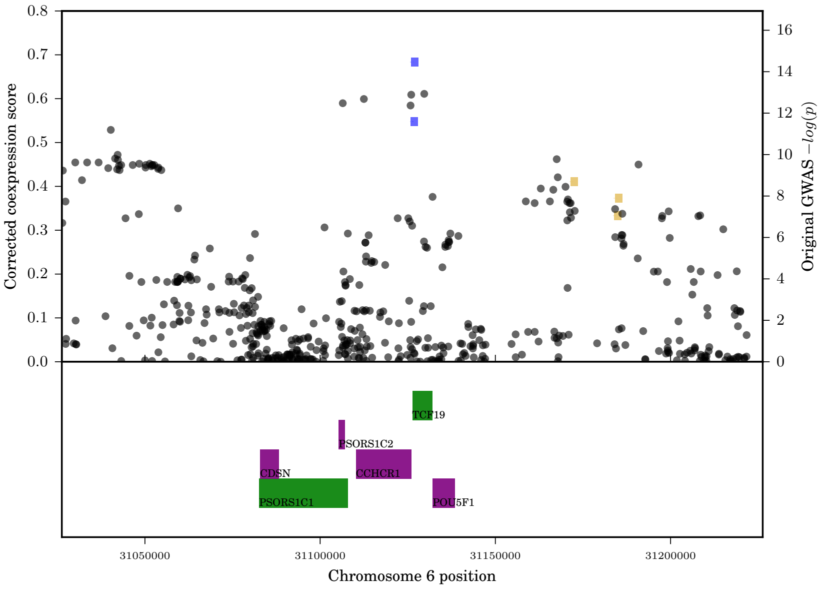

Region surrounding p1@TCF19 [rs720468—rs3130456] p(Bonferroni)=0.00e+00.

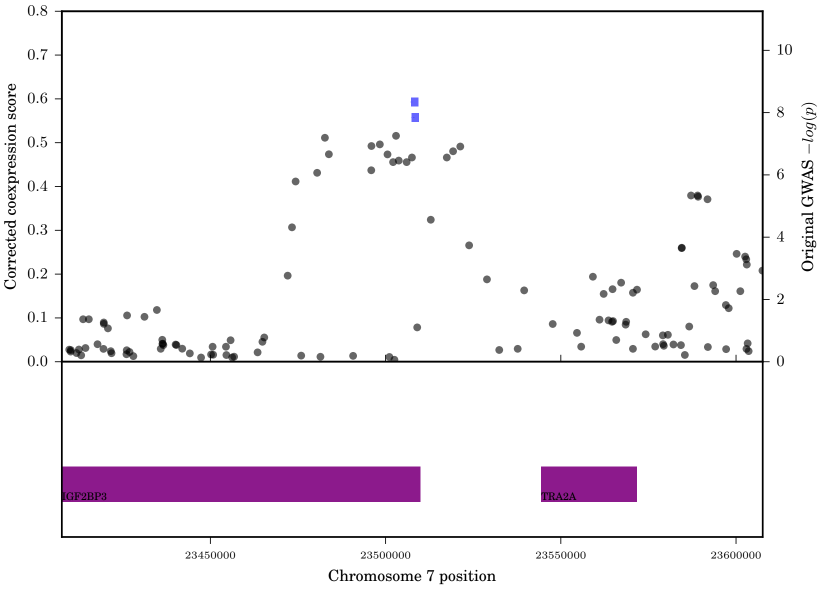

Region surrounding p@chr7:23507613..23507647,+ [rs4496867] p(Bonferroni)=0.00e+00.

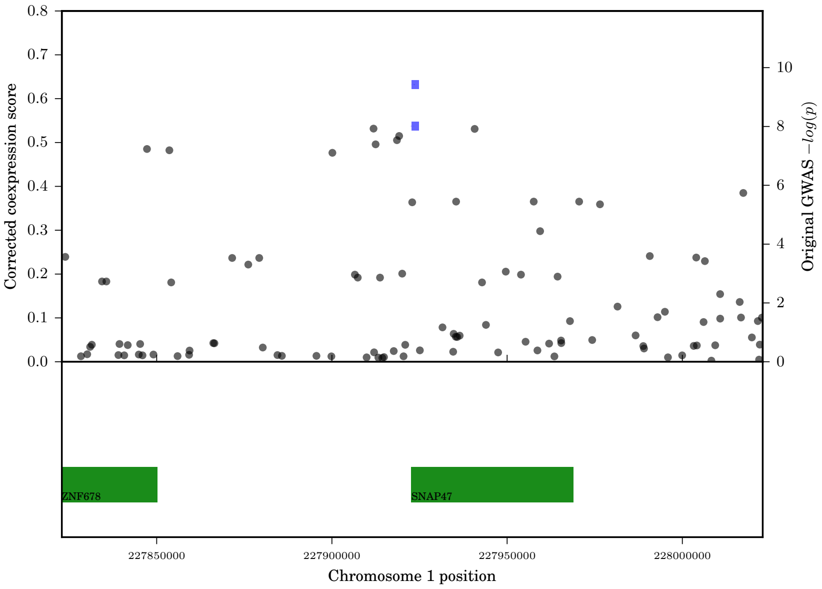

Region surrounding p1@JMJD4 [rs2295994] p(Bonferroni)=0.00e+00.

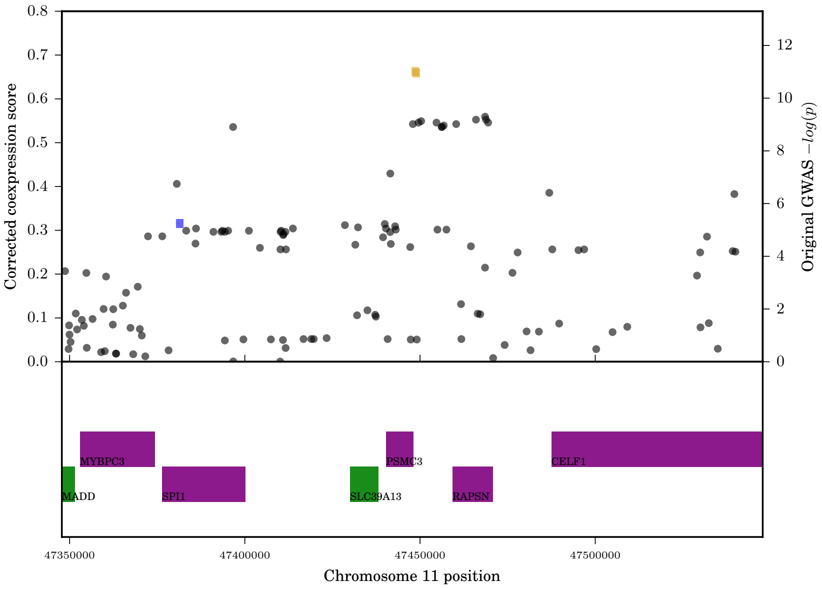

Region surrounding p1@PSMC3 [rs7948705] p(Bonferroni)=0.00e+00.

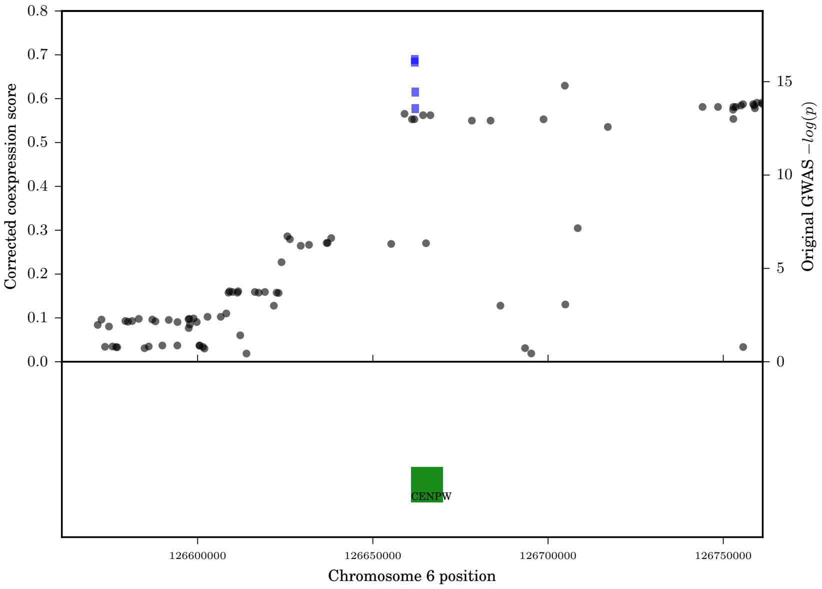

Region surrounding p1@CENPW [rs9388486] p(Bonferroni)=0.00e+00.

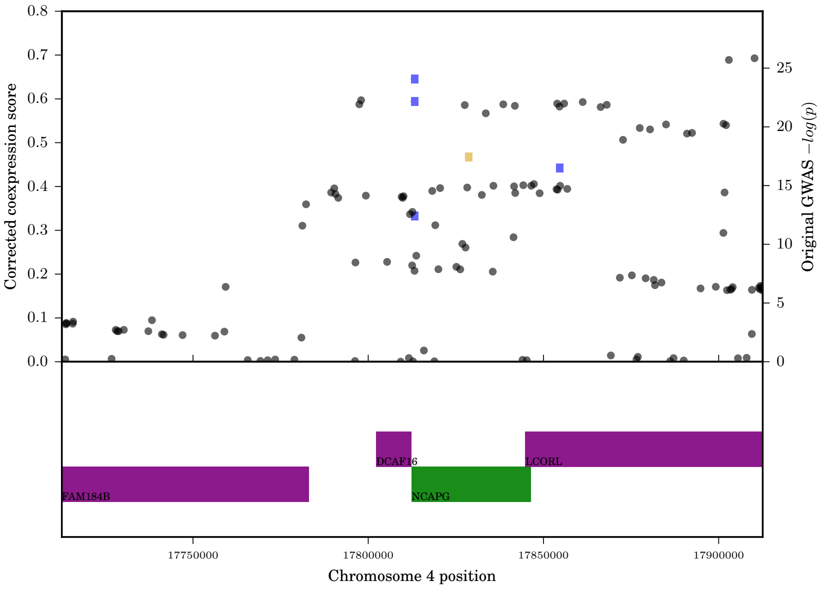

Region surrounding p1@NCAPG [rs11941723—rs2074974] p(Bonferroni)=0.00e+00.

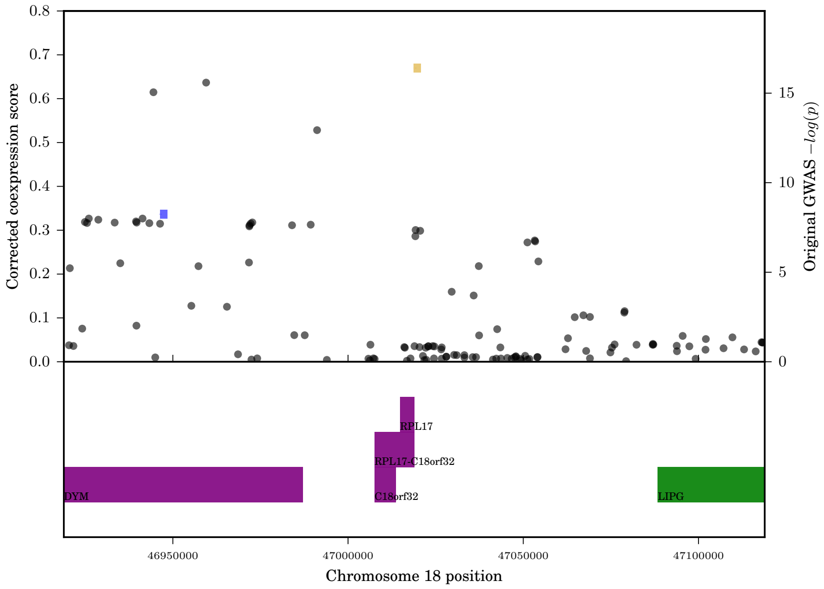

Region surrounding p3@RPL17 [rs3814888] p(Bonferroni)=0.00e+00.

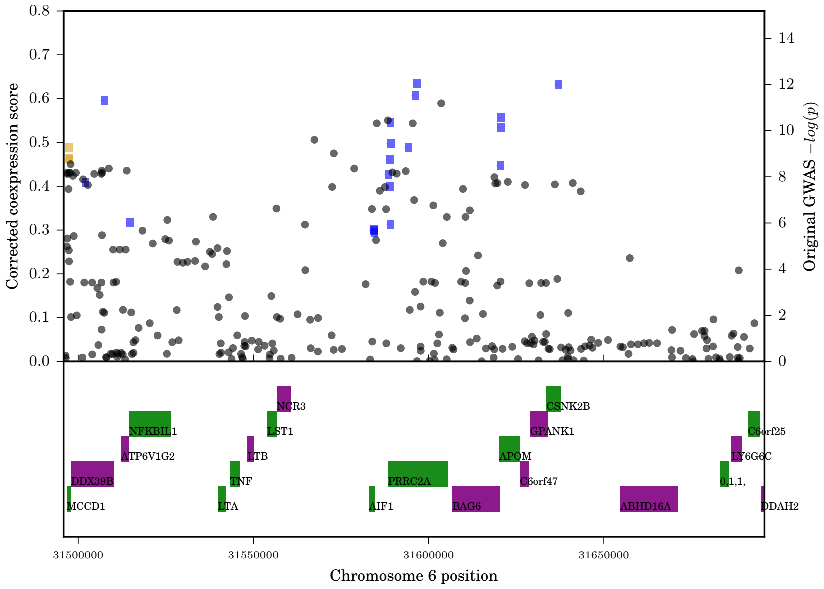

Region surrounding p@chr6:31595842..31595910,- [rs1046080] p(Bonferroni)=0.00e+00.

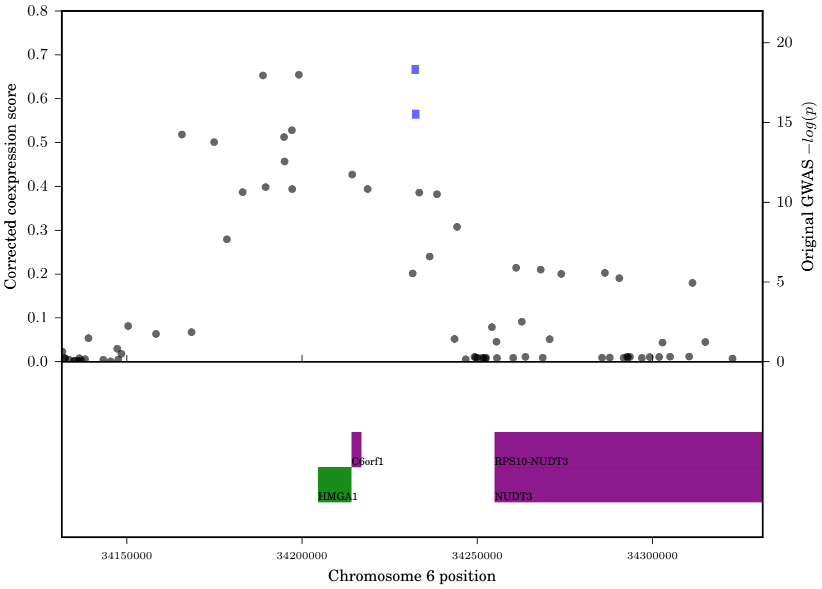

Region surrounding p1@RPL35P2 [rs6457769] p(Bonferroni)=0.00e+00.

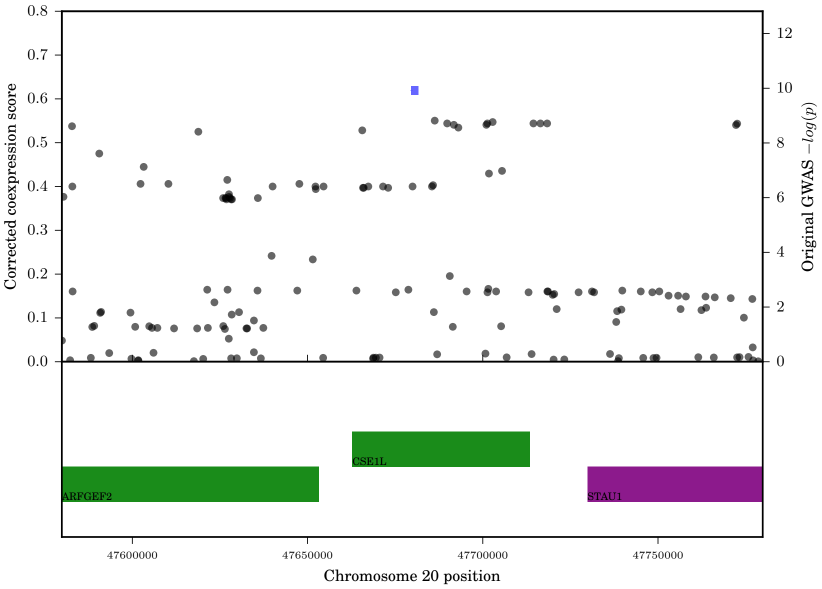

Region surrounding p@chr20:47679880..47679883,+ [rs6019621] p(Bonferroni)=0.00e+00.

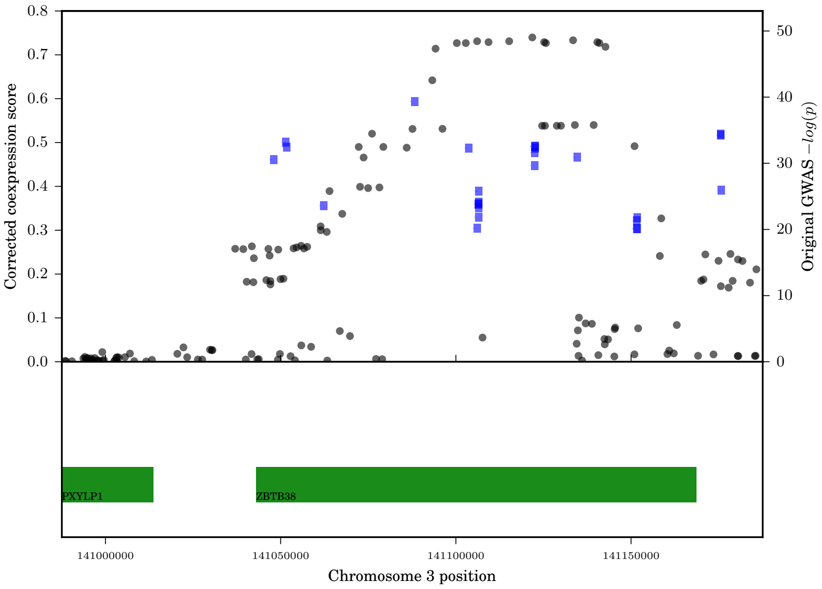

Region surrounding p12@ZBTB38 [rs1863868] p(Bonferroni)=0.00e+00.

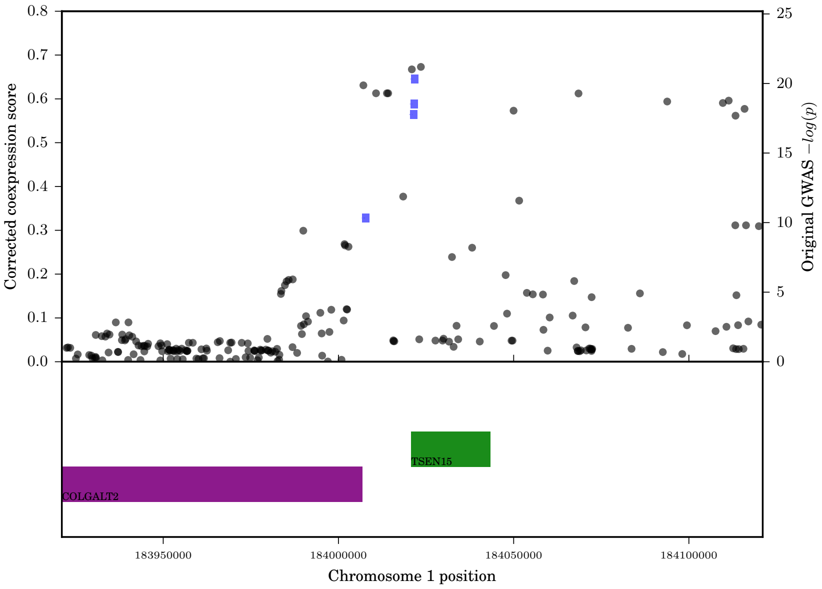

Region surrounding p@chr1:184021069..184021077,+ [rs2274432] p(Bonferroni)=0.00e+00.

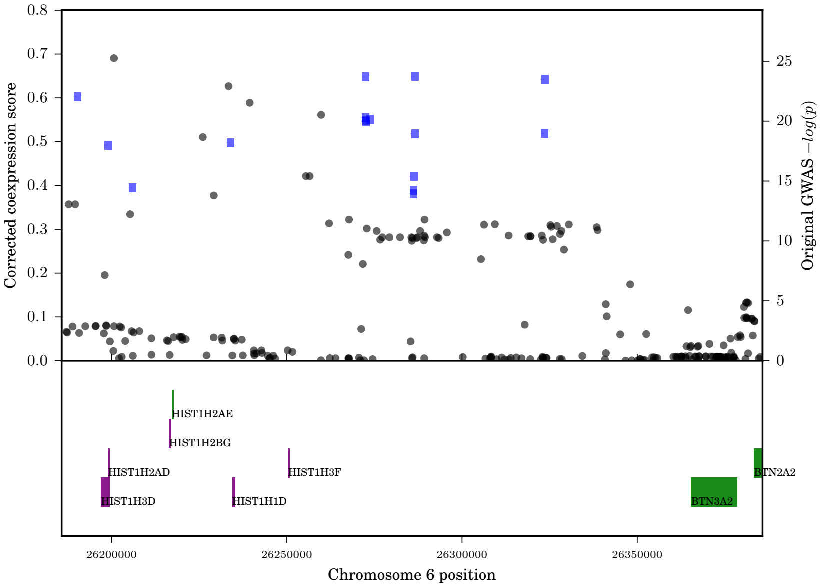

Region surrounding p4@HIST1H4A,p4@HIST1H4B,p4@HIST1H4D,p4@HIST1H4E,p4@HIST1H4F,p4@HIST1H4H,p4@HIST1H4I,p4@HIST1H4J,p4@[illegible]
[rs2393593—rs3999544] p(Bonferroni)=0.00e+00.

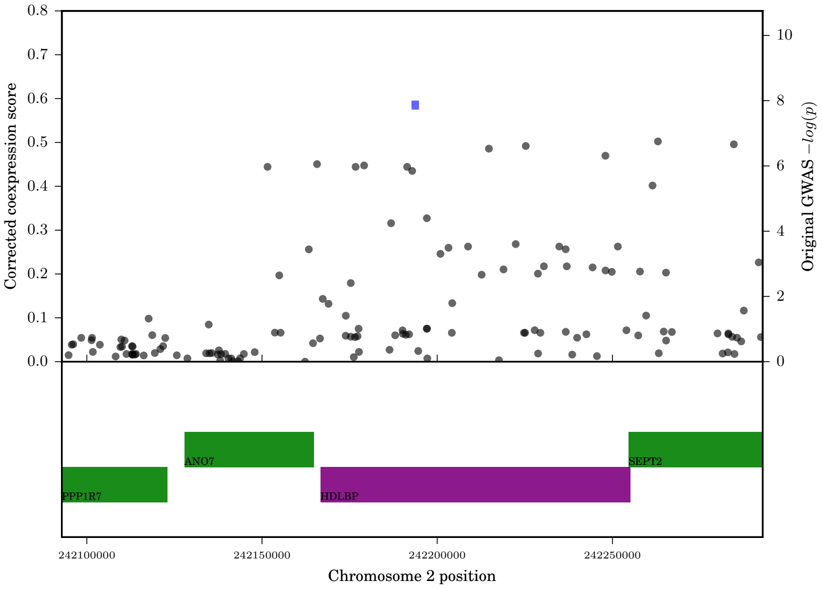

Region surrounding p@chr2:242192880..242192896,- [rs7578199] p(Bonferroni)=0.00e+00.

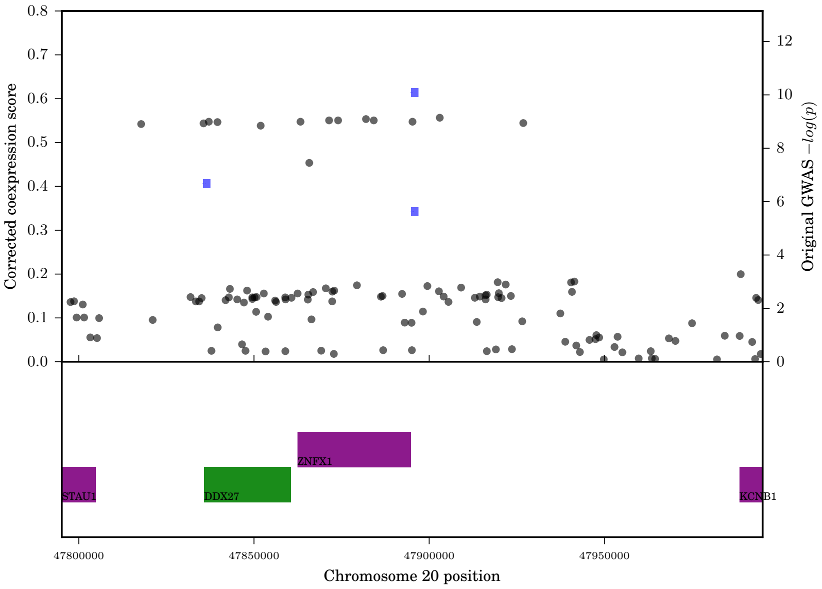

Region surrounding p1@ZNFX1-AS1 [rs6648] p(Bonferroni)=0.00e+00.

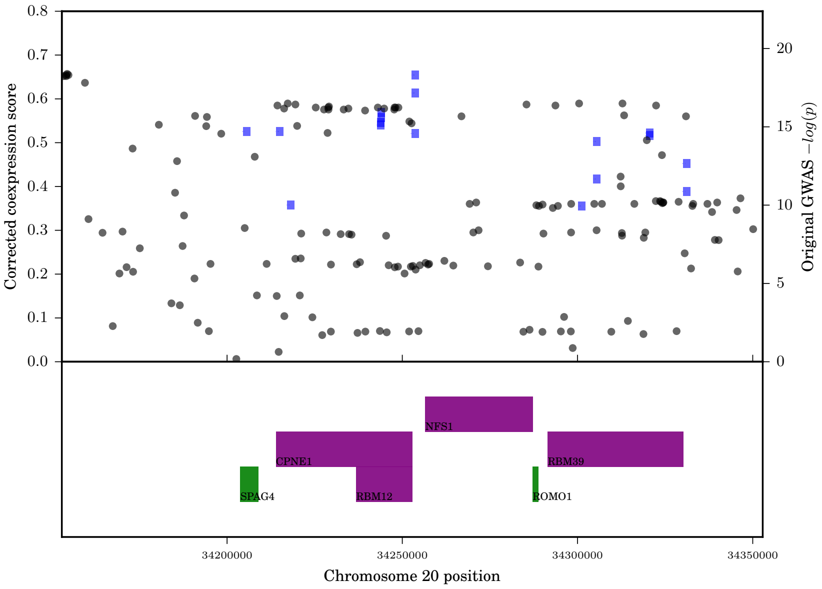

Region surrounding p2@CPNE1,p2@RBM12 [rs6119636] p(Bonferroni)=0.00e+00.

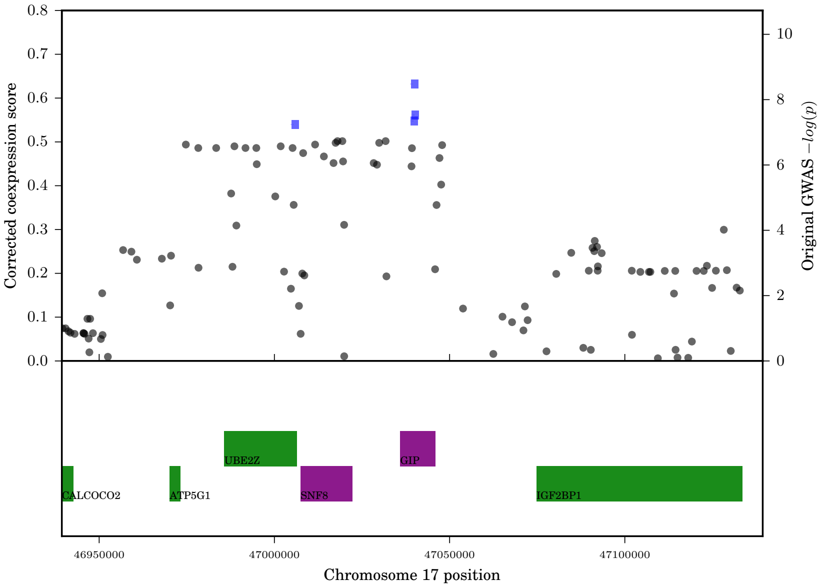

Region surrounding p@chr17:47039347..47039351,+ [rs2291725—rs2291726] p(Bonferroni)=0.00e+00.
