# Supplementary material for: Shared activity patterns arising at genetic susceptibility loci reveal underlying genomic and cellular architecture of human disease
Source: PLoS Comput Biol. 2018 Mar 1;14(3):e1005934. doi: 10.1371/journal.pcbi.1005934 (PMC5849332; doi:10.1371/journal.pcbi.1005934)
Supplement: S5 Table — (PDF) [file pcbi.1005934.s005.pdf]

# 1 Crohn's Disease

| Top promoter[SNPs in top promoter] | Linkage | Corrected coexpression score | Bonferroni-corrected p-value | FDR |
|---|---|---|---|---|
| p@chr5:131820136..131820148,- [rs2070727] | | 0.68 | 0 | 0.0 |
| enhancer@chr2:61112067-61112568 [rs6545835] | | 0.62 | 0 | 0.0 |
| p@chr1:67896878..67896883,+ [rs3762313, rs3762314] | | 0.61 | 0 | 0.0 |
| enhancer@chr5:40486540-40486993 [rs7720838] | | 0.57 | 0 | 0.0 |
| p4@TAP2 [rs241448, rs241447, rs241452, rs17034, rs241451, rs241449] | | 0.55 | 0 | 0.0 |
| enhancer@chr22:30591785-30593544 [rs713875] | | 0.52 | 0 | 0.0 |
| p@chr6:31240851..31240879,- [rs7759127] | | 0.52 | 0 | 0.0 |
| p@chr10:35484899..35484906,- [rs1057108] | | 0.49 | 0 | 0.0 |
| p@chr17:32635480..32635483,- [rs16969454] | | 0.48 | 0 | 0.0 |
| p@chr17:37970009..37970013,- [rs9909593] | | 0.46 | 0 | 0.0 |
| enhancer@chr15:67468171-67468380 [rs17294280] | | 0.44 | 0 | 0.0 |
| enhancer@chr14:88472465-88473193 [rs8005161] | | 0.43 | 0.023 | 0.0 |
| enhancer@chr10:82253401-82253904 [rs7900536] | | 0.41 | 0.069 | 0.0 |
| p1@DAP3 [rs1058207] | | 0.41 | 0.069 | 0.0 |
| p@chr6:32405128..32405175,- [rs3135395] | | 0.41 | 0.092 | 0.01 |
| p1@RPL37 [rs10065570] | | 0.38 | 0.14 | 0.01 |
| enhancer@chr21:45627966-45628150 [rs2838522] | | 0.38 | 0.16 | 0.01 |
| p@chr14:69261497..69261515,+ [rs2236262] | | 0.34 | 0.32 | 0.02 |
| p@chr2:43521231..43521252,+ [rs1322] | | 0.34 | 0.48 | 0.03 |
| p8@LNPEP [rs2351010] | | 0.32 | 0.62 | 0.03 |
| p1@C6orf48 [rs9368699] | | 0.32 | 0.64 | 0.03 |
| enhancer@chr1:206939730-206940044 [rs3024505] | | 0.32 | 0.64 | 0.03 |
| p3@CISD1 [rs2790189] | | 0.31 | 0.94 | 0.04 |
| p@chr2:102931037..102931044,+ [rs12712135] | | 0.29 | 1 | 0.08 |
| p@chr20:62366061..62366072,+ [rs2427533] | | 0.28 | 1 | 0.09 |
| p@chr10:64397428..64397447,- [rs16917546, rs10761652] | | 0.27 | 1 | 0.09 |
| p4@CD244 [rs12036607, rs7410867, rs12036670] | | 0.26 | 1 | 0.12 |
| p@chr1:7886661..7886674,+ [rs697693] | | 0.23 | 1 | 0.2 |
| p21@SEC16A [rs3812591] | | 0.23 | 1 | 0.2 |
| | rs3812591:rs3812584 D'=1.0; $r^2$=0.38. p3@PMPCA [rs3812584] | 0.17 | 1 | 0.36 |
| p@chr10:81053715..81053725,+ [rs1250560, rs1250559] | | 0.23 | 1 | 0.22 |
| enhancer@chr2:43745392-43745589 [rs10189235] | | 0.22 | 1 | 0.23 |
| p1@RNU86 [rs12484030] | | 0.22 | 1 | 0.23 |
| p1@AK131570 [rs9252] | | 0.22 | 1 | 0.23 |
| p3@SCAMP3 [rs1046188] | | 0.21 | 1 | 0.25 |
| p@chr3:49360037..49360041,- [rs9873994] | | 0.19 | 1 | 0.29 |
| p6@HIPK1 [rs3811019] | | 0.19 | 1 | 0.29 |
| enhancer@chr1:155951073-155951535 [rs1889532] | | 0.19 | 1 | 0.29 |
| enhancer@chr6:159514738-159514976 [rs654690] | | 0.19 | 1 | 0.31 |
| p@chr6:31543810..31543821,- [rs3093661] | | 0.18 | 1 | 0.33 |
| p3@TNXB [rs8283] | | 0.18 | 1 | 0.34 |
| enhancer@chr6:167411692-167411848 [rs239935] | | 0.18 | 1 | 0.34 |
| p1@ENST00000431069 [rs3780373] | | 0.17 | 1 | 0.35 |
| enhancer@chr1:155433776-155433971 [rs12724079] | | 0.16 | 1 | 0.38 |
| p7@RBM6 [rs6772095] | | 0.16 | 1 | 0.41 |
| p2@uc001flf.2 [rs2048431] | | 0.15 | 1 | 0.44 |
| p@chr6:32186154..32186158,- [rs438475] | | 0.14 | 1 | 0.46 |
| p@chr1:200877847..200877851,+ [rs7554511] | | 0.13 | 1 | 0.54 |
| enhancer@chr6:20811412-20811642 [rs9465900] | | 0.12 | 1 | 0.55 |
| p2@ATP2A1 [rs3888190] | | 0.12 | 1 | 0.55 |
| p14@FADS1 [rs174548] | | 0.12 | 1 | 0.55 |
| p1@RBM5 [rs2247510] | | 0.12 | 1 | 0.55 |
| p3@GON4L [rs3820594] | | 0.1 | 1 | 0.62 |
| chr16:50668341..50668342,+ [rs12913] | | 0.09 | 1 | 0.62 |
| enhancer@chr5:158848054-158848257 [rs4921496] | | 0.09 | 1 | 0.62 |
| p4@SLC9A4 [rs4851011] | | 0.09 | 1 | 0.63 |
| p1@ZNF300 [rs7724036] | | 0.09 | 1 | 0.63 |
| p@chr5:158705320..158705331,+ [rs12520035] | | 0.09 | 1 | 0.64 |
| p13@DGKD [rs838718] | | 0.08 | 1 | 0.65 |
| p2@TNFRSF6B [rs2297441] | | 0.08 | 1 | 0.68 |
| | rs2297441:rs11696871 D'=0.96; $r^2$=0.61. chr20:62387380..62387381,- [rs11696871] | 0.01 | 1 | 0.97 |
| p2@NKX2-3 [rs10883371] | | 0.06 | 1 | 0.74 |
| p5@LRRK2 [rs1388597] | | 0.05 | 1 | 0.8 |
| p1@SCARNA5 [rs3792109] | | 0.04 | 1 | 0.86 |
| p@chr3:49570849..49570853,+ [rs1050088] | | 0.03 | 1 | 0.91 |
| | rs1050088:rs3811697 D'=1.0; $r^2$=0.32. p@chr3:49591052..49591057,+ [rs3811697] | 0.02 | 1 | 0.92 |
| p9@EBF1 [rs7442701] | | 0.03 | 1 | 0.91 |
| enhancer@chr16:50554457-50554868 [rs12930566] | | 0.02 | 1 | 0.92 |
| p1@GTF2H4 [rs2074510] | | 0.02 | 1 | 0.92 |
| p@chr6:134637878..134637882,- [rs6923743] | | 0.0 | 1 | 1.0 |

| Cell type | RRA p | FDR |
|---|---|---|
| CD14pos Monocytes | 1.8e-17 | 0 |
| Peripheral Blood Mononuclear Cells | 2.8e-14 | 0 |
| CD14 monocytes treated with Trehalose | 4.7e-14 | 0 |
| CD14 monocytes treated with BCG | 7.4e-14 | 0 |
| CD14 monocytes treated with Candida | 8.2e-14 | 0 |
| CD14 monocytes treated with Group A streptococci | 9.5e-13 | 0 |
| CD14 monocytes treated with Salmonella | 9.7e-13 | 0 |
| Basophils | 1.7e-12 | 0 |
| CD14 monocytes treated with IFN Nhexane | 2e-09 | 0 |
| CD14pos CD16pos Monocytes | 2e-09 | 0 |
| CD4 CD25CD45RA memory conventional T cells | 1.5e-08 | 0 |
| Natural Killer Cells | 7.3e-08 | 0 |
| CD4 T Cells | 7.4e-07 | 0 |
| CD14neg CD16pos Monocytes | 3.7e-05 | 0 |
| CD14 monocytes treated with lipopolysaccharide | 8e-12 | 0.0039 |
| CD8 T Cells pluriselect | 5.6e-05 | 0.0073 |
| CD34 Progenitors | 1.4e-06 | 0.0087 |
| CD19 B Cells | 1.1e-05 | 0.0087 |
| CD14 monocytes mock treated | 3.4e-05 | 0.0087 |
| Dendritic Cells plasmacytoid | 0.00069 | 0.0087 |
| CD14 monocytes treated with Bglucan | 6.6e-08 | 0.011 |
| CD14 monocytes treated with Cryptococcus | 1e-06 | 0.015 |

| | | |
|---|---|---|
| CD4 CD25 CD45RA memory regulatory T cells expanded | 0.0085 | 0.015 |
| CD8 T Cells | 3.2e-05 | 0.019 |
| CD14pos CD16neg Monocytes | 0.00072 | 0.021 |
| CD4 CD25 CD45RA memory regulatory T cells | 0.0017 | 0.039 |

## 1.1 Crohn's disease subgroup 1

| Cell type | RRA p | FDR |
|---|---|---|
| CD14pos Monocytes | 0 | 0 |
| Peripheral Blood Mononuclear Cells | 0 | 0 |
| CD14 monocytes treated with Trehalose | 0 | 0 |
| Basophils | 0 | 0 |
| CD14 monocytes treated with BCG | 0 | 0 |
| CD14 monocytes treated with Salmonella | 0 | 0 |
| CD14 monocytes treated with Candida | 0 | 0 |
| CD14pos CD16pos Monocytes | 0 | 0 |
| CD14 monocytes treated with IFN Nhexane | 0 | 0 |
| Natural Killer Cells | 0 | 0 |
| CD4 CD25CD45RA memory conventional T cells | 0 | 0 |
| CD14neg CD16pos Monocytes | 0 | 0 |
| CD4 T Cells | 1e-05 | 0.00039 |
| CD19 B Cells | 1e-05 | 0.00039 |
| Dendritic Cells plasmacytoid | 2e-05 | 0.00072 |
| CD14 monocytes treated with Bglucan | 3e-05 | 0.001 |
| CD14 monocytes treated with Cryptococcus | 5e-05 | 0.0016 |
| CD14 monocytes treated with lipopolysaccharide | 7e-05 | 0.0021 |
| CD14 monocytes mock treated | 8e-05 | 0.0023 |
| CD34 Progenitors | 9e-05 | 0.0024 |
| CD4 CD25 CD45RA memory regulatory T cells expanded | 0.0001 | 0.0026 |
| CD14pos CD16neg Monocytes | 0.00015 | 0.0037 |
| CD14 monocytes treated with Group A streptococci | 0.00018 | 0.0042 |
| CD8 T Cells pluriselect | 0.00033 | 0.0073 |
| CD8 T Cells | 0.00034 | 0.0073 |
| macLPS 24hr | 0.00044 | 0.0091 |
| COBLa rinderpestC infection 48hr | 0.00062 | 0.012 |
| immature langerhans cells | 0.00067 | 0.013 |
| CD4 CD25 CD45RA memory regulatory T cells | 0.00074 | 0.014 |
| gamma delta positive T cells | 0.00088 | 0.016 |
| Neutrophils | 0.00099 | 0.017 |
| Mast cell expanded and stimulated | 0.0012 | 0.019 |
| macLPS 22hr | 0.0012 | 0.019 |

## 1.2 Crohn's disease subgroup 2

| Cell type | RRA p | FDR |
|---|---|---|
| Esophageal Epithelial Cells | 1e-05 | 0.0058 |
| K562 erythroblastic leukemia response to hemin 06hr | 0.00024 | 0.043 |
| mesenchymalstemcells(adiposederived)adipogenicinduction 00hr30min | 0.00048 | 0.043 |
| CD133 stem cells cord blood derived pool1 | 0.0006 | 0.043 |
| Smooth Muscle Cells Aortic(cytoplasm) | 0.00066 | 0.043 |
| Mesenchymal Stem Cells umbilical | 0.00069 | 0.043 |
| K562 erythroblastic leukemia response to hemin 24hr | 0.00072 | 0.043 |
| K562 erythroblastic leukemia response to hemin 02hr30min | 0.00074 | 0.043 |
| K562 erythroblastic leukemia response to hemin 03hr00min | 0.00077 | 0.043 |
| Fibroblast Aortic Adventitial(cytoplasmic) | 0.00084 | 0.043 |
| K562 erythroblastic leukemia response to hemin day03 | 0.00094 | 0.043 |
| Keratocytes | 0.00098 | 0.043 |
| Trabecular Meshwork Cells | 0.001 | 0.043 |
| K562 erythroblastic leukemia response to hemin 01hr00min | 0.0011 | 0.043 |
| K562 erythroblastic leukemia response to hemin 03hr30min | 0.0012 | 0.043 |
| Hep2 cells treated with Streptococci strain JRS4 | 0.0013 | 0.043 |
| K562 erythroblastic leukemia response to hemin 12hr | 0.0013 | 0.043 |
| K562 erythroblastic leukemia response to hemin 02hr00min | 0.0013 | 0.043 |
| K562 erythroblastic leukemia response to hemin 01hr20min | 0.0016 | 0.048 |
| K562 erythroblastic leukemia response to hemin day04 | 0.0016 | 0.048 |

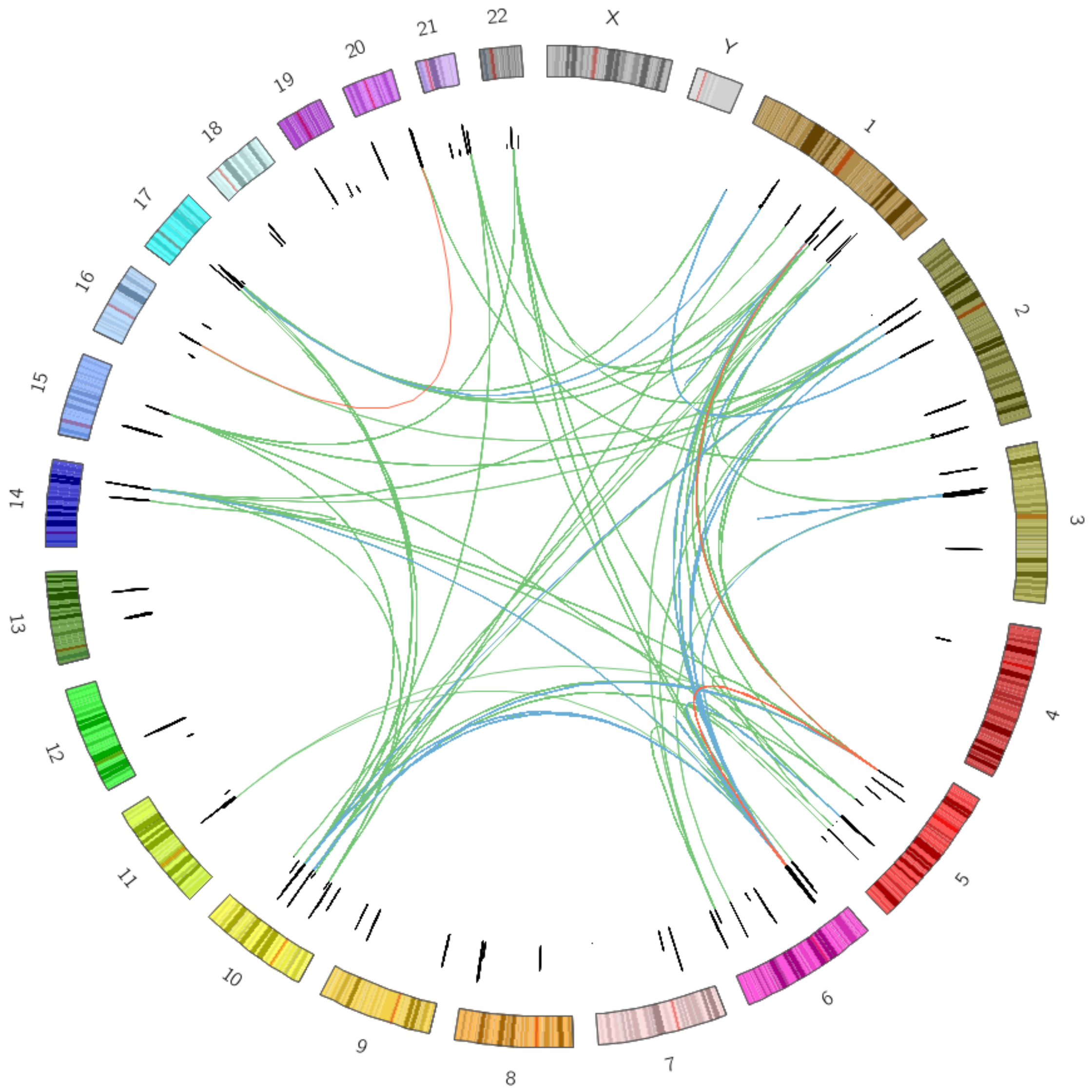


Figure 1: Circular plot of coexpression links between different locations on the genome (Crohn's Disease). Colour indicates $log_{10}(p)$: red>3, blue> 2, green> 1.5). See main manuscript for full explanation.

# 2 Ulcerative Colitis

| Top promoter[SNPs in top promoter] | Linkage | Corrected coexpression score | Bonferroni-corrected p-value | FDR |
|---|---|---|---|---|
| p1@TRIM40 [rs9261467] | | 0.67 | 0 | 0.0 |
| | rs9261467:rs9295829 D'=0.84; $r^2$=0.6. p3@ZNRD1-AS1 [rs9295829] | 0.33 | 1 | 0.05 |
| | rs9261467:rs9295829 D'=0.84; $r^2$=0.6. p2@ZNRD1 [rs9295829] | 0.16 | 1 | 0.45 |
| p4@RNF186 [rs12064796] | | 0.61 | 0 | 0.0 |
| p2@HLA-DQA1 [rs9272426] | | 0.6 | 0 | 0.0 |
| p11@C1orf106 [rs7554511] | | 0.56 | 0 | 0.0 |
| | same SNP, different promoter: p@chr1:200877847..200877851,+ [rs7554511] | 0.19 | 1 | 0.37 |
| | rs7554511:rs296533 D'=0.73; $r^2$=0.4. enhancer@chr1:200865457-200866265 [rs296533] | 0.08 | 1 | 0.73 |
| p1@LOC100133445, p1@LOC115110 [rs1886730] | | 0.54 | 0 | 0.0 |
| p3@HLA-DQA1 [rs3135391] | | 0.51 | 0 | 0.0 |
| | rs3135391:rs2050188 D'=1.0; $r^2$=0.47. p2@C6orf10 [rs2050188] | 0.28 | 1 | 0.15 |
| p@chr20:62366043..62366057,+ [rs2427533] | | 0.48 | 0 | 0.0 |
| p@chr11:6461452..6461464,- [rs10839564] | | 0.46 | 0 | 0.0 |
| p2@LSP1 [rs907611] | | 0.45 | 0 | 0.0 |
| p2@FAM55A [rs661946] | | 0.44 | 0 | 0.0 |
| p3@X07061, p3@X07062 [rs1058026] | | 0.4 | 0.12 | 0.01 |
| p2@NKX2-3 [rs10883371, rs10883373] | | 0.4 | 0.12 | 0.01 |
| p@chr1:206943938..206943954,- [rs3024493] | | 0.39 | 0.12 | 0.01 |
| p27@CFB [rs1048709] | | 0.37 | 0.27 | 0.02 |
| p13@TMBIM1 [rs2382817] | | 0.36 | 0.39 | 0.03 |
| p@chr17:38029166..38029196,+ [rs12936231] | | 0.36 | 0.41 | 0.03 |
| p5@CCNY [rs12242110] | | 0.36 | 0.43 | 0.03 |
| p4@PMPCA [rs3812584] | | 0.36 | 0.59 | 0.03 |
| | same SNP, different promoter: p3@PMPCA [rs3812584] | 0.29 | 1 | 0.13 |
| enhancer@chr1:151792678-151793001 [rs949969] | | 0.34 | 0.84 | 0.04 |
| p7@CREM [rs4934730] | | 0.34 | 0.88 | 0.04 |
| p4@CXCR2 [rs4674258] | | 0.33 | 1 | 0.05 |
| p28@TNIP1 [rs2233287] | | 0.31 | 1 | 0.09 |
| p4@MZB1 [rs9327847] | | 0.3 | 1 | 0.11 |
| p@chr1:8022209..8022235,- [rs3766606] | | 0.3 | 1 | 0.11 |
| p@chr6:31464084..31464111,- [rs6916394] | | 0.29 | 1 | 0.12 |
| | rs6916394:rs3828912 D'=1.0; $r^2$=1.0. p@chr6:31465753..31465756,- [rs3828912, rs3828914];rs6916394:rs3828914 D'=1.0; $r^2$=1.0. p@chr6:31465753..31465756,- [rs3828912, rs3828914] | 0.04 | 1 | 0.89 |
| p@chr5:498417..498421,- [rs12521198] | | 0.29 | 1 | 0.12 |
| enhancer@chr14:98513457-98513851 [rs8008845] | | 0.27 | 1 | 0.16 |
| enhancer@chr2:61095090-61095322 [rs842639] | | 0.27 | 1 | 0.17 |
| p1@SLC26A3 [rs2301988, rs2301989] | | 0.26 | 1 | 0.19 |
| p15@GAL3ST1 [rs7286285] | | 0.25 | 1 | 0.21 |
| enhancer@chr18:4003748-4003994 [rs16945853] | | 0.25 | 1 | 0.21 |
| enhancer@chr3:49389809-49389972 [rs17080528] | | 0.23 | 1 | 0.27 |
| | rs17080528:rs11716445 D'=1.0; $r^2$=0.3. p@chr3:49405916..49405955,- [rs11716445] | 0.05 | 1 | 0.83 |
| enhancer@chr5:601191-601577 [rs3749615] | | 0.23 | 1 | 0.27 |
| | rs3749615:rs7434 D'=1.0; $r^2$=1.0. p@chr5:660645..660655,- [rs7434] | 0.14 | 1 | 0.5 |
| enhancer@chr2:61204524-61204872 [rs7608910] | | 0.23 | 1 | 0.27 |
| p@chr6:32186154..32186158,- [rs436388] | | 0.23 | 1 | 0.27 |
| | rs436388:rs176095 D'=1.0; $r^2$=0.21. p@chr6:32158228..32158249,+ [rs176095] | 0.19 | 1 | 0.37 |
| | rs436388:rs3134947 D'=0.92; $r^2$=0.18. p2@AGPAT1 [rs3134947] | 0.03 | 1 | 0.91 |
| p1@GNA12 [rs1636249] | | 0.23 | 1 | 0.27 |
| enhancer@chr5:40410103-40410947 [rs6451493, rs11742570] | | 0.21 | 1 | 0.32 |
| p@chr3:49139102..49139119,- [rs35673421] | | 0.21 | 1 | 0.32 |
| chr2:28203328..28203338,+ [rs898031] | | 0.21 | 1 | 0.32 |
| p1@ENST00000448198, p1@ENST00000455328 [rs3916765] | | 0.21 | 1 | 0.32 |
| p@chr8:135520294..135520308,- [rs16905158] | | 0.21 | 1 | 0.32 |
| enhancer@chr6:42081758-42081907 [rs9462770] | | 0.2 | 1 | 0.33 |
| p1@RBM5 [rs2247510] | | 0.19 | 1 | 0.37 |
| p11@PNMT [rs876493] | | 0.19 | 1 | 0.37 |
| p2@TNFRSF6B [rs2297441] | | 0.19 | 1 | 0.37 |
| enhancer@chr13:37723502-37723846 [rs17054986] | | 0.19 | 1 | 0.37 |
| p@chr3:49591052..49591057,+ [rs3811697] | | 0.18 | 1 | 0.39 |
| p@chr6:29635769..29635797,+ [rs1318631] | | 0.18 | 1 | 0.4 |
| p9@MMP23B [rs28456011] | | 0.16 | 1 | 0.45 |
| chr3:49941317..49941322,- [rs2280406] | | 0.16 | 1 | 0.45 |
| | rs2280406:rs6772095 D'=1.0; $r^2$=0.87. p7@RBM6 [rs6772095] | 0.1 | 1 | 0.69 |
| enhancer@chr21:40466283-40466718 [rs2836882] | | 0.15 | 1 | 0.47 |
| p@chr4:148535454..148535476,- [rs931779] | | 0.15 | 1 | 0.49 |
| p@chr1:110364218..110364229,+ [rs2938616] | | 0.13 | 1 | 0.56 |
| p@chr5:149627361..149627384,- [rs7711562] | | 0.12 | 1 | 0.61 |
| p@chr12:7032510..7032525,+ [rs10849541] | | 0.11 | 1 | 0.63 |
| enhancer@chr1:36715455-36715834 [rs11263868] | | 0.1 | 1 | 0.68 |
| enhancer@chr5:158826036-158826401 [rs918519] | | 0.1 | 1 | 0.68 |
| p4@PRKAR1B [rs4074132] | | 0.1 | 1 | 0.68 |
| p1@ENST00000431069 [rs3780373] | | 0.09 | 1 | 0.73 |
| enhancer@chr6:30716597-30717062 [rs12210092] | | 0.08 | 1 | 0.73 |
| enhancer@chr3:138565329-138565569 [rs11716652] | | 0.08 | 1 | 0.73 |
| chr2:127816629..127816649,+ [rs17014835] | | 0.07 | 1 | 0.79 |
| p1@MLH3 [rs175083] | | 0.06 | 1 | 0.79 |
| p12@LAMB1 [rs2158836] | | 0.06 | 1 | 0.79 |
| enhancer@chr17:25691108-25691369 [rs322207] | | 0.06 | 1 | 0.82 |
| enhancer@chr7:22711367-22711465 [rs17147141] | | 0.04 | 1 | 0.89 |
| p1@ZFP90 [rs1170445, rs1170444] | | 0.03 | 1 | 0.9 |
| enhancer@chr12:8147722-8148111 [rs10161016] | | 0.03 | 1 | 0.9 |
| p1@SLC38A3 [rs1858828] | | 0.01 | 1 | 0.97 |
| p@chr13:77463947..77463954,- [rs17066875] | | 0.0 | 1 | 1.0 |

| | | | | |
|---|---|---|---|---|
| p2@RPL22 [rs4908865] | | 0.0 | 1 | 1.0 |

| Cell type | RRA p | FDR |
|---|---|---|
| small intestine adult pool1 | 9.7e-07 | 0 |
| liver adult pool1 | 5e-06 | 0 |
| macLPS 04hr | 0.00011 | 0 |
| colon adult pool1 | 0.00019 | 0 |
| appendix adult | 0.00026 | 0 |
| rectum fetal | 0.00053 | 0 |
| duodenum fetal donor1 tech | 3.3e-05 | 0.0073 |
| macLPS 05hr | 9.8e-05 | 0.0073 |
| small intestine fetal | 0.0075 | 0.012 |
| macLPS 22hr | 0.026 | 0.012 |
| macLPS 12hr | 0.013 | 0.015 |
| spleen adult pool1 | 0.041 | 0.015 |
| macLPS 18hr | 0.019 | 0.018 |
| colon fetal | 0.012 | 0.033 |
| macLPS 10hr | 0.006 | 0.035 |
| macLPS 03hr30min | 0.0022 | 0.036 |
| macLPS 24hr | 0.035 | 0.036 |
| macLPS 20hr | 0.036 | 0.036 |

## 2.1 Ulcerative colitis subgroup 1

| Cell type | RRA p | FDR |
|---|---|---|
| macLPS 04hr | 0 | 0 |
| macLPS 10hr | 0 | 0 |
| macLPS 03hr30min | 0 | 0 |
| macLPS 05hr | 0 | 0 |
| macLPS 02hr30min | 0 | 0 |
| macLPS 00hr45min | 0 | 0 |
| macLPS 03hr00min | 0 | 0 |
| macLPS 07hr | 0 | 0 |
| macLPS 01hr20min | 0 | 0 |
| macLPS 02hr00min | 0 | 0 |
| macLPS 12hr | 0 | 0 |
| macLPS 01hr40min | 0 | 0 |
| macLPS 24hr | 0 | 0 |
| macLPS 08hr | 0 | 0 |
| macLPS 00hr00min | 0 | 0 |
| macLPS 20hr | 0 | 0 |
| macLPS 18hr | 0 | 0 |
| macLPS 22hr | 0 | 0 |
| macLPS 48hr | 0 | 0 |
| macLPS 14hr | 0 | 0 |
| spleen adult pool1 | 0 | 0 |
| macLPS 06hr | 1e-05 | 0.00023 |
| macLPS 16hr | 1e-05 | 0.00023 |
| macLPS 36hr | 1e-05 | 0.00023 |
| CD14 monocyte derived endothelial progenitor cells | 1e-05 | 0.00023 |
| appendix adult | 2e-05 | 0.00042 |
| Macrophage monocyte derived | 2e-05 | 0.00042 |
| macLPS 00hr15min | 4e-05 | 0.00081 |
| macmockFLU 00hr00min | 6e-05 | 0.0011 |
| macLPS 00hr30min | 6e-05 | 0.0011 |
| CD4 CD25 CD45RA memory regulatory T cells expanded | 9e-05 | 0.0016 |
| gamma delta positive T cells | 0.0001 | 0.0017 |
| Dendritic Cells monocyte immature derived donor1 tech | 0.0001 | 0.0017 |
| COBLa rinderpest infection 24hr | 0.00011 | 0.0018 |
| macLPS 01hr00min | 0.00012 | 0.0019 |
| macFLU 02hr00min | 0.00031 | 0.0049 |
| migratory langerhans cells | 0.00033 | 0.005 |
| macmockFLU 24hr00min | 0.00035 | 0.0052 |
| COBLa rinderpestC infection 24hr | 0.0004 | 0.0058 |
| CD8 T Cells pluriselect | 0.00044 | 0.0062 |
| COBLa rinderpest infection 12hr | 0.00045 | 0.0062 |
| Dendritic Cells monocyte immature derived | 0.00053 | 0.0071 |
| CD4 CD25 CD45RA memory regulatory T cells | 0.00057 | 0.0075 |
| CD14 monocytes treated with Bglucan | 0.0007 | 0.009 |
| CD14 monocytes mock treated | 0.00074 | 0.0093 |
| COBLa rinderpestC infection 06hr | 0.00076 | 0.0094 |
| CD14 monocytes treated with BCG | 0.00078 | 0.0094 |
| CD14 monocytes treated with Cryptococcus | 0.00095 | 0.011 |
| Adipocyte differentiation day04 | 0.001 | 0.012 |
| trachea adult pool1 | 0.0011 | 0.013 |
| colon adult pool1 | 0.0012 | 0.013 |
| CD14pos CD16pos Monocytes | 0.0014 | 0.015 |
| CD4 CD25CD45RA memory conventional T cells expanded | 0.0014 | 0.015 |
| CD14 monocytes treated with IFN Nhexane | 0.0015 | 0.015 |
| CD14 monocytes treated with Group A streptococci | 0.0016 | 0.017 |
| CD19 B Cells pluriselect | 0.0017 | 0.017 |
| immature langerhans cells | 0.0019 | 0.019 |
| CD14 monocytes treated with lipopolysaccharide | 0.0021 | 0.02 |
| Adipocyte differentiation day12 | 0.0021 | 0.02 |
| CD14neg CD16pos Monocytes | 0.0021 | 0.02 |
| Peripheral Blood Mononuclear Cells | 0.0022 | 0.02 |
| spleen fetal pool1 | 0.0023 | 0.021 |
| COBLa rinderpest infection 00hr | 0.0024 | 0.022 |
| macFLU 00hr00min | 0.0025 | 0.022 |
| aorta adult pool1 | 0.0032 | 0.027 |
| CD14 monocytes treated with Trehalose | 0.0032 | 0.027 |
| Natural Killer Cells | 0.0032 | 0.027 |
| COBLa rinderpestC infection 12hr | 0.0036 | 0.03 |
| spinal cord adult | 0.0038 | 0.03 |
| CD4 CD25 CD45RA naive regulatory T cells expanded | 0.0038 | 0.03 |
| Adipocyte differentiation day08 | 0.0043 | 0.034 |
| CD19 B Cells | 0.0048 | 0.038 |
| CD14pos CD16neg Monocytes | 0.0049 | 0.038 |
| CD14 monocytes treated with Candida | 0.005 | 0.039 |
| blood adult pool1 | 0.006 | 0.045 |
| CD14 monocytes treated with Salmonella | 0.0061 | 0.046 |
| Myoblast differentiation to myotubes day04 control | 0.0063 | 0.046 |
| aorticSMC responsetoFGF2 0h 00hr00min | 0.0067 | 0.048 |
| lung right lower lobe adult | 0.0068 | 0.048 |

| | | |
|---|---|---|
| tonsil adult pool1 | 0.0068 | 0.048 |
| macFLU 07hr00min | 0.0069 | 0.048 |

## 2.2 Ulcerative colitis subgroup 2

| Cell type | RRA p | FDR |
|---|---|---|
| small intestine adult pool1 | 0 | 0 |
| liver adult pool1 | 1e-05 | 0.00074 |
| colon adult pool1 | 6e-05 | 0.0029 |
| duodenum fetal donor1 tech | 0.00098 | 0.029 |
| appendix adult | 0.001 | 0.029 |
| colon fetal | 0.0019 | 0.046 |

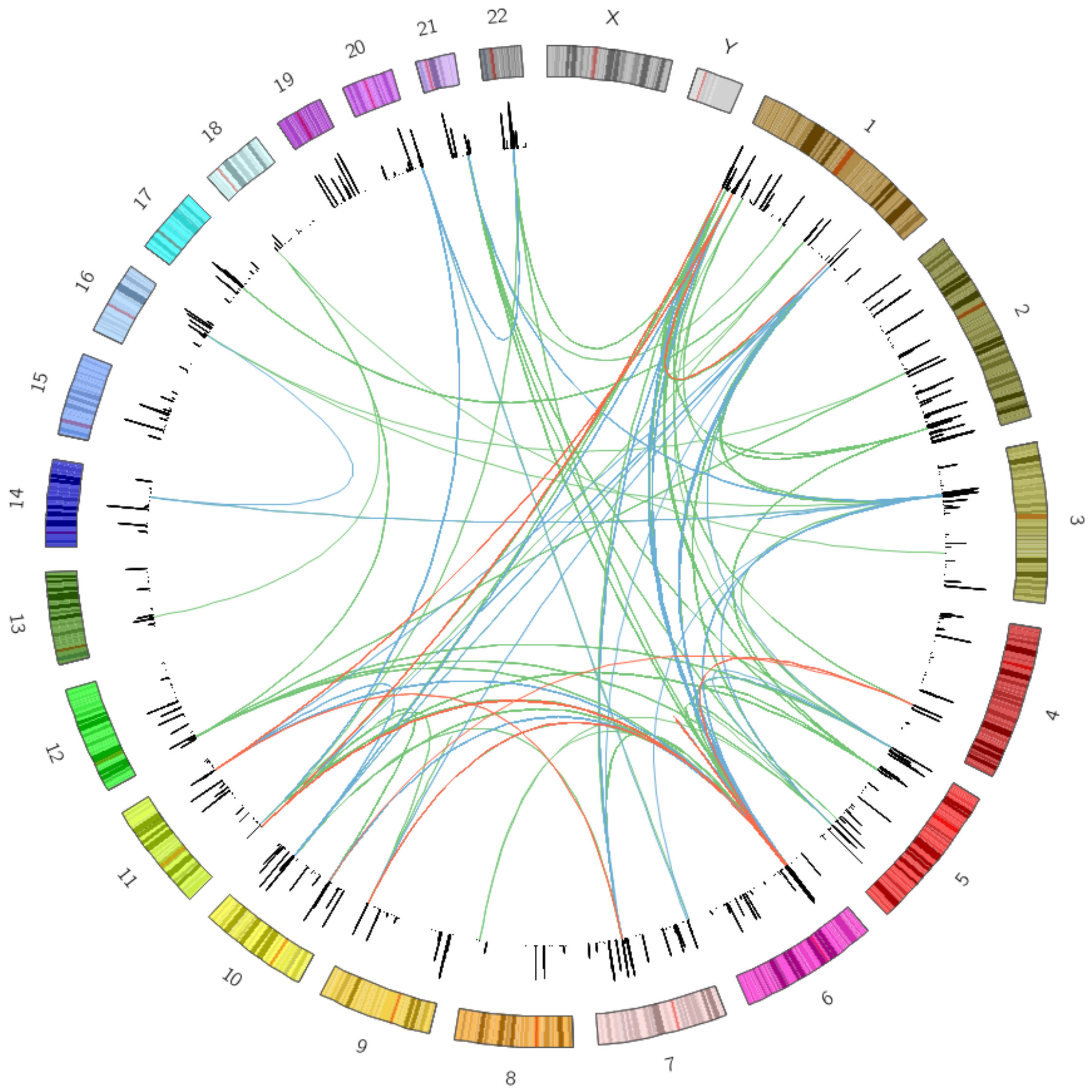


Figure 2: Circular plot of coexpression links between different locations on the genome (Ulcerative Colitis). Colour indicates $log_{10}(p)$: red>3, blue> 2, green> 1.5). See main manuscript for full explanation.

# 3 HDL Cholesterol

| Top promoter[SNPs in top promoter] | Linkage | Corrected coexpression score | Bonferroni-corrected p-value | FDR |
|---|---|---|---|---|
| p@chr9:107669326..107669336,+ [rs4100654, rs13284054] | | 0.71 | 0 | 0.0 |
| p2@ENST00000518619 [rs9987289, rs4841132] | | 0.67 | 0 | 0.0 |
| | rs9987289:rs7357361 D'=1.0; $r^2$=0.57. enhancer@chr8:9219931-9220273 [rs7357361];rs4841132:rs7357361 D'=1.0; $r^2$=0.57. enhancer@chr8:9219931-9220273 [rs7357361] | 0.1 | 1 | 0.76 |
| chr11:47281010..47281020,+ [rs11039155] | | 0.64 | 0 | 0.0 |
| p13@BC172787 [rs693] | | 0.64 | 0 | 0.0 |
| p1@SLC17A3 [rs13198474] | | 0.6 | 0 | 0.0 |
| p@chr18:47176875..47176889,+ [rs4939886, rs4939887, rs4939888] | | 0.55 | 0 | 0.0 |
| p@chr19:45411878..45411884,- [rs7412] | | 0.52 | 0.016 | 0.0 |
| p2@F2 [rs2070852, rs5896] | | 0.51 | 0.016 | 0.0 |
| | rs2070852:rs4752927 D'=1.0; $r^2$=0.29. p@chr11:46702222..46702264,- [rs4752927];rs5896:rs4752927 D'=1.0; $r^2$=0.84. p@chr11:46702222..46702264,- [rs4752927] | 0.26 | 1 | 0.38 |
| p1@APOA5 [rs651821] | | 0.5 | 0.032 | 0.0 |
| | rs651821:rs11216164 D'=1.0; $r^2$=0.06. p@chr11:116734451..116734464,- [rs11216164] | 0.09 | 1 | 0.79 |
| p8@ALDH1A2 [rs1122208] | | 0.48 | 0.063 | 0.01 |
| chr8:19824080..19824096,+ [rs1059507, rs3735964] | | 0.48 | 0.079 | 0.01 |
| | rs3735964:rs1569209 D'=1.0; $r^2$=0.87. p@chr8:19830089..19830095,+ [rs1569209] | 0.29 | 1 | 0.29 |
| enhancer@chr7:73037168-73037552 [rs55747707] | | 0.48 | 0.079 | 0.01 |
| p2@ENST00000519197 [rs28597716] | | 0.48 | 0.095 | 0.01 |
| p@chr2:165603312..165603317,- [rs10178921] | | 0.45 | 0.24 | 0.02 |
| | rs10178921:rs355894 D'=1.0; $r^2$=0.22. enhancer@chr2:165635333-165635585 [rs355894] | 0.11 | 1 | 0.76 |
| | rs10178921:rs355863 D'=1.0; $r^2$=1.0. p10@COBLL1 [rs355863] | 0.1 | 1 | 0.77 |
| p@chr8:116588829..116588841,+ [rs2049867] | | 0.44 | 0.35 | 0.02 |
| p8@MMP9 [rs2236416] | | 0.43 | 0.55 | 0.03 |
| p@chr19:11334017..11334033,- [rs4804154, rs4804155] | | 0.43 | 0.55 | 0.03 |
| | rs4804154:rs737337 D'=0.92; $r^2$=0.66. p@chr19:11347228..11347241,- [rs737337];rs4804155:rs737337 D'=0.92; $r^2$=0.66. p@chr19:11347228..11347241,- [rs737337] | 0.35 | 1 | 0.17 |
| p@chr19:52327915..52327926,+ [rs3752125] | | 0.4 | 1 | 0.08 |
| chr11:122526685..122526694,+ [rs7127978] | | 0.39 | 1 | 0.09 |
| p@chr7:17287199..17287204,+ [rs6968554] | | 0.38 | 1 | 0.1 |
| p@chr15:58576429..58576468,- [rs1711037] | | 0.38 | 1 | 0.1 |
| p@chr12:20473724..20473755,+ [rs7134375] | | 0.38 | 1 | 0.1 |
| p@chr4:88057279..88057283,+ [rs1408] | | 0.37 | 1 | 0.14 |
| p5@INHBE [rs3809114] | | 0.36 | 1 | 0.14 |
| p3@LIPC [rs2070895] | | 0.36 | 1 | 0.15 |
| chr11:48066327..48066358,+ [rs7934659] | | 0.35 | 1 | 0.17 |
| enhancer@chr15:58884938-58885321 [rs11637365] | | 0.34 | 1 | 0.18 |
| p@chr19:8468345..8468362,+ [rs2230876] | | 0.33 | 1 | 0.2 |
| p@chr2:211540478..211540490,+ [rs1047891] | | 0.33 | 1 | 0.2 |
| p@chr16:68114139..68114174,+ [rs7188085] | | 0.32 | 1 | 0.23 |
| p@chr6:32412666..32412686,+ [rs7194] | | 0.32 | 1 | 0.23 |
| p@chr12:124433988..124434017,- [rs2178663] | | 0.32 | 1 | 0.23 |
| | rs2178663:rs9863 D'=1.0; $r^2$=0.92. p@chr12:124421545..124421559,- [rs9863] | 0.1 | 1 | 0.76 |
| p7@ZDHHC18 [rs12760759] | | 0.3 | 1 | 0.28 |
| | rs12760759:rs17162313 D'=1.0; $r^2$=1.0. enhancer@chr1:27175784-27176273 [rs17162313] | 0.19 | 1 | 0.57 |
| | rs12760759:rs1883660 D'=1.0; $r^2$=0.59. enhancer@chr1:27191386-27191927 [rs1883660] | 0.06 | 1 | 0.84 |
| p@chr11:126226533..126226546,- [rs73632737] | | 0.3 | 1 | 0.28 |
| chr15:63346713..63346752,+ [rs4468558] | | 0.29 | 1 | 0.29 |
| | rs4468558:rs7174174 D'=0.94; $r^2$=0.83. chr15:63372403..63372411,- [rs7174174] | 0.19 | 1 | 0.57 |
| p14@FADS1 [rs174548, rs174549, rs174550] | | 0.29 | 1 | 0.31 |
| p@chr16:56742451..56742454,- [rs4238789] | | 0.27 | 1 | 0.36 |
| p4@NISCH [rs4687618] | | 0.27 | 1 | 0.36 |
| | rs4687618:rs887515 D'=1.0; $r^2$=0.25. p33@NISCH [rs887515] | 0.1 | 1 | 0.76 |
| p3@TGOLN2 [rs10460587] | | 0.26 | 1 | 0.4 |
| enhancer@chr6:30796195-30796565 [rs1264350] | | 0.25 | 1 | 0.4 |
| p@chr19:54775040..54775082,- [rs419304] | | 0.25 | 1 | 0.4 |
| p@chr17:76377571..76377602,- [rs4082919] | | 0.25 | 1 | 0.42 |
| p@chr16:68299481..68299485,- [rs12596500] | | 0.24 | 1 | 0.42 |
| | rs12596500:rs12447119 D'=1.0; $r^2$=0.87. p5@SLC7A6 [rs12447119] | 0.18 | 1 | 0.57 |
| | rs12596500:rs7184821 D'=1.0; $r^2$=0.5. p2@ESRP2 [rs7184821] | 0.17 | 1 | 0.57 |
| p@chr16:67581701..67581719,- [rs13334205] | | 0.24 | 1 | 0.42 |
| chr10:5799591..5799598,+ [rs2275774] | | 0.24 | 1 | 0.42 |
| p@chr19:45242420..45242462,+ [rs1531517] | | 0.24 | 1 | 0.42 |
| p@chr4:103212519..103212530,- [rs233807] | | 0.24 | 1 | 0.43 |
| enhancer@chr1:230289705-230290142 [rs7551742] | | 0.23 | 1 | 0.46 |
| | rs7551742:rs4846905 D'=0.93; $r^2$=0.83. enhancer@chr1:230279464-230279771 [rs4846905] | 0.18 | 1 | 0.57 |
| p@chr17:37970009..37970013,- [rs9909593] | | 0.22 | 1 | 0.46 |
| p@chr4:157683775..157683778,- [rs1425486] | | 0.2 | 1 | 0.55 |
| p@chr11:45880009..45880015,+ [rs1401417] | | 0.2 | 1 | 0.57 |
| p@chr10:46076251..46076291,- [rs7086046] | | 0.19 | 1 | 0.57 |
| p@chr9:15298766..15298791,+ [rs675849] | | 0.18 | 1 | 0.57 |
| p2@CU690121 [rs12225230] | | 0.18 | 1 | 0.57 |
| p1@ENST00000438127 [rs3181] | | 0.18 | 1 | 0.57 |
| | rs3181:rs10892074 D'=1.0; $r^2$=0.17. p@chr11:116980715..116980720,- [rs10892074] | 0.14 | 1 | 0.64 |
| | rs3181:rs7930264 D'=1.0; $r^2$=0.89. enhancer@chr11:116870646-116870996 [rs7930264] | 0.07 | 1 | 0.83 |

| | | | | |
|---|---|---|---|---|
| enhancer@chr6:34663389-34663600 [rs3800457, rs3800458] | | 0.17 | 1 | 0.57 |
| | rs3800457:rs11757370 D'=1.0; $r^2$=0.3. enhancer@chr6:34760607-34760986 [rs11757370, rs11755420];rs3800457:rs11755420 D'=1.0; $r^2$=0.3. enhancer@chr6:34760607-34760986 [rs11757370, rs11755420];rs3800458:rs11757370 D'=1.0; $r^2$=0.89. enhancer@chr6:34760607-34760986 [rs11757370, rs11755420];rs3800458:rs11755420 D'=1.0; $r^2$=0.89. enhancer@chr6:34760607-34760986 [rs11757370, rs11755420] | 0.15 | 1 | 0.62 |
| | rs3800457:rs2764205 D'=1.0; $r^2$=0.96. p1@ENST00000408001 [rs2764205];rs3800458:rs2764205 D'=1.0; $r^2$=0.28. p1@ENST00000408001 [rs2764205] | 0.07 | 1 | 0.83 |
| p5@BAZ1B [rs1178979] | | 0.17 | 1 | 0.57 |
| p@chr8:126447258..126447275,+ [rs2385114] | | 0.17 | 1 | 0.57 |
| p@chr1:93627656..93627659,- [rs4847235] | | 0.17 | 1 | 0.57 |
| enhancer@chr10:65274637-65275000 [rs10761779] | | 0.16 | 1 | 0.59 |
| p2@APOM [rs3117583] | | 0.16 | 1 | 0.59 |
| | rs3117583:rs3130618 D'=1.0; $r^2$=1.0. p6@GPANK1 [rs3130618] | 0.13 | 1 | 0.68 |
| | rs3117583:rs3115663 D'=1.0; $r^2$=1.0. p5@PRRC2A [rs3115663] | 0.02 | 1 | 0.95 |
| p@chr20:33356385..33356390,- [rs2295353] | | 0.15 | 1 | 0.62 |
| p@chr11:117075245..117075293,- [rs508487] | | 0.14 | 1 | 0.66 |
| p@chr11:46934513..46934519,+ [rs79920646, rs7120113] | | 0.13 | 1 | 0.68 |
| p@chr6:139843931..139843972,+ [rs4896469] | | 0.13 | 1 | 0.7 |
| p4@TMEM110 [rs11720228] | | 0.12 | 1 | 0.72 |
| enhancer@chr15:58712788-58712944 [rs11854624] | | 0.1 | 1 | 0.76 |
| | rs11854624:rs17821310 D'=1.0; $r^2$=0.86. p1@LIPC [rs17821310] | 0.1 | 1 | 0.77 |
| p@chr10:65121474..65121480,- [rs12355784] | | 0.09 | 1 | 0.78 |
| enhancer@chr12:123387713-123388070 [rs10847980] | | 0.08 | 1 | 0.79 |
| p6@PSORS1C1 [rs1265099] | | 0.08 | 1 | 0.81 |
| | same SNP, different promoter: chr6:31105474..31105493,+ [rs1265099] | 0.01 | 1 | 0.98 |
| enhancer@chr6:127481688-127482073 [rs9491700] | | 0.06 | 1 | 0.84 |
| enhancer@chr2:227172292-227172683 [rs2713556] | | 0.06 | 1 | 0.84 |
| p7@RBM6 [rs6772095] | | 0.05 | 1 | 0.86 |
| enhancer@chr1:39736190-39736575 [rs4660603] | | 0.05 | 1 | 0.86 |
| p5@NOL3 [rs2233455] | | 0.03 | 1 | 0.93 |
| p@chr17:37405559..37405561,- [rs2061342] | | 0.03 | 1 | 0.93 |
| p@chr16:19871741..19871755,- [rs11865578] | | 0.03 | 1 | 0.93 |
| p3@PACSIN1 [rs11758326] | | 0.0 | 1 | 1.0 |

| Cell type | RRA p | FDR |
|---|---|---|
| Hepatocyte | 4.3e-14 | 0 |
| liver adult pool1 | 3.1e-10 | 0 |
| mesenchymalstemcells(adiposederived)adipogenicinduction day08 | 0.00014 | 0 |
| mesenchymalstemcells(adiposederived)adipogenicinduction day14 | 0.00018 | 0.0064 |
| Adipocyte differentiation day08 | 0.0037 | 0.0064 |
| Adipocyte subcutaneous | 0.0062 | 0.0064 |
| liver fetal pool1 | 0.0051 | 0.027 |
| mesenchymalstemcells(adiposederived)adipogenicinduction undifferentiatedcontrol | 0.028 | 0.042 |
| Adipocyte differentiation day12 | 0.028 | 0.042 |
| macLPS 00hr00min | 0.023 | 0.042 |
| macLPS 22hr | 0.045 | 0.049 |

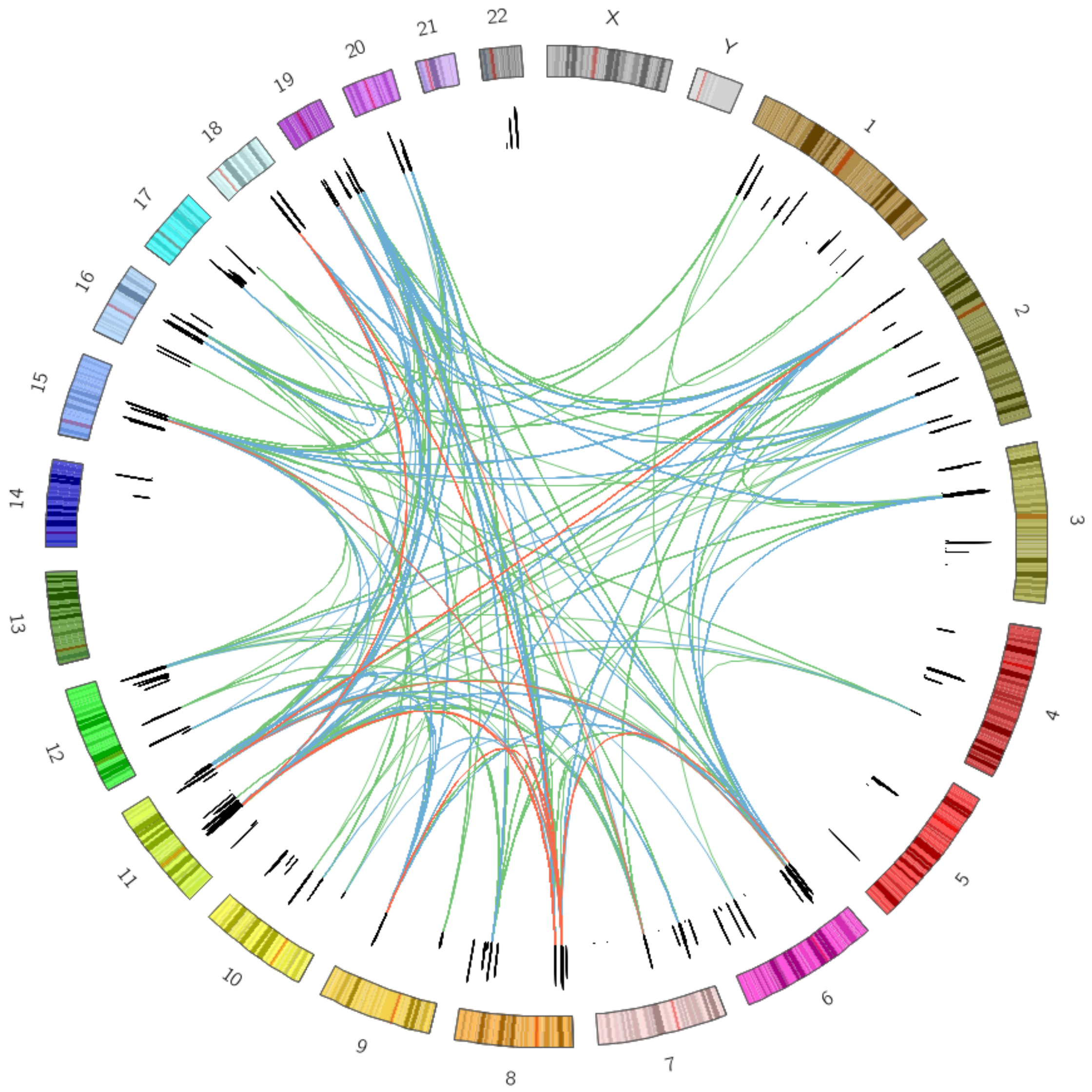


Figure 3: Circular plot of coexpression links between different locations on the genome (HDL Cholesterol). Colour indicates $log_{10}(p)$: red>3, blue> 2, green> 1.5). See main manuscript for full explanation.

# 4 LDL Cholesterol

| Top promoter[SNPs in top promoter] | Linkage | Corrected coexpression score | Bonferroni-corrected p-value | FDR |
|---|---|---|---|---|
| p94@APOB [rs1801701] | | 0.77 | 0 | 0.0 |
| p@chr17:64210704..64210723,+ [rs1801689] | | 0.73 | 0 | 0.0 |
| p3@ABCG5 [rs11887534] | | 0.66 | 0 | 0.0 |
| | rs11887534:rs13395550 D'=0.25; $r^2$=0.06. enhancer@chr2:44007303-44007575 [rs13395550] | 0.06 | 1 | 0.87 |
| p2@ENST00000518619 [rs9987289, rs4841132] | | 0.64 | 0 | 0.0 |
| | rs9987289:rs7357361 D'=1.0; $r^2$=0.57. enhancer@chr8:9219931-9220273 [rs7357361];rs4841132:rs7357361 D'=1.0; $r^2$=0.57. enhancer@chr8:9219931-9220273 [rs7357361] | 0.21 | 1 | 0.46 |
| p2@ASCC2 [rs4823054] | | 0.57 | 0 | 0.0 |
| p1@APOA5 [rs651821] | | 0.55 | 0 | 0.0 |
| p6@HNF1A-AS1 [rs1169286] | | 0.54 | 0 | 0.0 |
| | rs1169286:rs2258287 D'=0.68; $r^2$=0.39. p1@C12orf43 [rs2258287] | 0.18 | 1 | 0.55 |
| p@chr19:19616795..19616800,+ [rs1063966] | | 0.53 | 0 | 0.0 |
| | rs1063966:rs4808967 D'=1.0; $r^2$=1.0. p@chr19:19640454..19640461,+ [rs4808967] | 0.24 | 1 | 0.36 |
| p2@LPIN3 [rs6029636] | | 0.53 | 0.017 | 0.0 |
| p@chr16:72075048..72075055,- [rs9932951] | | 0.52 | 0.017 | 0.0 |
| | rs9932951:rs3213422 D'=0.29; $r^2$=0.08. p1@DHODH [rs34270657, rs3213422] | 0.43 | 0.52 | 0.03 |
| p2@NPC1L1 [rs41279633, rs17655652] | | 0.51 | 0.017 | 0.0 |
| | rs17655652:rs732797 D'=0.92; $r^2$=0.4. p@chr7:44646674..44646689,+ [rs732797] | 0.17 | 1 | 0.57 |
| p@chr1:109727753..109727759,+ [rs12239854] | | 0.49 | 0.051 | 0.0 |
| p1@SPC24 [rs892114, rs7251031] | | 0.47 | 0.17 | 0.01 |
| p1@FRK [rs1999929] | | 0.45 | 0.3 | 0.02 |
| p3@SLC22A1 [rs12208357] | | 0.43 | 0.64 | 0.04 |
| p4@HMGCR [rs3761740] | | 0.42 | 0.79 | 0.05 |
| p12@TRIB1 [rs17405319] | | 0.42 | 0.81 | 0.05 |
| p1@FEN1 [rs174538, rs412334] | | 0.41 | 0.9 | 0.05 |
| | rs174538:rs412334 D'=1.0; $r^2$=0.07. p4@C11orf10 [rs412334];same SNP, different promoter: p4@C11orf10 [rs412334] | 0.12 | 1 | 0.72 |
| p5@ABCA1 [rs1800978] | | 0.39 | 1 | 0.07 |
| p3@TIRAP [rs10893493] | | 0.37 | 1 | 0.1 |
| | rs10893493:rs638433 D'=0.96; $r^2$=0.92. enhancer@chr11:126180283-126180636 [rs638433] | 0.19 | 1 | 0.53 |
| p@chr1:63112277..63112278,+ [rs1168085] | | 0.37 | 1 | 0.1 |
| p@chr2:121310959..121310964,- [rs940688] | | 0.36 | 1 | 0.11 |
| p7@PCSK9 [rs11591147, rs11583680] | | 0.35 | 1 | 0.12 |
| p3@HAVCR1 [rs67960962] | | 0.35 | 1 | 0.12 |
| | rs67960962:rs6882076 D'=0.83; $r^2$=0.31. p1@TIMD4 [rs6882076] | 0.07 | 1 | 0.87 |
| p@chr9:136154265..136154271,- [rs579459, rs649129] | | 0.34 | 1 | 0.14 |
| chr19:19488822..19488846,+ [rs12973258] | | 0.33 | 1 | 0.16 |
| chr6:160770176..160770186,- [rs539298] | | 0.33 | 1 | 0.16 |
| p4@TAP2 [rs241448, rs241447, rs241452] | | 0.33 | 1 | 0.17 |
| p@chr19:45411878..45411884,- [rs7412] | | 0.31 | 1 | 0.2 |
| p3@C6orf10 [rs6913309] | | 0.31 | 1 | 0.21 |
| | rs6913309:rs13218331 D'=0.87; $r^2$=0.44. p@chr6:32411190..32411212,- [rs13218331] | 0.26 | 1 | 0.31 |
| p@chr8:145010972..145010996,- [rs6993938] | | 0.3 | 1 | 0.21 |
| p@chr19:45242420..45242462,+ [rs1531517] | | 0.3 | 1 | 0.21 |
| | rs1531517:rs10422182 D'=0.62; $r^2$=0.22. p@chr19:45159154..45159158,+ [rs10422182] | 0.09 | 1 | 0.82 |
| chr19:11031515..11031547,+ [rs2288842] | | 0.29 | 1 | 0.23 |
| | rs2288842:rs11085749 D'=1.0; $r^2$=0.61. p@chr19:10961151..10961183,+ [rs11085749] | 0.17 | 1 | 0.57 |
| p4@SH2B3 [rs739496] | | 0.29 | 1 | 0.23 |
| p@chr2:136594382..136594389,+ [rs2236783] | | 0.29 | 1 | 0.23 |
| p1@NAT2 [rs4646246] | | 0.29 | 1 | 0.24 |
| enhancer@chr20:34127589-34128004 [rs2104417] | | 0.27 | 1 | 0.31 |
| p5@RBM12 [rs6121015] | | 0.26 | 1 | 0.31 |
| p31@CORO1C [rs3741782] | | 0.26 | 1 | 0.31 |
| p23@USP1 [rs646179] | | 0.25 | 1 | 0.36 |
| p4@GSTM4 [rs1010167] | | 0.23 | 1 | 0.42 |
| p16@HLX [rs17597773] | | 0.23 | 1 | 0.42 |
| p@chr6:32658073..32658091,- [rs9469220] | | 0.22 | 1 | 0.44 |
| p12@PLCG1 [rs6124323] | | 0.21 | 1 | 0.48 |
| p@chr1:25756820..25756837,+ [rs9438904] | | 0.2 | 1 | 0.49 |
| p@chr6:31239406..31239455,+ [rs9264664] | | 0.2 | 1 | 0.51 |
| p1@POC5 [rs2291630] | | 0.19 | 1 | 0.53 |
| | rs2291630:rs2047059 D'=1.0; $r^2$=0.64. p2@POC5 [rs2047059] | 0.14 | 1 | 0.69 |
| | rs2291630:rs7715806 D'=0.95; $r^2$=0.39. enhancer@chr5:75034241-75034569 [rs7715806] | 0.1 | 1 | 0.76 |
| | rs2291630:rs34358 D'=1.0; $r^2$=0.47. p@chr5:74965139..74965165,+ [rs34358] | 0.08 | 1 | 0.82 |
| p1@SLC44A4 [rs605203] | | 0.19 | 1 | 0.53 |
| chr20:39634184..39634189,- [rs1000410] | | 0.17 | 1 | 0.57 |
| enhancer@chr16:56985400-56985562 [rs72786786, rs12448528] | | 0.17 | 1 | 0.57 |
| p@chr1:63266602..63266609,- [rs11208033] | | 0.17 | 1 | 0.57 |
| chr19:10742301..10742328,+ [rs2288904, rs1560711] | | 0.15 | 1 | 0.63 |
| p@chr12:112212004..112212016,+ [rs2238151] | | 0.15 | 1 | 0.64 |
| p@chr2:216286925..216286942,- [rs1250248] | | 0.13 | 1 | 0.7 |
| enhancer@chr14:94813306-94813452 [rs926144] | | 0.13 | 1 | 0.7 |
| p4@FUT1 [rs838136] | | 0.13 | 1 | 0.7 |
| p3@SPTY2D1 [rs7943121] | | 0.12 | 1 | 0.7 |
| enhancer@chr5:74805918-74806308 [rs5744533] | | 0.11 | 1 | 0.76 |
| p@chr10:113928643..113928689,- [rs2803616] | | 0.11 | 1 | 0.76 |
| p1@ENST00000364447 [rs1003116] | | 0.1 | 1 | 0.78 |
| enhancer@chr19:46018095-46018753 [rs7255743] | | 0.08 | 1 | 0.82 |
| enhancer@chr2:136816831-136817220 [rs749873] | | 0.08 | 1 | 0.85 |
| p@chr3:12704990..12705013,- [rs904453] | | 0.07 | 1 | 0.85 |

| | | | | |
|---|---|---|---|---|
| p@chr11:117075245..117075293,- [rs508487] | | 0.06 | 1 | 0.87 |
| enhancer@chr1:109754796-109754995 [rs187413240] | | 0.06 | 1 | 0.87 |
| enhancer@chr11:61713558-61713889 [rs1534842] | | 0.06 | 1 | 0.87 |
| p@chr6:16131425..16131449,+ [rs2235215, rs2066905] | | 0.06 | 1 | 0.87 |
| enhancer@chr8:59311673-59312115 [rs1030431] | | 0.05 | 1 | 0.88 |
| p@chr2:135893438..135893485,+ [rs10445686] | | 0.04 | 1 | 0.92 |
| enhancer@chr6:34663389-34663600 [rs3800457] | | 0.04 | 1 | 0.92 |
| enhancer@chr1:92668364-92668570 [rs3131816] | | 0.03 | 1 | 0.92 |
| p3@C17orf57 [rs2271803] | | 0.03 | 1 | 0.92 |
| p@chr5:74964235..74964256,+ [rs7715739, rs369034591] | | 0.02 | 1 | 0.96 |
| p1@ATXN2 [rs695871] | | 0.02 | 1 | 0.96 |
| p@chr6:32078154..32078167,- [rs3807039] | | 0.02 | 1 | 0.96 |
| enhancer@chr1:25802039-25802147 [rs66844552] | | 0.01 | 1 | 0.96 |
| p@chr6:31512638..31512648,- [rs2071593] | | 0.0 | 1 | 1.0 |

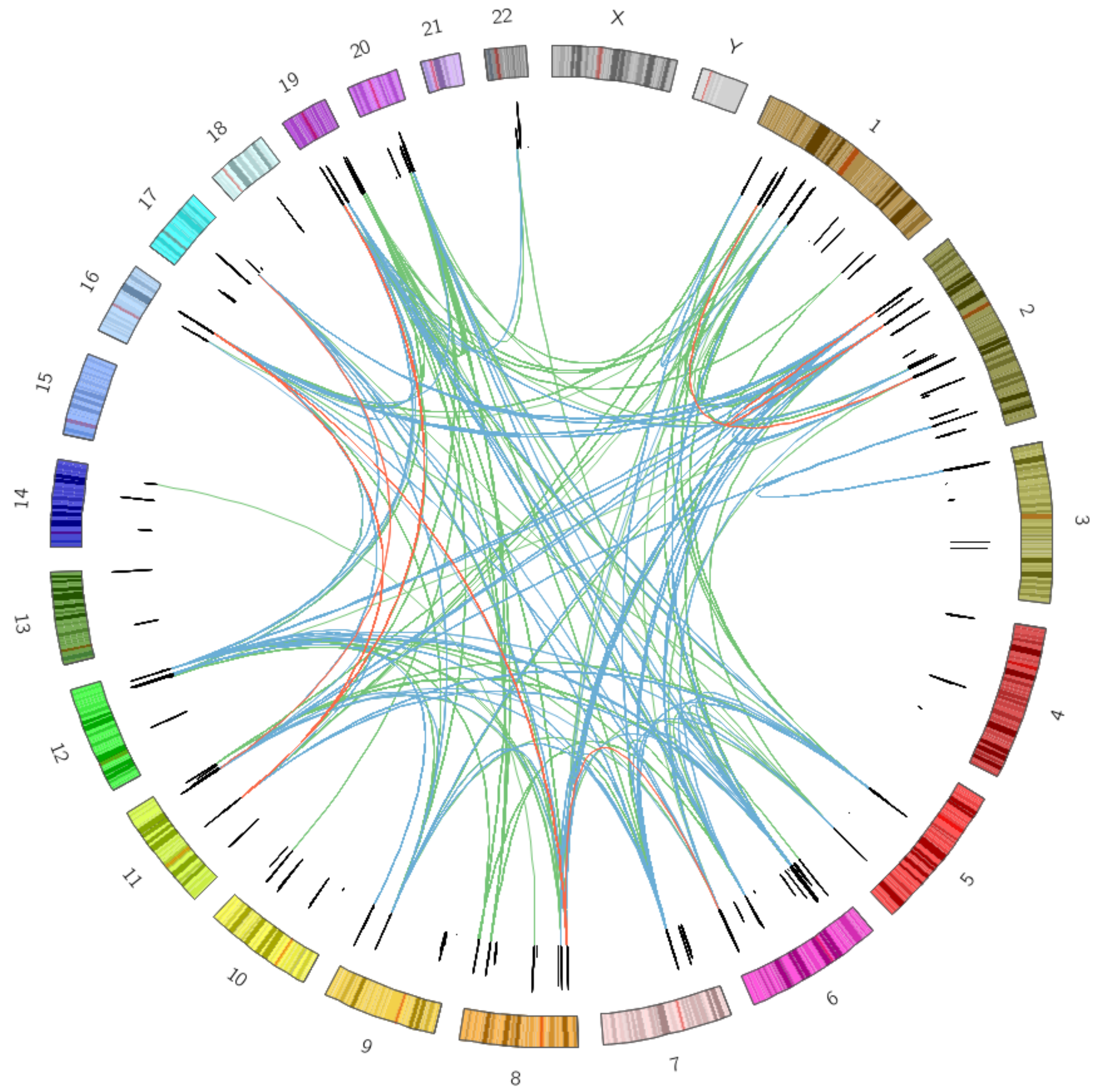


Figure 4: Circular plot of coexpression links between different locations on the genome (LDL Cholesterol). Colour indicates $log_{10}(p)$: red>3, blue> 2, green> 1.5). See main manuscript for full explanation.

# 5 Total Cholesterol

| Top promoter[SNPs in top promoter] | Linkage | Corrected coexpression score | Bonferroni-corrected p-value | FDR |
|---|---|---|---|---|
| p58@APOB [rs533617] | | 0.74 | 0 | 0.0 |
| p@chr7:44581054..44581057,+ [rs41279633, rs17655652] | | 0.68 | 0 | 0.0 |
| | rs17655652:rs732797 D'=0.92; $r^2$=0.4. p@chr7:44646674..44646689,+ [rs732797] | 0.26 | 1 | 0.3 |
| p6@HNF1A-AS1 [rs1169286] | | 0.65 | 0 | 0.0 |
| | rs1169286:rs2258287 D'=0.68; $r^2$=0.39. p1@C12orf43 [rs2258287] | 0.16 | 1 | 0.61 |
| p2@ENST00000518619 [rs11774381, rs9987289, rs4841132] | | 0.64 | 0 | 0.0 |
| | rs9987289:rs7357361 D'=1.0; $r^2$=0.57. enhancer@chr8:9219931-9220273 [rs7357361];rs4841132:rs7357361 D'=1.0; $r^2$=0.57. enhancer@chr8:9219931-9220273 [rs7357361] | 0.26 | 1 | 0.3 |
| p3@LIPC [rs2070895] | | 0.61 | 0 | 0.0 |
| p5@ABCA1 [rs1800978] | | 0.58 | 0 | 0.0 |
| p3@ABCG5 [rs11887534] | | 0.54 | 0 | 0.0 |
| | rs11887534:rs13395550 D'=0.25; $r^2$=0.06. enhancer@chr2:44007303-44007575 [rs13395550] | 0.08 | 1 | 0.81 |
| p1@GPN1 [rs3749147] | | 0.54 | 0 | 0.0 |
| | same SNP, different promoter: p1@CCDC121 [rs3749147] | 0.06 | 1 | 0.87 |
| enhancer@chr6:30731311-30731508 [rs3095338] | | 0.52 | 0 | 0.0 |
| | rs3095338:rs8233 D'=0.91; $r^2$=0.62. p@chr6:30692851..30692934,+ [rs8233] | 0.17 | 1 | 0.58 |
| p3@HAVCR1 [rs67960962] | | 0.51 | 0 | 0.0 |
| | rs67960962:rs6882076 D'=0.83; $r^2$=0.31. p1@TIMD4 [rs6882076] | 0.06 | 1 | 0.87 |
| p1@APOA5 [rs651821] | | 0.51 | 0 | 0.0 |
| | rs651821:rs689243 D'=1.0; $r^2$=0.21. enhancer@chr11:116722234-116722744 [rs689243] | 0.0 | 1 | 1.0 |
| p1@ENST00000447070 [rs6760828] | | 0.5 | 0 | 0.0 |
| | rs6760828:rs4665963 D'=0.77; $r^2$=0.31. p@chr2:27528919..27528940,+ [rs4665963] | 0.33 | 1 | 0.12 |
| p@chr16:72075048..72075055,- [rs9932951] | | 0.48 | 0.017 | 0.0 |
| | rs9932951:rs3213422 D'=0.29; $r^2$=0.08. p1@DHODH [rs34270657, rs3213422] | 0.43 | 0.17 | 0.01 |
| p1@SPTY2D1 [rs7943121] | | 0.48 | 0.017 | 0.0 |
| p@chr19:19616795..19616800,+ [rs1063966] | | 0.48 | 0.017 | 0.0 |
| | rs1063966:rs73004933 D'=1.0; $r^2$=0.19. p@chr19:19675738..19675742,- [rs73004933] | 0.37 | 1 | 0.06 |
| | rs1063966:rs4808967 D'=1.0; $r^2$=1.0. p@chr19:19640454..19640461,+ [rs4808967] | 0.11 | 1 | 0.76 |
| p@chr6:26500524..26500540,+ [rs13194984] | | 0.47 | 0.017 | 0.0 |
| p3@SLC22A1 [rs12208357] | | 0.46 | 0.067 | 0.0 |
| p6@RBM39 [rs2425090] | | 0.46 | 0.067 | 0.0 |
| p16@HLA-DQA1 [rs9272775] | | 0.44 | 0.13 | 0.01 |
| p37@CFB [rs2072633] | | 0.44 | 0.13 | 0.01 |
| p@chr2:27975692..27975750,+ [rs6547796] | | 0.43 | 0.17 | 0.01 |
| | rs6547796:rs13013484 D'=1.0; $r^2$=0.9. p@chr2:27988549..27988557,- [rs13013484] | 0.17 | 1 | 0.58 |
| chr6:160770176..160770186,- [rs474513, rs539298] | | 0.43 | 0.22 | 0.01 |
| p7@PCSK9 [rs11591147] | | 0.42 | 0.37 | 0.02 |
| p@chr19:45411878..45411884,- [rs7412] | | 0.41 | 0.57 | 0.02 |
| p1@FEN1 [rs412334, rs174538] | | 0.4 | 0.86 | 0.03 |
| | same SNP, different promoter: p4@C11orf10 [rs412334];rs174538:rs412334 D'=1.0; $r^2$=0.07. p4@C11orf10 [rs412334] | 0.35 | 1 | 0.07 |
| p1@SPATC1 [rs6985603] | | 0.39 | 1 | 0.04 |
| p@chr18:47176875..47176889,+ [rs4939886, rs4939887, rs4939888] | | 0.39 | 1 | 0.04 |
| p1@NAT2 [rs4646246] | | 0.38 | 1 | 0.05 |
| p@chr9:136154265..136154271,- [rs579459, rs649129] | | 0.38 | 1 | 0.05 |
| | rs579459:rs568203 D'=1.0; $r^2$=0.1. p@chr9:136151396..136151407,- [rs568203];rs649129:rs568203 D'=1.0; $r^2$=0.1. p@chr9:136151396..136151407,- [rs568203] | 0.26 | 1 | 0.3 |
| enhancer@chr8:126446642-126447323 [rs2385114] | | 0.37 | 1 | 0.06 |
| p@chr1:63112277..63112278,+ [rs1168085] | | 0.37 | 1 | 0.06 |
| p3@NSMAF [rs2279460] | | 0.37 | 1 | 0.06 |
| enhancer@chr20:34127589-34128004 [rs2104417] | | 0.36 | 1 | 0.07 |
| p13@UGT1A6 [rs2070959] | | 0.36 | 1 | 0.07 |
| | rs2070959:rs17864701 D'=0.92; $r^2$=0.8. p2@DNAJB3 [rs17864701] | 0.25 | 1 | 0.3 |
| p2@SPC24 [rs892114, rs7251031] | | 0.36 | 1 | 0.07 |
| enhancer@chr5:74805918-74806308 [rs5744533] | | 0.36 | 1 | 0.07 |
| p1@AK311221 [rs9469089] | | 0.35 | 1 | 0.09 |
| | rs9469089:rs3134931 D'=0.49; $r^2$=0.14. p@chr6:32190527..32190536,- [rs3134931] | 0.09 | 1 | 0.81 |
| p@chr16:68114139..68114174,+ [rs7188085] | | 0.34 | 1 | 0.1 |
| p@chr1:221054751..221054771,- [rs17597773] | | 0.34 | 1 | 0.1 |
| p1@POC5 [rs2291630] | | 0.33 | 1 | 0.11 |
| | rs2291630:rs34358 D'=1.0; $r^2$=0.47. p@chr5:74965139..74965165,+ [rs34358] | 0.24 | 1 | 0.37 |
| | rs2291630:rs2047059 D'=1.0; $r^2$=0.64. p2@POC5 [rs2047059] | 0.17 | 1 | 0.58 |
| | rs2291630:rs7715806 D'=0.95; $r^2$=0.39. enhancer@chr5:75034241-75034569 [rs7715806] | 0.16 | 1 | 0.63 |
| p2@LY6G6C [rs805292] | | 0.33 | 1 | 0.11 |
| p@chr6:16131425..16131449,+ [rs2235215] | | 0.33 | 1 | 0.11 |
| p@chr2:136594310..136594317,+ [rs2236783] | | 0.33 | 1 | 0.12 |
| | rs2236783:rs1438307 D'=0.94; $r^2$=0.85. p2@UBXN4 [rs1438307] | 0.13 | 1 | 0.74 |
| enhancer@chr6:135435494-135435847 [rs9483788] | | 0.32 | 1 | 0.13 |
| p1@HLA-DRB9 [rs13191565] | | 0.31 | 1 | 0.17 |
| p1@USP1 [rs646179] | | 0.3 | 1 | 0.2 |
| enhancer@chr19:45242034-45242469 [rs1531517] | | 0.28 | 1 | 0.23 |
| | rs1531517:rs10422182 D'=0.62; $r^2$=0.22. p@chr19:45159154..45159158,+ [rs10422182] | 0.09 | 1 | 0.81 |

| | | | | |
|---|---|---|---|---|
| p@chr19:10961151..10961183,+ [rs11085749] | | 0.28 | 1 | 0.25 |
| | rs11085749:rs2288842 D'=1.0; $r^2$=0.61. chr19:11031515..11031547,+ [rs2288842] | 0.23 | 1 | 0.38 |
| p12@PLCG1 [rs6124323] | | 0.28 | 1 | 0.25 |
| enhancer@chr10:17254796-17255338 [rs7080366] | | 0.27 | 1 | 0.26 |
| p2@LPIN3 [rs6029636] | | 0.26 | 1 | 0.29 |
| p1@ENST00000408001 [rs2764205] | | 0.25 | 1 | 0.3 |
| | rs2764205:rs3800457 D'=1.0; $r^2$=0.96. enhancer@chr6:34663389-34663600 [rs3800457] | 0.08 | 1 | 0.81 |
| enhancer@chr16:56985400-56985562 [rs12448528, rs72786786] | | 0.25 | 1 | 0.3 |
| p@chr9:15298766..15298791,+ [rs675849] | | 0.24 | 1 | 0.36 |
| p@chr1:63266602..63266609,- [rs11208033, rs10493328, rs4409690] | | 0.23 | 1 | 0.38 |
| | rs11208033:rs12122434 D'=1.0; $r^2$=0.22. p@chr1:63261897..63261900,+ [rs12122434];rs10493328:rs12122434 D'=1.0; $r^2$=0.22. p@chr1:63261897..63261900,+ [rs12122434];rs4409690:rs12122434 D'=1.0; $r^2$=0.22. p@chr1:63261897..63261900,+ [rs12122434] | 0.08 | 1 | 0.81 |
| p4@SH2B3 [rs739496] | | 0.23 | 1 | 0.38 |
| p3@C6orf10 [rs6913309, rs2050189] | | 0.21 | 1 | 0.45 |
| p@chr2:113841162..113841225,+ [rs6734238] | | 0.21 | 1 | 0.45 |
| p1@FRK [rs1999929] | | 0.21 | 1 | 0.45 |
| p@chr16:71934252..71934256,+ [rs4788576] | | 0.2 | 1 | 0.48 |
| enhancer@chr2:136816831-136817220 [rs749873] | | 0.19 | 1 | 0.55 |
| | rs749873:rs309137 D'=0.86; $r^2$=0.52. enhancer@chr2:136765824-136766130 [rs309137] | 0.12 | 1 | 0.76 |
| | rs749873:rs6714750 D'=1.0; $r^2$=0.55. enhancer@chr2:136783030-136783335 [rs6714750] | 0.1 | 1 | 0.79 |
| p4@HMGCR [rs3761740] | | 0.19 | 1 | 0.56 |
| enhancer@chr19:46018095-46018753 [rs7255743] | | 0.18 | 1 | 0.56 |
| p6@UBASH3B [rs7127978] | | 0.18 | 1 | 0.58 |
| enhancer@chr11:48004294-48004631 [rs7946766] | | 0.17 | 1 | 0.58 |
| chr20:39634184..39634189,- [rs1000410] | | 0.17 | 1 | 0.58 |
| p@chr2:203131655..203131667,- [rs7581542] | | 0.17 | 1 | 0.58 |
| chr11:118486256..118486298,+ [rs11603023] | | 0.17 | 1 | 0.58 |
| p@chr11:116980715..116980720,- [rs10892074] | | 0.15 | 1 | 0.66 |
| | rs10892074:rs508487 D'=1.0; $r^2$=0.09. p@chr11:117075245..117075293,- [rs508487] | 0.02 | 1 | 0.96 |
| enhancer@chr16:31129616-31130225 [rs9925964] | | 0.15 | 1 | 0.66 |
| p@chr12:112212004..112212016,+ [rs2238151] | | 0.14 | 1 | 0.67 |
| p4@GSTM4 [rs1010167] | | 0.14 | 1 | 0.67 |
| p@chr17:76377571..76377602,- [rs4082919] | | 0.13 | 1 | 0.74 |
| p@chr1:109784420..109784424,+ [rs688386] | | 0.12 | 1 | 0.75 |
| p@chr4:88057279..88057283,+ [rs1408] | | 0.12 | 1 | 0.76 |
| p6@SYN2 [rs3773364] | | 0.11 | 1 | 0.78 |
| chr19:10742301..10742328,+ [rs2288904, rs1560711] | | 0.11 | 1 | 0.78 |
| chr6:31808499..31808514,+ [rs599707] | | 0.1 | 1 | 0.78 |
| p1@ERGIC2 [rs2278093] | | 0.1 | 1 | 0.8 |
| p2@HLA-L [rs9391806] | | 0.09 | 1 | 0.81 |
| p6@PHC1 [rs3809218] | | 0.09 | 1 | 0.81 |
| p@chr5:74964235..74964256,+ [rs7715739, rs369034591] | | 0.09 | 1 | 0.81 |
| p@chr17:37405559..37405561,- [rs2061342] | | 0.08 | 1 | 0.81 |
| p@chr2:28124429..28124474,+ [rs10177845] | | 0.08 | 1 | 0.81 |
| enhancer@chr22:35708559-35708961 [rs138774] | | 0.08 | 1 | 0.82 |
| p@chr2:216286896..216286912,- [rs1250248] | | 0.07 | 1 | 0.87 |
| p@chr2:135893438..135893485,+ [rs10445686] | | 0.06 | 1 | 0.87 |
| enhancer@chr17:37836315-37836462 [rs9303274] | | 0.06 | 1 | 0.87 |
| | rs9303274:rs1053651 D'=0.91; $r^2$=0.65. p@chr17:37822585..37822591,+ [rs1053651] | 0.01 | 1 | 0.99 |
| enhancer@chr15:58712788-58712944 [rs11854624] | | 0.06 | 1 | 0.87 |
| | rs11854624:rs17821310 D'=1.0; $r^2$=0.86. p1@LIPC [rs17821310] | 0.01 | 1 | 0.99 |
| enhancer@chr8:59311673-59312115 [rs1030431] | | 0.05 | 1 | 0.9 |
| p@chr20:34001017..34001019,- [rs6087704] | | 0.05 | 1 | 0.9 |
| enhancer@chr11:61713558-61713889 [rs1534842] | | 0.04 | 1 | 0.91 |
| p1@GPR61 [rs552101] | | 0.04 | 1 | 0.91 |
| p@chr3:12704956..12704973,- [rs904453] | | 0.04 | 1 | 0.92 |
| p2@CU690121 [rs12225230] | | 0.04 | 1 | 0.92 |
| enhancer@chr1:92668364-92668570 [rs3131816] | | 0.03 | 1 | 0.96 |
| p2@PACSIN1 [rs11758326] | | 0.01 | 1 | 0.98 |
| p1@ATXN2 [rs695871] | | 0.0 | 1 | 1.0 |

| Cell type | RRA p | FDR |
|---|---|---|
| Hepatocyte | 1.6e-17 | 0 |
| liver adult pool1 | 6.8e-12 | 0 |
| Prostate Epithelial Cells | 7.6e-07 | 0 |
| liver fetal pool1 | 4.8e-06 | 0 |
| intestinal epithelial cells polarised | 0.00013 | 0 |
| colon adult pool1 | 0.0042 | 0.0029 |
| HES3GFP Embryonic Stem cells cardiomyocytic induction day12 | 4.1e-06 | 0.0037 |
| HES3GFP Embryonic Stem cells cardiomyocytic induction day05 | 0.00097 | 0.0038 |
| Renal Proximal Tubular Epithelial Cell | 0.041 | 0.0038 |
| Renal Mesangial Cells | 0.024 | 0.0043 |
| H9 Embryoid body cells melanocytic induction day12 | 0.0016 | 0.0047 |
| kidney fetal pool1 | 0.019 | 0.0072 |
| HES3GFP Embryonic Stem cells cardiomyocytic induction day02 | 0.0019 | 0.011 |
| HES3GFP Embryonic Stem cells cardiomyocytic induction day08 | 0.0057 | 0.038 |
| HES3GFP Embryonic Stem cells cardiomyocytic induction day07 | 0.0023 | 0.04 |

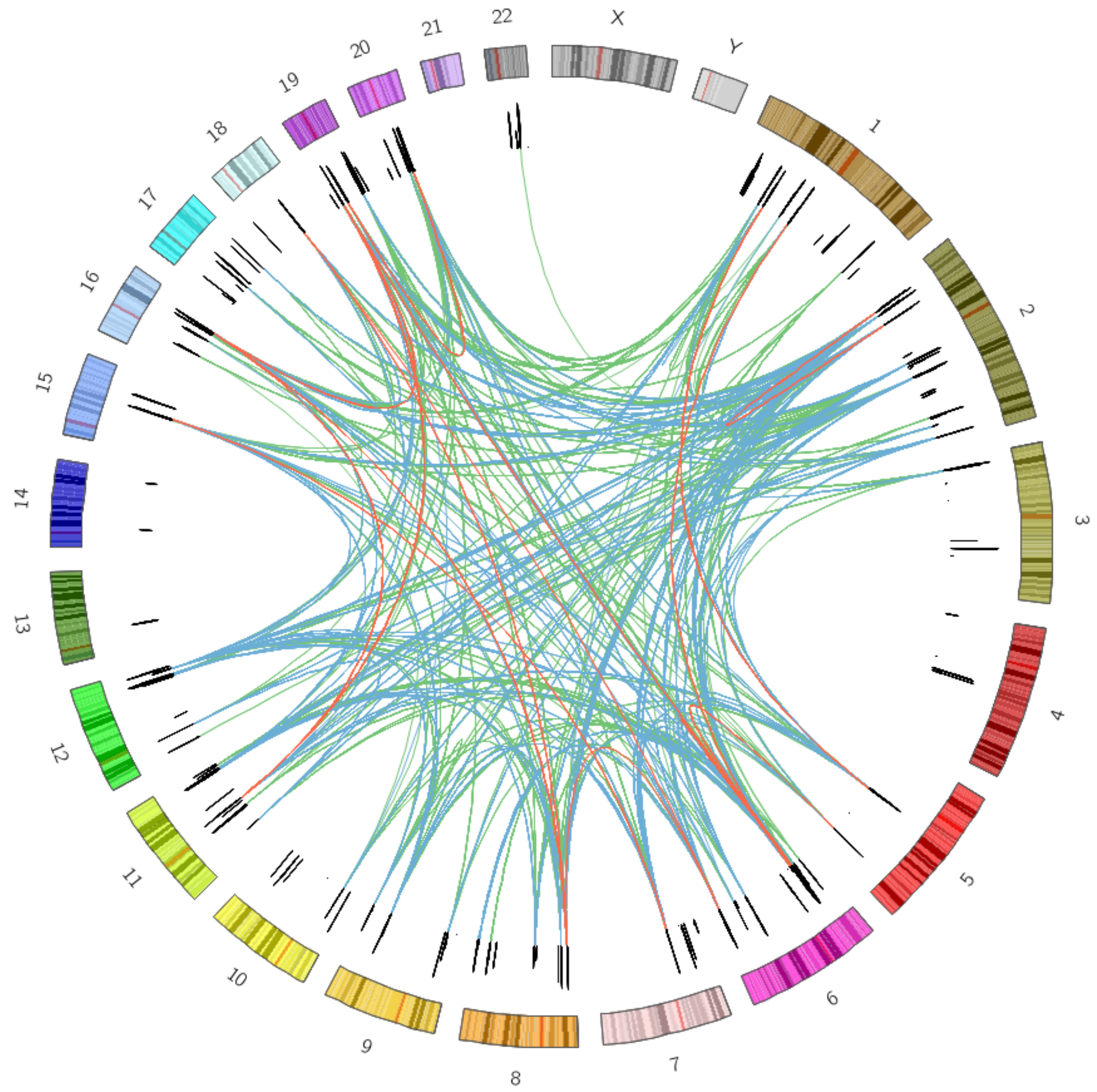


Figure 5: Circular plot of coexpression links between different locations on the genome (Total Cholesterol). Colour indicates $log_{10}(p)$: red>3, blue> 2, green> 1.5). See main manuscript for full explanation.

# 6 Triglycerides

| Top promoter[SNPs in top promoter] | Linkage | Corrected coexpression score | Bonferroni-corrected p-value | FDR |
|---|---|---|---|---|
| p67@APOB [rs676210] | | 0.63 | 0 | 0.0 |
| p@chr2:165603346..165603357,- [rs10178921] | | 0.56 | 0.018 | 0.01 |
| p@chr2:27528919..27528940,+ [rs4665963] | | 0.53 | 0.036 | 0.01 |
| | rs4665963:rs11684134 D'=0.8; $r^2$=0.3. p1@LOC100505624 [rs11684134] | 0.42 | 0.29 | 0.02 |
| p@chr7:73106975..73106980,+ [rs2293490] | | 0.52 | 0.036 | 0.01 |
| p32@ACSL5 [rs11195943] | | 0.51 | 0.036 | 0.01 |
| p@chr11:116691448..116691464,- [rs5110, rs675] | | 0.5 | 0.036 | 0.01 |
| p@chr19:45411878..45411884,- [rs7412] | | 0.5 | 0.054 | 0.01 |
| p1@ZSWIM1 [rs3746497] | | 0.5 | 0.054 | 0.01 |
| p@chr7:17287199..17287204,+ [rs6968554] | | 0.49 | 0.054 | 0.01 |
| p@chr6:31254912..31254928,- [rs9468920] | | 0.48 | 0.054 | 0.01 |
| enhancer@chr7:72939749-72940060 [rs80189144] | | 0.48 | 0.072 | 0.01 |
| p@chr12:124433988..124434017,- [rs2178663] | | 0.46 | 0.11 | 0.01 |
| p1@BAG6 [rs3117582] | | 0.45 | 0.13 | 0.01 |
| p@chr6:52617751..52617757,- [rs2180314] | | 0.44 | 0.14 | 0.01 |
| p@chr19:7184803..7184814,- [rs17253937] | | 0.43 | 0.24 | 0.02 |
| p1@ENST00000518619 [rs17149760] | | 0.42 | 0.25 | 0.02 |
| p@chr20:44640866..44640875,- [rs3918256] | | 0.4 | 0.47 | 0.03 |
| p@chr4:88057279..88057283,+ [rs1408] | | 0.4 | 0.51 | 0.03 |
| | rs1408:rs3755980 D'=1.0; $r^2$=0.14. p@chr4:88042092..88042099,- [rs3755980] | 0.18 | 1 | 0.45 |
| p1@ENST00000448198, p1@ENST00000455328 [rs3916765] | | 0.38 | 0.98 | 0.05 |
| enhancer@chr19:19483893-19484092 [rs59148799] | | 0.37 | 1 | 0.05 |
| p1@NSUN5 [rs1880948] | | 0.37 | 1 | 0.05 |
| chr6:30798448..30798455,- [rs1264347] | | 0.37 | 1 | 0.05 |
| | rs1264347:rs886422 D'=1.0; $r^2$=1.0. p23@DDR1 [rs886422] | 0.05 | 1 | 0.82 |
| p2@LIPC [rs2070895] | | 0.37 | 1 | 0.05 |
| p@chr19:45487536..45487571,+ [rs16979600] | | 0.36 | 1 | 0.06 |
| | rs16979600:rs3760629 D'=1.0; $r^2$=0.05. p3@CLPTM1 [rs3760629] | 0.13 | 1 | 0.61 |
| p2@LY6G6C [rs805292] | | 0.36 | 1 | 0.06 |
| p@chr4:87873088..87873092,+ [rs1992876] | | 0.35 | 1 | 0.07 |
| p@chr8:19782798..19782817,- [rs28522139] | | 0.34 | 1 | 0.08 |
| p@chr8:11396776..11396795,+ [rs2409784] | | 0.33 | 1 | 0.09 |
| chr2:28203328..28203338,+ [rs898031] | | 0.33 | 1 | 0.09 |
| | rs898031:rs2305929 D'=1.0; $r^2$=0.1. p@chr2:28114209..28114224,+ [rs2305929] | 0.1 | 1 | 0.68 |
| p1@HAX1 [rs11556341] | | 0.33 | 1 | 0.11 |
| | same SNP, different promoter: p@chr1:154244875..154244901,- [rs11556341] | 0.14 | 1 | 0.61 |
| p@chr2:28634997..28635008,+ [rs12624279] | | 0.32 | 1 | 0.11 |
| p1@ZNF646 [rs2303222] | | 0.32 | 1 | 0.11 |
| p2@F2 [rs2070852] | | 0.3 | 1 | 0.15 |
| | rs2070852:rs7932354 D'=1.0; $r^2$=1.0. p1@ARHGAP1 [rs7932354] | 0.13 | 1 | 0.61 |
| enhancer@chr15:58712788-58712944 [rs11854624] | | 0.3 | 1 | 0.16 |
| | rs11854624:rs17821310 D'=1.0; $r^2$=0.86. p1@LIPC [rs17821310] | 0.1 | 1 | 0.68 |
| p1@ENST00000519197 [rs28597716] | | 0.29 | 1 | 0.18 |
| enhancer@chr11:47109490-47109615 [rs4319472] | | 0.28 | 1 | 0.2 |
| p1@NAT2 [rs4646246] | | 0.28 | 1 | 0.21 |
| enhancer@chr16:56985400-56985562 [rs72786786, rs12448528] | | 0.27 | 1 | 0.23 |
| p@chr6:33153425..33153432,- [rs9277934] | | 0.27 | 1 | 0.23 |
| | rs9277934:rs2076311 D'=1.0; $r^2$=1.0. p@chr6:33145331..33145378,- [rs2076311] | 0.07 | 1 | 0.79 |
| enhancer@chr8:11032163-11032532 [rs6986032] | | 0.27 | 1 | 0.23 |
| p@chr1:62928606..62928608,+ [rs998403] | | 0.27 | 1 | 0.23 |
| p@chr1:63112277..63112278,+ [rs1168085] | | 0.26 | 1 | 0.23 |
| p@chr6:32411003..32411021,- [rs3135391] | | 0.26 | 1 | 0.25 |
| p11@SIDT2 [rs474339] | | 0.25 | 1 | 0.26 |
| | rs474339:rs10892074 D'=1.0; $r^2$=0.25. p@chr11:116980715..116980720,- [rs10892074] | 0.14 | 1 | 0.61 |
| | rs474339:rs508487 D'=1.0; $r^2$=0.37. p@chr11:117075245..117075293,- [rs508487] | 0.05 | 1 | 0.82 |
| p@chr19:19640431..19640450,+ [rs4808967] | | 0.24 | 1 | 0.3 |
| | rs4808967:rs1063966 D'=1.0; $r^2$=1.0. p@chr19:19616795..19616800,+ [rs1063966] | 0.17 | 1 | 0.48 |
| p@chr8:19830089..19830095,+ [rs1569209] | | 0.24 | 1 | 0.31 |
| p1@TIMD4 [rs6882076] | | 0.23 | 1 | 0.31 |
| enhancer@chr11:116722234-116722744 [rs689243] | | 0.23 | 1 | 0.32 |
| p@chr11:47303410..47303414,+ [rs326217] | | 0.22 | 1 | 0.34 |
| p@chr8:126442070..126442090,- [rs4871594] | | 0.21 | 1 | 0.37 |
| p@chr2:27975692..27975750,+ [rs6547796] | | 0.21 | 1 | 0.37 |
| p@chr1:63266602..63266609,- [rs11208033, rs10493328, rs4409690] | | 0.21 | 1 | 0.37 |
| | rs11208033:rs12122434 D'=1.0; $r^2$=0.22. p@chr1:63261897..63261900,+ [rs12122434];rs10493328:rs12122434 D'=1.0; $r^2$=0.22. p@chr1:63261897..63261900,+ [rs12122434];rs4409690:rs12122434 D'=1.0; $r^2$=0.22. p@chr1:63261897..63261900,+ [rs12122434] | 0.19 | 1 | 0.42 |
| p3@FADS2 [rs968567] | | 0.2 | 1 | 0.42 |
| chr6:31808499..31808514,+ [rs599707] | | 0.19 | 1 | 0.42 |
| enhancer@chr10:65274637-65275000 [rs10761779] | | 0.19 | 1 | 0.45 |
| chr6:160770176..160770186,- [rs474513, rs539298] | | 0.18 | 1 | 0.48 |
| enhancer@chr6:32913033-32913520 [rs1480380] | | 0.16 | 1 | 0.52 |
| enhancer@chr2:227020272-227020467 [rs13404263] | | 0.16 | 1 | 0.52 |
| p1@HLA-DQB1 [rs3891175] | | 0.15 | 1 | 0.56 |
| enhancer@chr3:52531917-52532418 [rs13326165] | | 0.15 | 1 | 0.59 |
| p9@IFT172 [rs780104, rs79740025, rs6734392] | | 0.13 | 1 | 0.61 |
| p@chr10:65121474..65121480,- [rs12355784] | | 0.13 | 1 | 0.61 |
| enhancer@chr1:230279464-230279771 [rs4846905] | | 0.13 | 1 | 0.61 |

| | | | | |
|---|---|---|---|---|
| | rs4846905:rs7551742 D'=0.93; $r^2$=0.83. enhancer@chr1:230289705-230290142 [rs7551742] | 0.08 | 1 | 0.77 |
| enhancer@chr2:28319174-28319559 [rs7568903] | | 0.13 | 1 | 0.61 |
| chr6:32148013..32148032,+ [rs3134943] | | 0.13 | 1 | 0.61 |
| | rs3134943:rs3134931 D'=1.0; $r^2$=0.08. p@chr6:32190527..32190536,- [rs3134931] | 0.07 | 1 | 0.79 |
| | rs3134943:rs3131283 D'=1.0; $r^2$=0.92. p3@PRRT1 [rs3131283] | 0.03 | 1 | 0.88 |
| p3@CPEB4 [rs55946741] | | 0.11 | 1 | 0.67 |
| p1@ENST00000451079 [rs12442297] | | 0.11 | 1 | 0.68 |
| p@chr16:72108093..72108100,+ [rs2000999] | | 0.11 | 1 | 0.68 |
| enhancer@chr8:19518773-19518959 [rs4921664] | | 0.09 | 1 | 0.74 |
| | rs4921664:rs7001715 D'=1.0; $r^2$=0.66. p@chr8:19507328..19507329,- [rs7001715] | 0.05 | 1 | 0.82 |
| p@chr22:38572651..38572665,+ [rs133017] | | 0.08 | 1 | 0.77 |
| p@chr16:81610493..81610521,+ [rs11642655] | | 0.07 | 1 | 0.79 |
| enhancer@chr8:59311673-59312115 [rs1030431] | | 0.07 | 1 | 0.79 |
| enhancer@chr15:44289112-44289501 [rs6493092] | | 0.07 | 1 | 0.79 |
| enhancer@chr3:170695585-170695919 [rs7635100] | | 0.05 | 1 | 0.82 |
| enhancer@chr11:61746281-61746413 [rs10792320] | | 0.05 | 1 | 0.83 |
| p@chr2:28524175..28524215,+ [rs12621972] | | 0.04 | 1 | 0.86 |
| enhancer@chr6:127481688-127482073 [rs9491700] | | 0.04 | 1 | 0.86 |
| enhancer@chr2:227172292-227172683 [rs2713556] | | 0.02 | 1 | 0.91 |
| enhancer@chr15:44431053-44431468 [rs2555389] | | 0.01 | 1 | 0.94 |
| p8@MAPK10 [rs2869433] | | 0.0 | 1 | 1.0 |

| Cell type | RRA p | FDR |
|---|---|---|
| small intestine adult pool1 | 2.4e-06 | 0 |
| colon adult pool1 | 0.00099 | 0 |
| small intestine fetal | 0.0036 | 0 |
| duodenum fetal donor1 tech | 3.2e-05 | 0.015 |

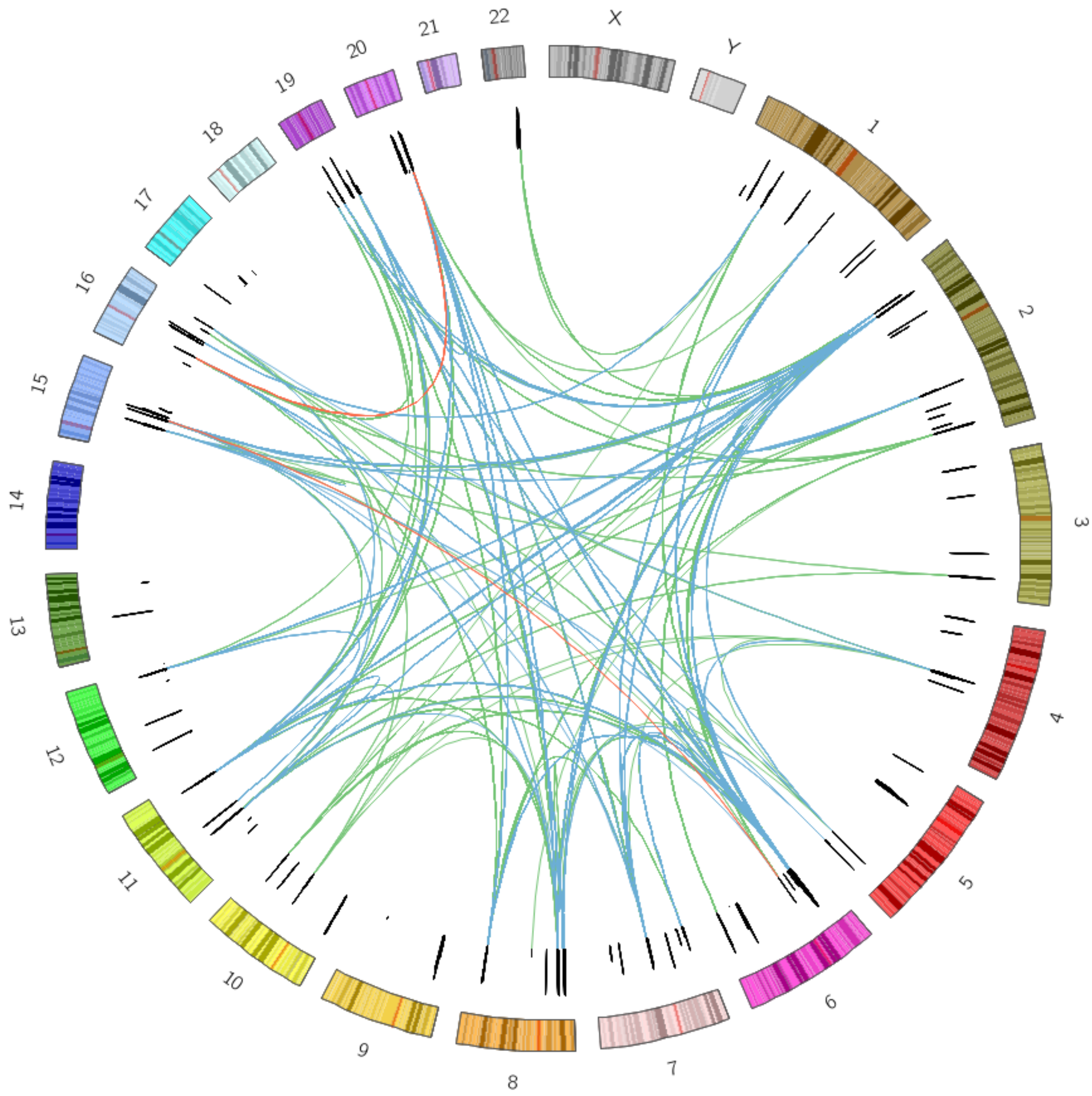


Figure 6: Circular plot of coexpression links between different locations on the genome (Triglycerides). Colour indicates $log_{10}(p)$: red>3, blue> 2, green> 1.5). See main manuscript for full explanation.

# 7 Height

| Top promoter[SNPs in top promoter] | Linkage | Corrected coexpression score | Bonferroni-corrected p-value | FDR |
|---|---|---|---|---|
| p3@RPL17 [rs3814888] | | 0.68 | 0 | 0.0 |
| | rs3814888:rs7506517 D'=1.0; $r^2$=0.57. enhancer@chr18:46946352-46946456 [rs7506517] | 0.05 | 1 | 0.91 |
| p1@CENPW [rs9388486] | | 0.67 | 0 | 0.0 |
| p1@TCF19 [rs720468, rs3130456] | | 0.65 | 0 | 0.0 |
| p1@RPL35P2 [rs6457769] | | 0.62 | 0 | 0.0 |
| p1@PSMC3 [rs7948705] | | 0.6 | 0 | 0.0 |
| | rs7948705:rs3740689 D'=1.0; $r^2$=0.58. p@chr11:47380465..47380487,- [rs3740689] | 0.02 | 1 | 0.98 |
| p@chr1:184021069..184021077,+ [rs2274432] | | 0.58 | 0 | 0.0 |
| p2@CPNE1, p2@RBM12 [rs6119636] | | 0.56 | 0 | 0.0 |
| p@chr17:47039347..47039351,+ [rs2291725, rs2291726] | | 0.54 | 0 | 0.0 |
| p1@NCAPG [rs11941723, rs2074974] | | 0.54 | 0 | 0.0 |
| | rs2074974:rs4698210 D'=1.0; $r^2$=0.62. enhancer@chr4:17827662-17827866 [rs4698210] | 0.31 | 1 | 0.21 |
| p2@RQCD1 [rs500422] | | 0.52 | 0 | 0.0 |
| | rs500422:rs611203 D'=1.0; $r^2$=0.98. p2@PLCD4 [rs611203] | 0.12 | 1 | 0.76 |
| p@chr6:31595842..31595910,- [rs1046080] | | 0.51 | 0 | 0.0 |
| | rs1046080:rs3130054 D'=0.56; $r^2$=0.19. p1@MCCD1 [rs3130054] | 0.28 | 1 | 0.3 |
| p4@HIST1H4A, p4@HIST1H4B, p4@HIST1H4D, p4@HIST1H4E, p4@HIST1H4F, p4@HIST1H4H, p4@HIST1H4I, p4@HIST1H4J, p4@HIST1H4K, p4@HIST1H4L, p4@HIST2H4A, p4@HIST2H4B, p4@HIST4H4, p5@HIST1H4C [rs2393593, rs3999544] | | 0.51 | 0 | 0.0 |
| p@chr20:47679880..47679883,+ [rs6019621] | | 0.49 | 0 | 0.0 |
| p1@JMJD4 [rs2295994] | | 0.49 | 0 | 0.0 |
| p1@ZNFX1-AS1 [rs6648] | | 0.48 | 0 | 0.0 |
| p1@EXOSC5 [rs10853751] | | 0.48 | 0 | 0.0 |
| | same SNP, different promoter: chr19:41903221..41903222,+ [rs10853751] | 0.07 | 1 | 0.85 |
| p@chr2:242192880..242192896,- [rs7578199] | | 0.47 | 0 | 0.0 |
| p12@ZBTB38 [rs1863868] | | 0.47 | 0 | 0.0 |
| p@chr7:23507613..23507647,+ [rs4496867] | | 0.46 | 0 | 0.0 |
| chr16:2257216..2257230,+ [rs26862] | | 0.45 | 0.08 | 0.0 |
| p@chr9:95691523..95691530,- [rs13296126] | | 0.43 | 0.18 | 0.01 |
| | rs13296126:rs13293147 D'=0.97; $r^2$=0.6. enhancer@chr9:95727493-95728071 [rs13293147] | 0.04 | 1 | 0.93 |
| chr9:86593333..86593367,- [rs167203] | | 0.43 | 0.22 | 0.01 |
| p@chr7:92244110..92244132,- [rs42039] | | 0.43 | 0.3 | 0.01 |
| p@chr6:28926723..28926729,+ [rs6901599] | | 0.41 | 0.62 | 0.03 |
| p@chr4:145568170..145568180,- [rs7689420] | | 0.41 | 0.73 | 0.03 |
| enhancer@chr7:46431809-46432084 [rs12534091] | | 0.39 | 1 | 0.04 |
| | rs12534091:rs13231201 D'=1.0; $r^2$=0.85. p@chr7:46385081..46385087,+ [rs13231201, rs13233996];rs12534091:rs13233996 D'=1.0; $r^2$=0.85. p@chr7:46385081..46385087,+ [rs13231201, rs13233996] | 0.24 | 1 | 0.41 |
| p1@ENST00000408001 [rs2764205] | | 0.39 | 1 | 0.04 |
| | rs2764205:rs3800457 D'=1.0; $r^2$=0.96. enhancer@chr6:34663389-34663600 [rs3800457] | 0.16 | 1 | 0.62 |
| p@chr17:59485435..59485452,+ [rs2240736] | | 0.39 | 1 | 0.05 |
| chr11:75277935..75277984,+ [rs651581] | | 0.39 | 1 | 0.05 |
| enhancer@chr2:241860652-241861096 [rs10207380] | | 0.37 | 1 | 0.06 |
| | rs10207380:rs7591322 D'=1.0; $r^2$=0.9. enhancer@chr2:241899989-241900661 [rs7591322] | 0.06 | 1 | 0.88 |
| enhancer@chr15:74226590-74227025 [rs2028386, rs4337252] | | 0.37 | 1 | 0.07 |
| p1@ENST00000438379 [rs1182175] | | 0.37 | 1 | 0.07 |
| p3@SLC38A9 [rs6450345] | | 0.36 | 1 | 0.08 |
| | rs6450345:rs2408204 D'=1.0; $r^2$=0.91. p4@SLC38A9 [rs2408204] | 0.27 | 1 | 0.31 |
| p2@MFAP2 [rs9435732] | | 0.35 | 1 | 0.09 |
| | rs9435732:rs732679 D'=0.64; $r^2$=0.26. chr1:17371735..17371757,- [rs732679, rs10887994];rs9435732:rs10887994 D'=0.95; $r^2$=0.35. chr1:17371735..17371757,- [rs732679, rs10887994] | 0.13 | 1 | 0.72 |
| p@chr13:92001646..92001654,+ [rs4284505] | | 0.35 | 1 | 0.09 |
| p1@ENST00000544890 [rs6633] | | 0.35 | 1 | 0.1 |
| | rs6633:rs12817892 D'=1.0; $r^2$=1.0. p1@ENST00000545406 [rs12817892] | 0.09 | 1 | 0.83 |
| enhancer@chr13:51103280-51103738 [rs536338] | | 0.35 | 1 | 0.1 |
| chr6:81442621..81442640,+ [rs11756729] | | 0.34 | 1 | 0.12 |
| p@chr15:84408895..84408904,+ [rs1426160] | | 0.34 | 1 | 0.12 |
| chr6:32148013..32148032,+ [rs3134943] | | 0.33 | 1 | 0.15 |
| chr6:31237739..31237758,+ [rs9264606, rs9264608] | | 0.33 | 1 | 0.18 |
| p@chr5:33229308..33229311,- [rs7443939] | | 0.32 | 1 | 0.19 |
| p3@PMPCA [rs3812584] | | 0.32 | 1 | 0.19 |
| | same SNP, different promoter: p4@PMPCA [rs3812584] | 0.08 | 1 | 0.84 |
| enhancer@chr20:34471781-34472424 [rs3787173] | | 0.31 | 1 | 0.21 |
| | rs3787173:rs11699815 D'=1.0; $r^2$=1.0. p7@SCAND1 [rs11699815] | 0.11 | 1 | 0.8 |
| p6@ACSS2 [rs3818273] | | 0.31 | 1 | 0.21 |
| | rs3818273:rs6120778 D'=1.0; $r^2$=0.75. p9@MYH7B [rs6120778] | 0.0 | 1 | 1.0 |
| p@chr5:134364600..134364638,- [rs479632] | | 0.31 | 1 | 0.21 |
| p@chr5:32829061..32829092,+ [rs7733331] | | 0.31 | 1 | 0.23 |
| enhancer@chr19:2182828-2183132 [rs12609327] | | 0.3 | 1 | 0.24 |
| enhancer@chr10:80918312-80918688 [rs779932, rs779933] | | 0.3 | 1 | 0.24 |
| p3@ANP32E [rs6679147] | | 0.3 | 1 | 0.26 |
| p4@EHMT2 [rs7887] | | 0.29 | 1 | 0.27 |
| | rs7887:rs2072633 D'=0.74; $r^2$=0.19. p37@CFB [rs2072633] | 0.1 | 1 | 0.8 |
| p5@NARFL [rs12597563] | | 0.29 | 1 | 0.27 |
| p5@ITCH [rs6579167] | | 0.29 | 1 | 0.27 |
| p@chr5:131541116..131541121,- [rs10075459] | | 0.29 | 1 | 0.27 |

| | | | | |
|---|---|---|---|---|
| enhancer@chr6:7692548-7692711 [rs7756651] | | 0.29 | 1 | 0.28 |
| p@chr6:7786714..7786717,+ [rs5009024] | | 0.28 | 1 | 0.28 |
| p@chr20:33734472..33734476,- [rs1415771] | | 0.28 | 1 | 0.3 |
| enhancer@chr3:136492102-136492187 [rs9819856] | | 0.28 | 1 | 0.3 |
| p3@TNPO1 [rs34651] | | 0.28 | 1 | 0.3 |
| enhancer@chr11:12697923-12698543 [rs7926971] | | 0.28 | 1 | 0.3 |
| p@chr10:81113959..81113978,+ [rs7332] | | 0.27 | 1 | 0.31 |
| enhancer@chr15:99214674-99215061 [rs2311767] | | 0.27 | 1 | 0.32 |
| enhancer@chr3:13555627-13555924 [rs2597513, rs2655226] | | 0.27 | 1 | 0.32 |
| p@chr12:123616405..123616412,- [rs1727294] | | 0.27 | 1 | 0.32 |
| p10@SCUBE3 [rs2071920] | | 0.26 | 1 | 0.33 |
| | rs2071920:rs6899744 D'=1.0; $r^2$=0.05. p@chr6:35286250..35286262,+ [rs6899744] | 0.24 | 1 | 0.41 |
| enhancer@chr5:54886412-54886788 [rs1983189] | | 0.26 | 1 | 0.35 |
| | rs1983189:rs3936310 D'=0.89; $r^2$=0.64. enhancer@chr5:54896440-54896979 [rs3936310] | 0.13 | 1 | 0.72 |
| p5@NOS3 [rs743507] | | 0.26 | 1 | 0.35 |
| p@chr17:30326770..30326783,+ [rs537166] | | 0.25 | 1 | 0.36 |
| enhancer@chr5:32768263-32768639 [rs3792752] | | 0.25 | 1 | 0.36 |
| enhancer@chr1:23448967-23449247 [rs1571466] | | 0.25 | 1 | 0.37 |
| | rs1571466:rs2806561 D'=1.0; $r^2$=1.0. p3@LUZP1 [rs2806561] | 0.13 | 1 | 0.73 |
| p@chr6:32575596..32575598,- [rs9461776] | | 0.24 | 1 | 0.39 |
| enhancer@chr9:95288173-95288480 [rs4744137] | | 0.24 | 1 | 0.39 |
| p@chr19:7184803..7184814,- [rs891088] | | 0.24 | 1 | 0.41 |
| p@chr6:142702634..142702638,+ [rs7757571] | | 0.24 | 1 | 0.41 |
| p@chr1:172134755..172134764,+ [rs537444] | | 0.23 | 1 | 0.42 |
| | same SNP, different promoter: p@chr1:172134736..172134739,+ [rs537444] | 0.1 | 1 | 0.8 |
| p@chr12:56727954..56727978,+ [rs2371494] | | 0.23 | 1 | 0.42 |
| enhancer@chr8:135628946-135629314 [rs7010948, rs7011157] | | 0.23 | 1 | 0.44 |
| | rs7010948:rs894344 D'=0.89; $r^2$=0.59. p@chr8:135612764..135612786,- [rs894344];rs7011157:rs894344 D'=0.89; $r^2$=0.59. p@chr8:135612764..135612786,- [rs894344] | 0.18 | 1 | 0.56 |
| p1@C20orf173 [rs6060450] | | 0.23 | 1 | 0.44 |
| p13@TGFB2 [rs991967] | | 0.22 | 1 | 0.46 |
| p@chr20:33356385..33356390,- [rs2295353] | | 0.22 | 1 | 0.47 |
| p@chr13:33232408..33232414,+ [rs2301393, rs7335546] | | 0.21 | 1 | 0.48 |
| p@chr12:93967885..93967910,+ [rs3782415] | | 0.21 | 1 | 0.49 |
| enhancer@chr5:131406243-131406684 [rs721121] | | 0.21 | 1 | 0.49 |
| p@chr19:3434311..3434347,+ [rs2074977] | | 0.21 | 1 | 0.49 |
| enhancer@chr1:77980165-77980378 [rs12729914] | | 0.21 | 1 | 0.49 |
| enhancer@chr2:25457688-25458068 [rs11695471] | | 0.21 | 1 | 0.49 |
| | rs11695471:rs7594432 D'=1.0; $r^2$=0.35. enhancer@chr2:25482494-25482888 [rs7594432] | 0.17 | 1 | 0.6 |
| p13@ESR1 [rs2077647] | | 0.2 | 1 | 0.5 |
| enhancer@chr7:2749963-2750288 [rs7798875] | | 0.2 | 1 | 0.5 |
| enhancer@chr6:131327805-131328036 [rs6921207] | | 0.2 | 1 | 0.5 |
| p@chr3:171965372..171965436,+ [rs1039027] | | 0.2 | 1 | 0.52 |
| enhancer@chr12:94178297-94178734 [rs10859579] | | 0.19 | 1 | 0.53 |
| p@chr12:11898277..11898290,+ [rs1009954] | | 0.19 | 1 | 0.54 |
| p10@FAM134A [rs3210652] | | 0.19 | 1 | 0.54 |
| enhancer@chr14:68699331-68699744 [rs1314913] | | 0.19 | 1 | 0.56 |
| p@chr18:46582506..46582523,+ [rs16950294, rs16950298, rs16950303] | | 0.18 | 1 | 0.59 |
| enhancer@chr20:6594261-6594618 [rs6038557, rs6054392] | | 0.18 | 1 | 0.59 |
| enhancer@chr2:88899554-88899981 [rs1913671] | | 0.17 | 1 | 0.59 |
| p@chr6:26500524..26500540,+ [rs13194984] | | 0.17 | 1 | 0.6 |
| enhancer@chr17:61639369-61639739 [rs9906747] | | 0.17 | 1 | 0.61 |
| p2@SLFNL1 [rs17357954] | | 0.17 | 1 | 0.61 |
| p@chr6:35765153..35765158,+ [rs2766597] | | 0.17 | 1 | 0.61 |
| p@chr6:32412808..32412821,- [rs3135388] | | 0.16 | 1 | 0.61 |
| p@chr15:84295121..84295125,+ [rs2585070] | | 0.16 | 1 | 0.62 |
| enhancer@chr13:51210187-51210463 [rs7991818] | | 0.16 | 1 | 0.62 |
| p4@SLC3A1 [rs3738985] | | 0.16 | 1 | 0.63 |
| enhancer@chr2:233181304-233181393 [rs6743962] | | 0.16 | 1 | 0.63 |
| enhancer@chr20:47471567-47471956 [rs6012541, rs6095329] | | 0.15 | 1 | 0.65 |
| | rs6012541:rs752421 D'=0.98; $r^2$=0.95. enhancer@chr20:47434951-47435381 [rs752421];rs6095329:rs752421 D'=0.98; $r^2$=0.95. enhancer@chr20:47434951-47435381 [rs752421] | 0.07 | 1 | 0.85 |
| enhancer@chr11:65248969-65249218 [rs1787666] | | 0.15 | 1 | 0.66 |
| p@chr7:20392799..20392811,+ [rs2214442] | | 0.13 | 1 | 0.72 |
| p@chr9:98245687..98245692,- [rs17369383] | | 0.13 | 1 | 0.73 |
| enhancer@chr15:70047760-70048370 [rs10152590, rs10152591] | | 0.12 | 1 | 0.76 |
| enhancer@chr2:25136821-25136958 [rs10203386] | | 0.12 | 1 | 0.77 |
| | rs10203386:rs9631062 D'=0.96; $r^2$=0.52. p@chr2:25110228..25110248,+ [rs9631062] | 0.09 | 1 | 0.81 |
| p@chr1:184128142..184128173,+ [rs6424925] | | 0.11 | 1 | 0.77 |
| chr5:168233584..168233600,- [rs17553840] | | 0.1 | 1 | 0.8 |
| | rs17553840:rs17635284 D'=1.0; $r^2$=1.0. p@chr5:168246378..168246386,+ [rs17635284] | 0.02 | 1 | 0.98 |
| p9@ITIH4 [rs13072536] | | 0.1 | 1 | 0.8 |
| p1@C2CD4A [rs8039105] | | 0.1 | 1 | 0.8 |
| p@chr6:76415669..76415672,+ [rs9343320] | | 0.1 | 1 | 0.8 |
| p4@MICAL1 [rs2277113] | | 0.1 | 1 | 0.81 |
| | rs2277113:rs9398202 D'=1.0; $r^2$=0.88. enhancer@chr6:109780095-109780443 [rs9398202] | 0.07 | 1 | 0.85 |
| enhancer@chr3:135861520-135861675 [rs9881400] | | 0.09 | 1 | 0.84 |
| p@chr1:149875845..149875874,- [rs7534365] | | 0.08 | 1 | 0.84 |
| enhancer@chr11:48102794-48103200 [rs6485807] | | 0.08 | 1 | 0.84 |
| p8@PRKG2 [rs788861] | | 0.08 | 1 | 0.84 |
| p@chr5:131675781..131675785,+ [rs272872] | | 0.08 | 1 | 0.84 |
| p@chr6:6884633..6884678,- [rs4960263] | | 0.08 | 1 | 0.84 |
| enhancer@chr20:34689096-34689267 [rs2746102] | | 0.07 | 1 | 0.86 |
| p@chr3:11643506..11643527,- [rs2276749] | | 0.06 | 1 | 0.87 |

| | | | | |
|---|---|---|---|---|
| p@chr2:219610047..219610074,- [rs2272189] | | 0.06 | 1 | 0.88 |
| p@chr5:179737830..179737831,- [rs888926] | | 0.05 | 1 | 0.91 |
| enhancer@chr6:34358644-34359014 [rs3798564] | | 0.05 | 1 | 0.91 |
| p2@ENST00000473753 [rs11049416] | | 0.05 | 1 | 0.91 |
| enhancer@chr7:28122864-28123088 [rs10951191] | | 0.04 | 1 | 0.93 |
| p6@CCDC91 [rs10843172] | | 0.04 | 1 | 0.93 |
| p@chr9:139118938..139118947,- [rs10858250] | | 0.04 | 1 | 0.93 |
| p3@HCG22 [rs4713429, rs9262615] | | 0.04 | 1 | 0.93 |
| | rs4713429:rs7744253 D'=0.94; $r^2$=0.86. p@chr6:31023284..31023287,- [rs7744253];rs9262615:rs7744253 D'=0.92; $r^2$=0.84. p@chr6:31023284..31023287,- [rs7744253] | 0.03 | 1 | 0.96 |
| chr17:21279520..21279522,+ [rs7213608] | | 0.02 | 1 | 0.97 |
| p2@SOCS5 [rs3829835] | | 0.01 | 1 | 0.99 |
| p@chr20:34820071..34820089,+ [rs3813918] | | 0.01 | 1 | 0.99 |
| p@chr15:100749314..100749319,- [rs5026360] | | 0.0 | 1 | 1.0 |

| Cell type | RRA p | FDR |
|---|---|---|
| Skeletal Muscle Cells | 6.4e-10 | 0 |
| K562 erythroblastic leukemia response to hemin 00hr45min | 1.6e-07 | 0 |
| COBLa rinderpest infection 06hr | 7.2e-07 | 0 |
| Fibroblast skin normal(cytoplasmic) | 1.1e-06 | 0 |
| K562 erythroblastic leukemia response to hemin 03hr00min | 3.7e-06 | 0 |
| COBLa rinderpest infection 24hr | 4.3e-06 | 0 |
| Hair Follicle Dermal Papilla Cells | 1.5e-05 | 0 |
| Preadipocyte subcutaneous | 3.5e-05 | 0 |
| Schwann Cells | 3.9e-05 | 0 |
| iPS differentiation to neuron downsyndrome donor C11CCL54 day00 | 4.1e-05 | 0 |
| H1 embryonic stem cells differentiation to CD34 HSC day00 | 5.8e-05 | 0 |
| Preadipocyte visceral | 6.4e-05 | 0 |
| Mesenchymal Stem Cells bone marrow | 7.7e-05 | 0 |
| Skeletal muscle cells differentiated into Myotubes multinucleated | 0.00014 | 0 |
| iPS differentiation to neuron control donor C11CRL2429 day00 | 0.00015 | 0 |
| Fibroblast Cardiac | 0.0002 | 0 |
| Renal Glomerular Endothelial Cells | 0.00024 | 0 |
| 293SLAM rinderpest infection 00hr | 0.00035 | 0 |
| Osteoblast | 0.00035 | 0 |
| Skeletal Muscle Satellite Cells | 0.00061 | 0 |
| Fibroblast Dermal | 0.00071 | 0 |
| Hepatic Stellate Cells (lipocyte) | 0.00093 | 0 |
| Smooth Muscle Cells Bronchial | 0.0078 | 0 |
| Mammary Epithelial Cell | 0.0089 | 0 |
| Prostate Stromal Cells | 0.018 | 0 |
| Endothelial Cells Artery | 0.022 | 0 |
| Meningeal Cells | 0.035 | 0 |
| Smooth Muscle Cells Tracheal | 0.038 | 0 |
| Smooth muscle cells airway asthmatic | 1.4e-05 | 0.00021 |
| K562 erythroblastic leukemia response to hemin 12hr | 0.00013 | 0.00021 |
| Ciliary Epithelial Cells | 0.0062 | 0.00021 |
| Hep2 cells treated with Streptococci strain 5448 | 0.0072 | 0.00021 |
| Renal Epithelial Cells | 0.0079 | 0.00021 |
| Smooth Muscle Cells Umbilical Artery | 0.028 | 0.00021 |
| mesenchymal precursor cell adipose | 0.028 | 0.00021 |
| Fibroblast Aortic Adventitial | 0.047 | 0.00021 |
| Mesenchymal Stem Cells adipose | 5.7e-06 | 0.00037 |
| K562 erythroblastic leukemia response to hemin day04 | 2.5e-05 | 0.00037 |
| Alveolar Epithelial Cells | 0.0003 | 0.00037 |
| COBLa rinderpest infection 12hr | 0.0022 | 0.00037 |
| Smooth Muscle Cells Umbilical Vein | 0.0058 | 0.00037 |
| K562 erythroblastic leukemia response to hemin 00hr00min | 4.7e-07 | 0.00051 |
| HES3GFP Embryonic Stem cells cardiomyocytic induction | 0.00021 | 0.00051 |
| COBLa rinderpest infection 00hr | 0.00029 | 0.00051 |
| lymph endothelial cells VEGF 00hr15min | 0.02 | 0.00051 |
| Fibroblast Gingival(periodontitis) | 0.0082 | 0.00066 |
| K562 erythroblastic leukemia response to hemin day03 | 0.00019 | 0.00081 |
| Corneal Epithelial Cells | 0.0016 | 0.00086 |
| Endothelial Cells Vein | 0.0054 | 0.00086 |
| aorticSMC responsetoIL1b immediate early 00hr15min | 0.0074 | 0.00086 |
| Smooth Muscle Cells Brachiocephalic | 0.011 | 0.00086 |
| Fibroblast Periodontal Ligament | 0.013 | 0.00086 |
| Saos2calc 00hr15min | 0.04 | 0.00086 |
| K562 erythroblastic leukemia response to hemin 06hr | 2.5e-05 | 0.00095 |
| COBLa rinderpestC infection 24hr | 4.4e-05 | 0.00095 |
| iPS differentiation to neuron control donor C32CRL1502 day00 | 0.00022 | 0.0012 |
| Retinal Pigment Epithelial Cells | 0.018 | 0.0012 |
| 293SLAM rinderpest infection 12hr | 0.0022 | 0.0013 |
| K562 erythroblastic leukemia response to hemin day02 | 9.9e-05 | 0.0013 |
| mesenchymalstemcells(adiposederived)adipogenicinduction 00hr30min | 0.00049 | 0.0013 |
| Fibroblast Aortic Adventitial(cytoplasmic) | 0.016 | 0.0013 |
| mesenchymal precursor cell cardiac | 0.037 | 0.0013 |
| 293SLAM rinderpest infection 06hr | 0.0035 | 0.0016 |
| Tracheal Epithelial Cells | 0.012 | 0.0017 |
| iPS differentiation to neuron downsyndrome donor C18CCL54 day06 | 0.00011 | 0.0017 |
| mesenchymal precursor cell ovarian cancer right ovary | 0.0021 | 0.0019 |
| 293SLAM rinderpest infection 24hr | 3.4e-05 | 0.002 |
| K562 erythroblastic leukemia response to hemin 01hr00min | 0.0019 | 0.0026 |
| K562 erythroblastic leukemia response to hemin 00hr30min | 0.00021 | 0.0027 |
| HES3GFP Embryonic Stem cells cardiomyocytic induction day08 | 0.0016 | 0.0027 |
| K562 erythroblastic leukemia response to hemin 01hr40min | 0.00078 | 0.0031 |
| Placental Epithelial Cells | 0.014 | 0.0031 |
| HES3GFP Embryonic Stem cells cardiomyocytic induction day05 | 0.0085 | 0.0031 |
| iPS differentiation to neuron downsyndrome donor C18CCL54 day00 | 0.0088 | 0.0031 |
| K562 erythroblastic leukemia response to hemin 03hr30min | 0.0029 | 0.0032 |
| COBLa rinderpestC infection 12hr | 0.0084 | 0.0035 |
| iPS differentiation to neuron downsyndrome donor C11CCL54 day18 | 0.015 | 0.0038 |
| HES3GFP Embryonic Stem cells cardiomyocytic induction day03 | 0.00033 | 0.0039 |
| iPS differentiation to neuron control donor C11CRL2429 day18 | 0.038 | 0.0044 |
| Mesenchymal Stem Cells umbilical | 0.022 | 0.0056 |
| Bronchial Epithelial Cell | 0.00017 | 0.0061 |
| Myoblast differentiation to myotubes day00 Duchenne Muscular Dystrophy | 0.00011 | 0.0069 |

| | | |
|---|---|---|
| K562 erythroblastic leukemia response to hemin 00hr15min | 0.00087 | 0.0076 |
| Fibroblast Gingival | 0.004 | 0.011 |
| K562 erythroblastic leukemia response to hemin 04hr | 0.0049 | 0.011 |
| HES3GFP Embryonic Stem cells cardiomyocytic induction day07 | 0.0051 | 0.011 |
| K562 erythroblastic leukemia response to hemin 01hr20min | 0.01 | 0.012 |
| Hepatic Sinusoidal Endothelial Cells | 0.045 | 0.012 |
| hIPS | 0.0013 | 0.012 |
| ARPE19 EMT induced with TGFbeta and TNFalpha 24hr00min | 4.7e-07 | 0.015 |
| K562 erythroblastic leukemia response to hemin 02hr30min | 0.016 | 0.017 |
| HES3GFP Embryonic Stem cells cardiomyocytic induction day04 | 0.024 | 0.018 |
| ARPE19 EMT induced with TGFbeta and TNFalpha 08hr00min | 0.02 | 0.018 |
| K562 erythroblastic leukemia response to hemin 02hr00min | 0.031 | 0.019 |
| ARPE19 EMT induced with TGFbeta and TNFalpha 01hr40min | 0.037 | 0.021 |
| HES3GFP Embryonic Stem cells cardiomyocytic induction day01 | 0.027 | 0.03 |
| HES3GFP Embryonic Stem cells cardiomyocytic induction day06 | 0.044 | 0.034 |

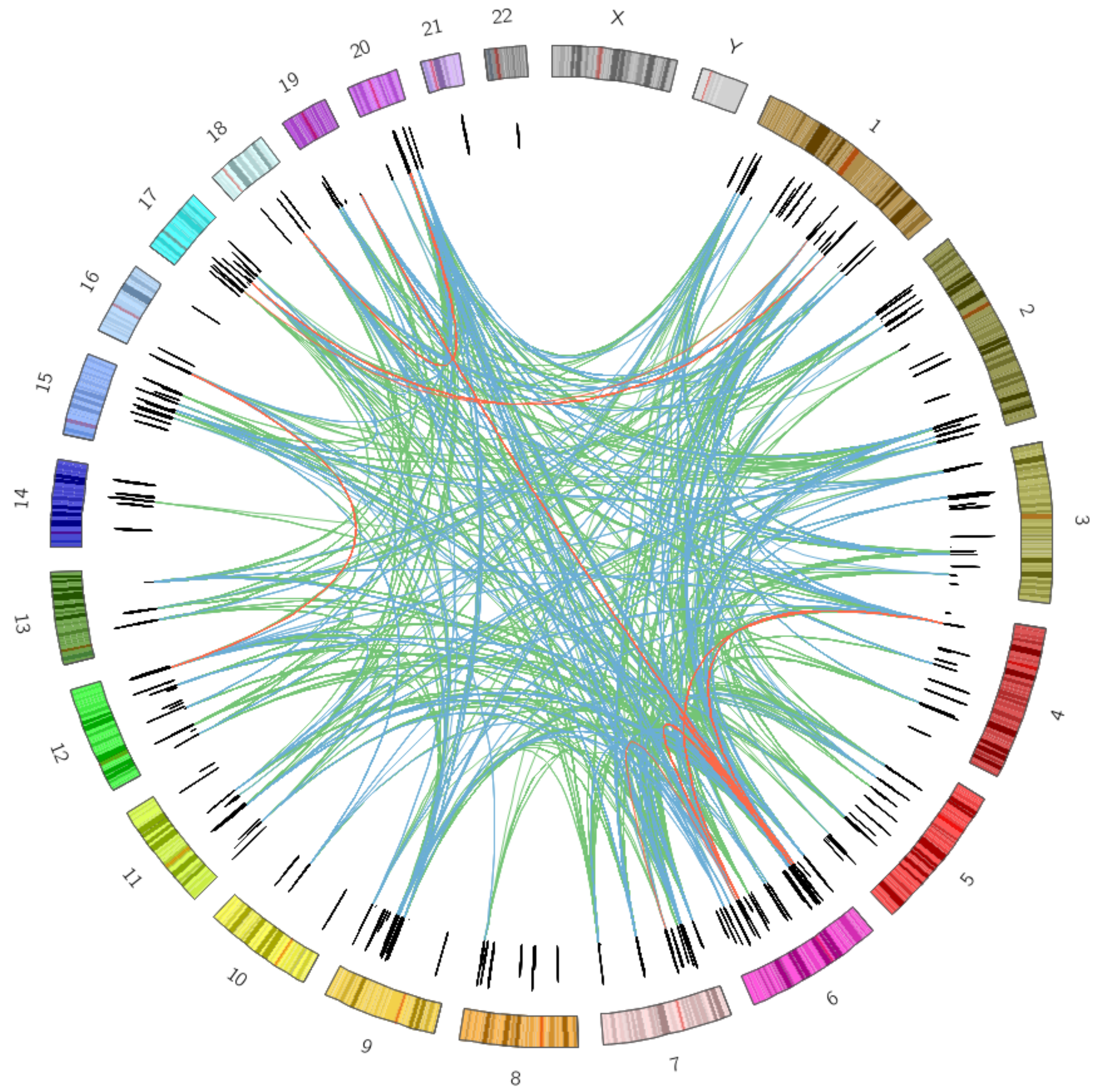


Figure 7: Circular plot of coexpression links between different locations on the genome (Height). Colour indicates $log_{10}(p)$: red>3, blue> 2, green> 1.5). See main manuscript for full explanation.

# 8 Diastolic Blood Pressure

| Top promoter[SNPs in top promoter] | Linkage | Corrected coexpression score | Bonferroni-corrected p-value | FDR |
|---|---|---|---|---|
| p@chr11:16916978..16916996,- [rs11024074] | | 0.58 | 0.29 | 0.29 |
| enhancer@chr7:107939769-107940082 [rs11561991] | | 0.49 | 0.59 | 0.3 |
| p@chr3:27490147..27490174,+ [rs13096477] | | 0.43 | 0.97 | 0.32 |
| p4@SH2B3 [rs739496] | | 0.36 | 1 | 0.37 |
| p@chr10:63524522..63524527,- [rs1530440] | | 0.32 | 1 | 0.37 |
| p@chr6:26104437..26104461,- [rs198851] | | 0.31 | 1 | 0.37 |
| p@chr12:90090838..90090842,- [rs12230074, rs11105378] | | 0.22 | 1 | 0.52 |
| p11@MTHFR [rs13306561] | | 0.21 | 1 | 0.52 |
| p@chr4:81208116..81208130,+ [rs3733336] | | 0.14 | 1 | 0.65 |
| p@chr12:112212004..112212016,+ [rs2238151] | | 0.1 | 1 | 0.72 |
| p3@NT5C2 [rs11191582] | | 0.08 | 1 | 0.72 |
| p8@ATXN2 [rs695871] | | 0.03 | 1 | 0.85 |
| p14@CSK [rs2168519, rs8033381] | | 0.02 | 1 | 0.85 |
| enhancer@chr11:16357706-16357868 [rs12798854] | | 0.0 | 1 | 1.0 |

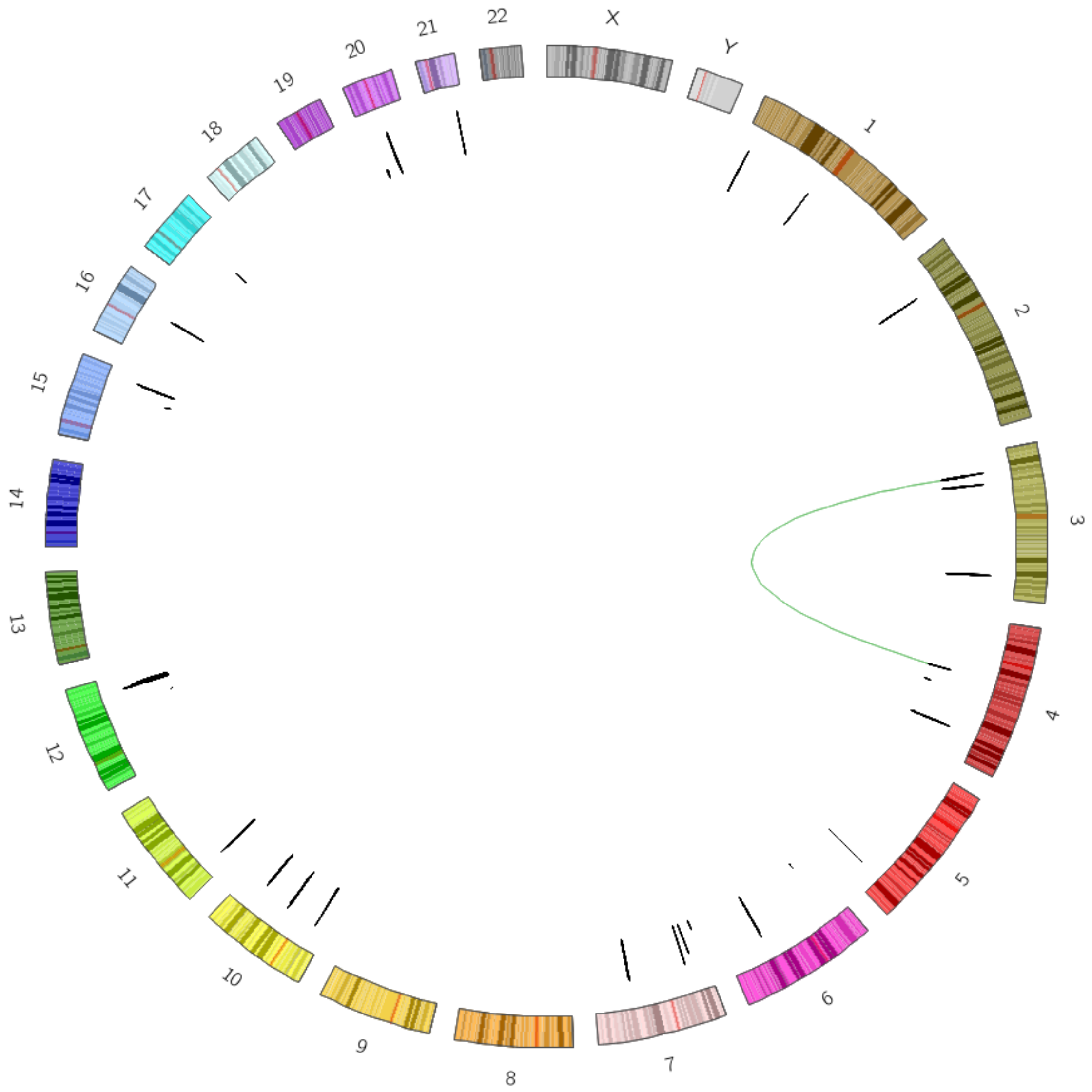


Figure 8: Circular plot of coexpression links between different locations on the genome (Diastolic Blood Pressure). Colour indicates $log_{10}(p)$: red>3, blue> 2, green> 1.5). See main manuscript for full explanation.

# 9 Systolic Blood Pressure

| Top promoter[SNPs in top promoter] | Linkage | Corrected coexpression score | Bonferroni-corrected p-value | FDR |
|---|---|---|---|---|
| p@chr1:11905830..11905839,- [rs5068] | | 0.54 | 0.11 | 0.09 |
| enhancer@chr11:10350347-10350728 [rs7129220] | | 0.46 | 0.24 | 0.09 |
| p@chr11:16916978..16916996,- [rs11024074] | | 0.44 | 0.26 | 0.09 |
| p@chr5:32829061..32829092,+ [rs7733331] | | 0.37 | 0.77 | 0.19 |
| p@chr10:75409754..75409787,- [rs9664184, rs12247028] | | 0.27 | 1 | 0.34 |
| p14@CSK [rs2168519, rs8033381] | | 0.18 | 1 | 0.48 |
| p@chr10:104905957..104905982,- [rs11191580] | | 0.17 | 1 | 0.48 |
| enhancer@chr20:57740587-57741260 [rs6026742] | | 0.12 | 1 | 0.57 |
| p@chr10:104591351..104591362,+ [rs17115100] | | 0.09 | 1 | 0.65 |
| p@chr17:43195790..43195798,- [rs3744760] | | 0.06 | 1 | 0.69 |
| p@chr12:90090838..90090842,- [rs11105378, rs12230074] | | 0.02 | 1 | 0.79 |
| p5@FURIN [rs4932178] | | 0.02 | 1 | 0.79 |
| enhancer@chr10:104769429-104769711 [rs11191511] | | 0.0 | 1 | 1.0 |

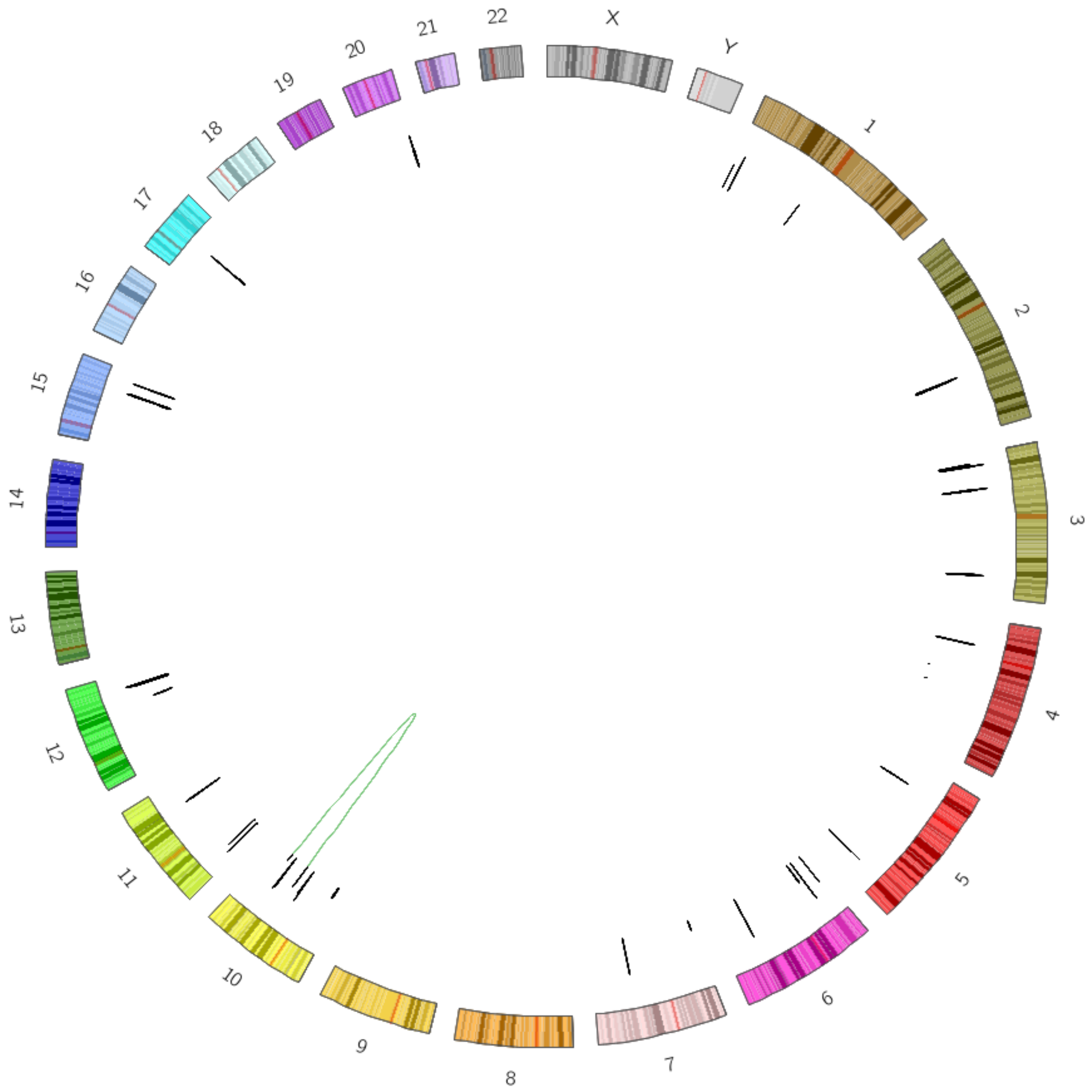


Figure 9: Circular plot of coexpression links between different locations on the genome (Systolic Blood Pressure). Colour indicates $log_{10}(p)$: red>3, blue> 2, green> 1.5). See main manuscript for full explanation.
